# An ultrasound-activatable platinum prodrug for sono-sensitized chemotherapy

**DOI:** 10.1126/sciadv.adg5964

**Published:** 2023-06-21

**Authors:** Gongyuan Liu, Yachao Zhang, Houzong Yao, Zhiqin Deng, Shu Chen, Yue Wang, Wang Peng, Guohan Sun, Man-Kit Tse, Xianfeng Chen, Jianbo Yue, Yung-Kang Peng, Lidai Wang, Guangyu Zhu

**Affiliations:** ^1^ Department of Chemistry, City University of Hong Kong, Hong Kong SAR, P.R. China.; ^2^ City University of Hong Kong Shenzhen Research Institute, Shenzhen 518057, P.R. China.; ^3^ Department of Biomedical Engineering, City University of Hong Kong, Hong Kong SAR, P.R. China.; ^4^ Department of Biomedical Sciences, City University of Hong Kong, Hong Kong SAR, P.R. China.; ^5^ School of Engineering, Institute for Bioengineering, University of Edinburgh, UK.; ^6^ Division of Natural and Applied Sciences, Duke Kunshan University, Kunshan 215316, P.R. China.

## Abstract

Despite the great success achieved by photoactivated chemotherapy, eradicating deep tumors using external sources with high tissue penetration depth remains a challenge. Here, we present cyaninplatin, a paradigm of Pt(IV) anticancer prodrug that can be activated by ultrasound in a precise and spatiotemporally controllable manner. Upon sono-activation, mitochondria-accumulated cyaninplatin exhibits strengthened mitochondrial DNA damage and cell killing efficiency, and the prodrug overcomes drug resistance as a consequence of combined effects from released Pt(II) chemotherapeutics, the depletion of intracellular reductants, and the burst of reactive oxygen species, which gives rise to a therapeutic approach, namely sono-sensitized chemotherapy (SSCT). Guided by high-resolution ultrasound, optical, and photoacoustic imaging modalities, cyaninplatin realizes the overall theranostics of tumors in vivo with superior efficacy and biosafety. This work highlights the practical utility of ultrasound to precisely activate Pt(IV) anticancer prodrugs for the eradication of deep tumor lesions and broadens the biomedical uses of Pt coordination complexes.

## Teaser

A sono-activatable Pt(IV) anticancer prodrug can achieve the on-demand activation of platinum chemotherapeutics in deep tumor.

## INTRODUCTION

Through using energy-conversion chemistry, the controllable activation of prodrugs by exogenous stimuli can realize therapeutic courses with spatiotemporal accuracy. (*1*, *2*) The activatable molecular prodrugs augment the therapeutic behavior of original drugs toward location-specific treatment with reduced adverse effects (*3*, *4*). For example, photoactivatable chemotherapeutics can realize controllable activation in a spatiotemporal manner to surmount the potential issues of conventional antineoplastics (*5*–*7*). In recent years, photoactivatable Pt(IV) prodrugs have achieved great success, taking the advantages of Pt(IV) anticancer prodrugs including high stability and easy functionalization for versatile theranostic tasks (*8*–*14*). In addition, Pt(IV) scaffolds can be integrated with phototherapy, whereby photosensitization is used to generate radicals or reactive oxygen species (ROS) (*15*–*17*). However, although photoactivatable Pt(IV) prodrugs have been successfully applied, the light used for their photoactivation penetrates issues to millimeter depths, which is too shallow to realize the treatment of deep tumors. Moreover, the presence of strong optical scattering prevents precisely controlling the treatment region in deep tissue (*18*). Thus, photoactivatable prodrugs have yet to meet the demands for a noninvasive therapy capable of the precise ablation of deep tumor lesions.

Various forms of energy input, such as x-ray, magnetic field, and ultrasound, have entered clinical applications as tissue-penetrating imaging sources (*19*, *20*). Among them, ultrasound especially focused ultrasound (FUS) outstandingly appears as a “healing sound” that exhibits high penetration depth, great cost effectiveness, and superior biosafety. Ultrasound has been widely used for deep tissue imaging, therapy, and sono-responsive supramolecular- and nanodrug delivery (*21*–*26*). At present, except for a few studies where ultrasound facilitates cellular uptake and drug infiltration of Pt(II)-based chemotherapeutics in tumors (*27*–*30*), the exploration of using mechanical force to activate metal-based anticancer agents is desperately insufficient. The involvement of sono-activation process in metal-based drugs such as Pt(IV) species, whereby mechanical energy of soundwave is used to trigger specific bond scission and the reduction of Pt(IV) prodrugs, may enable the development of a therapeutic approach for inhibition or even removal of deep tumors.

**Fig. 1.**
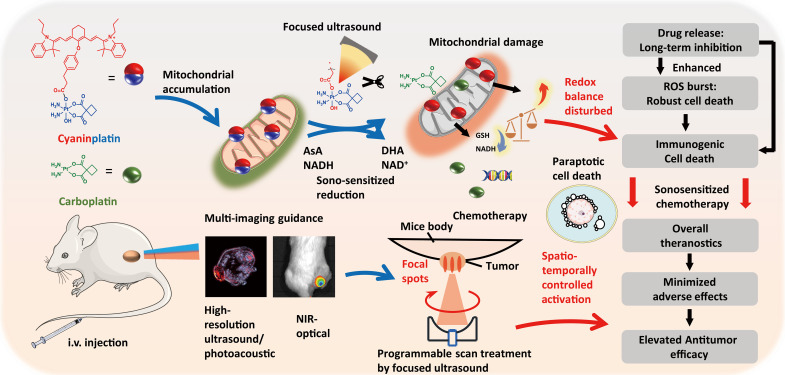
Schematic illustration of the working mechanism of cyaninplatin. Cyaninplatin can execute cancer cell killing and multimodal imaging guided sono-sensitized chemotherapy (SSCT) upon activation by focused ultrasound (FUS). i.v., intravenous.

Here, we present a molecular paradigm of ultrasound-activatable Pt(IV) prodrug that realizes the on-demand release of Pt(II) chemotherapeutics via a sono-sensitized electron transfer process (Fig. 1). Upon the treatment by a FUS system with precise and spatiotemporal control, beyond the boosted reduction of the mitochondria-targeted Pt(IV) scaffold to release carboplatin, the simultaneous depletion of intracellular reductants during the sono-activated reduction process further augments the ROS-induced damage. Such concomitant therapy not only allows for the burst release of Pt(II) therapeutics but also circumvents the antioxidant defense system to overcome drug resistance. By inducing mitochondrial dysfunction and subsequent endoplasmic reticulum (ER) stress, sono-activated cyaninplatin kills cancer cells via programmed paraptosis and strong immunogenic cell death (ICD). This prodrug also acts as a multi-imaging contrast agent that allows guidance by high-resolution ultrasound imaging, near-infrared (NIR) optical imaging, and photoacoustic computed tomography before therapeutic courses. This spatiotemporally controlled activation of cyaninplatin exhibits outstanding anticancer efficacy in a mouse model, thus accomplishing a theranostic approach, designated as sono-sensitized chemotherapy (SSCT). The application of FUS allows the activation of cyaninplatin residing in centimeter-deep tumors. The strategy presented in this study demonstrates the feasibility of activating small-molecule metal-based prodrugs by mechanical force and broadens the biomedical applicability of Pt coordination complexes, especially for the noninvasive treatment of deep tumors.

## RESULTS

### Synthesis and characterization of Pt(IV) prodrug

**Fig. 2.**
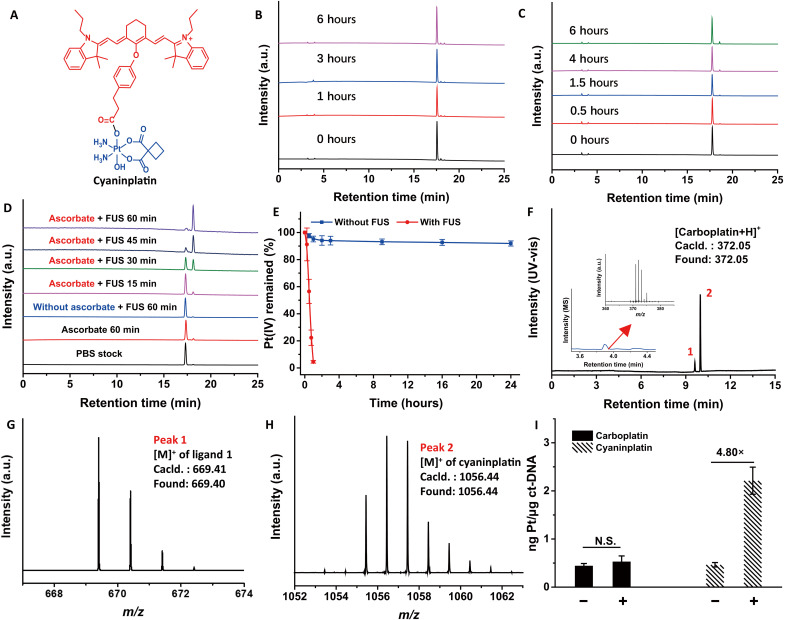
Stability and focused ultrasound (FUS)–mediated reduction of cyaninplatin. (**A**) Chemical structure of cyaninplatin. (**B**) Stability of cyaninplatin in complete RPMI 1640 culture medium containing 10% fetal bovine serum (FBS) at 37°C. (**C**) Stability of cyaninplatin in the lysate of A2780 cells at 37°C. (**D**) Reduction of cyaninplatin under sono-activation (FUS: 1.75 MHz, 4 W, 0 to 60 min) in phosphate-buffered saline (PBS) containing 5 mM ascorbate at 37°C with (**E**) corresponding reduction profile of cyaninplatin with or without FUS. (**F**) Liquid chromatography (LC) trace of reduction products of sono-activated (4 W, 45 min, in PBS buffer, pH 7.4, with 5 mM ascorbate, ultraviolet-visible (UV-vis) detector at 700 nm) cyaninplatin [insert: LC trace and mass spectrometry (MS) results of released carboplatin], with corresponding HR-MS analysis results of (**G**) peak 1 and (**H**) peak 2. (**I**) Calf thymus DNA (ct-DNA) binding of carboplatin or cyaninplatin with or without FUS activation (4 W, 60 min, in PBS buffer, pH 7.4, with 5 mM ascorbate). *t* test, N.S. not significant, mean ± SD, *n* = 3. a.u., arbitrary units; *m/z*, mass/charge ratio.

To rationally design an ultrasound-responsive theranostic Pt-coordination compound, IR780, a representative heptamethine cyanine sonosensitizer, was adopted as an ultrasound-antenna motif in a Pt(IV) moiety, which may not only facilitate electron transfer processes capable of reducing Pt(IV) center upon sono-excitation but also enable the use of optical-fluorescent and photoacoustic imaging (*31*). IR780 efficiently enters cells via organic anion-transporting polypeptides and accumulates in the mitochondria, and it is resistant to adenosine triphosphate (ATP)–binding cassette-related efflux (*32*, *33*). We used a carboplatin-based Pt(IV) scaffold owing to its enhanced stability in serum and inertness to intracellular reductants compared to cisplatin derivatives (*34*, *35*). Following this design, IR780 was first modified to obtain ligand **1** (fig. S1), which was then conjugated to *c,t*-[Pt(CBDCA)(NH_3_)_2_(OH)_2_] (CBDCA = 1,1-cyclobutane-dicarboxylate; fig. S2). The resulting Pt(IV) complex was designated as cyaninplatin (Fig. 2A), and we characterized the purified complex via ^1^H, ^13^C, and ^195^Pt nuclear magnetic resonance (NMR), ultraviolet-visible (UV-vis), fluorescence spectroscopies, and liquid chromatography–high resolution mass spectrometry (LC-HRMS) (figs. S3 to S6). Noteworthy, the strong absorption and fluorescence of ligand **1** in the NIR region (at 780 nm) persisted after conjugation with Pt (fig. S6). Therefore, cyaninplatin can also serve as a contrast agent for optical and photoacoustic imaging, enabling the monitoring of drug accumulation and diagnostic tumor imaging toward a holistic theranostic approach.

### Stability and sono-activation

Out of the primary concern for prodrugs, we evaluated the stability of cyaninplatin under various circumstances. In phosphate-buffered saline (PBS; pH 7.4), cyaninplatin remained intact after incubation for 24 hours at 37°C (fig. S7A), and the prodrug was stable in complete RPMI 1640 cell culture medium containing 10% fetal bovine serum (FBS; 98.3% remained after 6 hours; Fig. 2B) as well as in cell lysate from A2780 ovarian carcinoma cells (95.6% remained after 6 hours; Fig. 2C). These data clearly indicate the high stability of this prodrug under different physiological conditions. Henceforth, we tested the ultrasound-mediated activation using a lab-built FUS system with controllable power and steerable focus (the details are described in Materials and Methods). In the presence of sodium ascorbate (NaAsc; 5 mM in PBS, 37°C) without sono-activation, 92% of cyaninplatin remained after 24 hours (fig. S7B). In contrast, upon activation by FUS, 95% of cyaninplatin was expeditiously reduced over ultrasound treatment for 1 hour (Fig. 2, D and E). Intriguingly, in the absence of sodium ascorbate, the ultrasound-triggered reduction was negligible (<1%; Fig. 2D), indicating that an external electron donor was required for the reduction and that the process was via intermolecular electron transfer. In fresh cell lysates, FUS can also facilitate the reduction of cyaninplatin (fig. S7C). Furthermore, we confirmed the reduction product to be carboplatin and ligand **1** by LC-HRMS (Fig. 2, F to H) and ^1^H NMR (fig. S8), validating the concept of using mechanical force to activate Pt(IV) prodrugs. Meanwhile, dehydroascorbic acid was identified as the oxidized product after sono-reduction of cyaninplatin in the presence of ascorbate, proving that ascorbate is an intermolecular electron donor (fig. S9). When incubated with calf thymus DNA (ct-DNA), compared with carboplatin, cyaninplatin displayed a greatly elevated DNA binding level by 4.8 times upon sono-activation (Fig. 2I), whereas the ultrasound-mediated micro-cavitation effect could scarcely enhance the binding performance of carboplatin. As the released Pt(II) species but not the inert Pt(IV) prodrug can effectively bind DNA,^13^ the data suggest the efficient sono-activation of cyaninplatin and that ligand **1** indeed performed as an ultrasound antenna to facilitate the activation of Pt(IV) prodrugs for sono-sensitization–augmented chemotherapy. Noteworthy, the cyanine dye with a positive charge and a large aromatic structure may have contributed to the high level of Pt-DNA binding via DNA intercalation. After incubation, the broadened absorption peak of cyaninplatin (fig. S10A) and the change in the circular dichroism spectra of ctDNA (fig. S10B) validated the interaction between cyaninplatin and ctDNA.

**Fig. 3.**
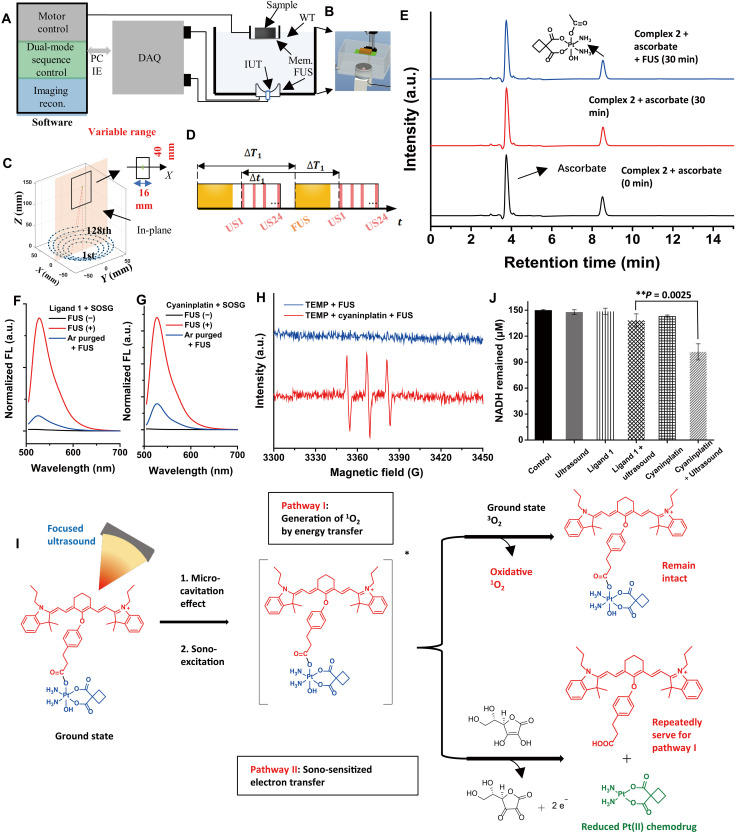
Mechanistic study of focused ultrasound (FUS)–mediated reduction of cyaninplatin. (**A** and **B**) Scheme of device setup of FUS system. (**C** and **D**) Setup of FUS array. DAQ, data acquisition; Mem., membrane; IUT, imaging ultrasound transducer; US, ultrasound; WT, water tank; ∆*T*_1_ is 60 ms; ∆*t*_1_ is 0.5 ms; the duty circle is 67% for FUS unit. (**E**) Complex 2 in phosphate-buffered saline (PBS) solution containing 5 mM ascorbate (pH 7.4) at 37°C, with or without FUS activation (4 W, 30 min). Fluorescent spectra of Singlet Oxygen Sensor Green (SOSG) with FUS-activated (**F**) ligand **1** or (**G**) cyaninplatin (4 W, 15 min). (**H**) Electron paramagnetic resonance (EPR) spectra of tetramethylpiperidine (TEMP) with FUS-activated cyaninplatin (4 W, 15 min). (**I**) Proposed mechanism of reduction of cyaninplatin under FUS-activation. (**J**) Oxidation of nicotinamide adenine dinucleotide (NADH) in PBS solution with various treatments (FUS: 4 W, 15 min, NADH: 150 μM, ligand 1 or cyaninplatin: 15 μM). Mean ± SD, *t* test,**P < 0.01, *n* = 3.

We further explored the sono-activation mechanism of cyaninplatin. Complex **2**, *c,t*-[Pt(CBDCA)(NH_3_)_2_(OAc)(OH)] (fig. S11), a typical carboplatin-based Pt(IV) scaffold without a sonosensitizer motif, was treated with our lab-built FUS (Fig. 3, A to D) in the presence of ascorbate. The difference in the reduction of complex **2** with and without FUS treatment for 30 min was negligible (<2%; Fig. 3E), confirming the essential role of sono-sensitizing ligand **1** as an energy converter for the ultrasound-mediated reduction of cyaninplatin. Sonosensitizers at the excited states can further transfer their energy to ground-state oxygen to generate singlet oxygen (^1^O_2_), which is the well-studied type II sono-sensitization process (*33*). The generation of ^1^O_2_ was tested by using Singlet Oxygen Sensor Green (SOSG) as a fluorescent probe. Both ligand **1** and cyaninplatin generated ^1^O_2_ upon sono-activation and induced the increase of peak intensity at 520 nm. In contrast, the generation of ^1^O_2_ decreased by more than 70% in samples that were degassed and purged with argon gas (Fig. 3, F and G, and fig. S12A). We further verified the generation of ^1^O_2_ rather than other types of ROS by electron paramagnetic resonance (EPR) spectroscopy using 2,2,6,6-tetramethylpiperidine (TEMP) as a spin trapping agent, as a typical 1:1:1 triplet pattern from the adduct (2,2,6,6-tetramethylpiperidin-1-yl)oxyl was observed (Fig. 3H and fig. S12, B to D). These data clearly showed that both ligand **1** and cyaninplatin can be excited upon ultrasonication and that the sono-sensitization followed type II rather than type I sono-dynamic process; in the latter process, the generation of hydroxyl radicals or superoxide anions is accomplished by type I sono-sensitizers via direct electron transfer (*18*).

On the basis of the above results, we proposed a possible mechanism for the sono-activation of cyaninplatin (Fig. 3I). When FUS is applied to an aqueous solution, it causes a micro-cavitation effect that excites cyaninplatin containing a sonosensitizer moiety. The excited Pt(IV) prodrug then interacts with the surrounding substances via two possible pathways. The first pathway follows a conventional type II sonodynamic process, whereby excited cyaninplatin transfers energy to ground-state oxygen to generate ^1^O_2_, as discussed above. The second pathway is distinct from a typical sonodynamic process, as it involves intermolecular electron transfer from an electron donor, sodium ascorbate in this case, to cyaninplatin, which facilitates the reduction of the oxidative Pt(IV) complex. Such a metal complex at the excited state has strengthened redox reactivity, likewise to the well-studied photoactivatable Pt(IV) complexes or metallic oxidative catalysts (*17*, *36*). Hence, differed from previously reported radical-driven sono-reduction of free Pt(IV) cations (*37*), this ultrasound-mediated reduction that requires an electron donor was termed sono-sensitized electron transfer.

Considering that the sono-excitation could augment the oxidation capability of cyaninplatin, the oxidation of reduced form of nicotinamide adenine dinucleotide (NADH) by cyaninplatin was measured. When cyaninplatin was incubated with an excess amount of NADH, upon treatment with ultrasound, the amount of remaining NADH decreased markedly to 67.9%, which was greatly lower than that of either ligand **1** under sono-activation (92.2%) or cyaninplatin only (95.5%; Fig. 3J). The NAD^+^/NADH pool dominantly harnesses the intracellular redox homeostasis by supporting antioxidant defense to scavenge ROS and regenerating cellular reductants, and it also supports DNA repair as a vital substrate via poly(adenosine diphosphate–ribose) polymerase–related pathways (*38*). Consequently, the oxidative disturbance of redox homeostasis by sono-activation of the Pt(IV) prodrug could potentiate the oxidative stress-induced cell damage, thereby augmenting its therapeutic effects by impairing the cellular detoxification and potential DNA repair (*39*, *40*).

### Cellular uptake and subcellular distribution

**Fig. 4.**
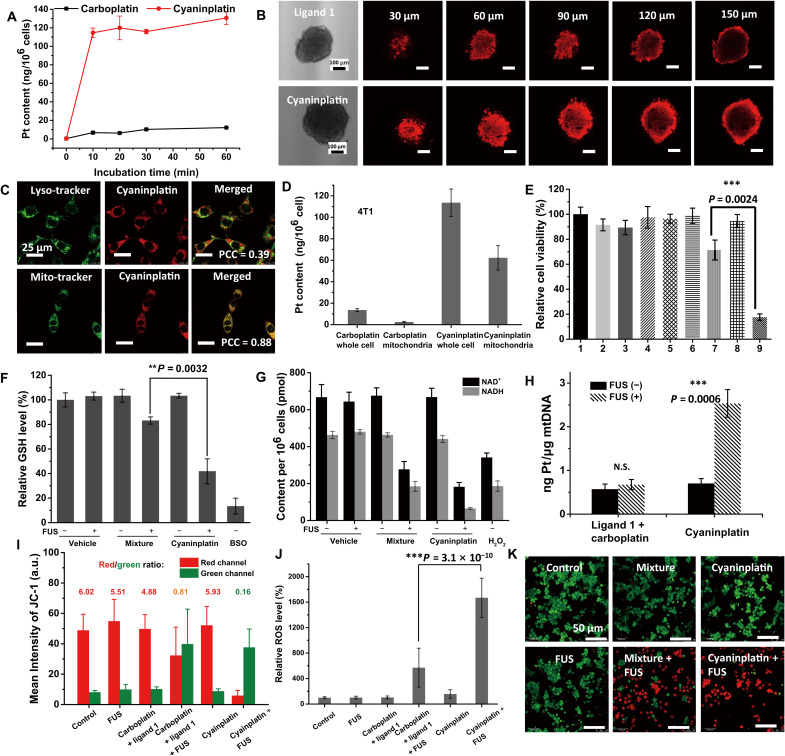
Cell-based in vitro experiments. (**A**) Cellular accumulation of cyaninplatin (12.5 μM) and carboplatin (12.5 μM). (**B**) Uptake and infiltration of cyaninplatin in 4T1 tumor spheroids (25 μM, 30 min). (**C**) Subcellular distribution of cyaninplatin (12.5 μM, 30 min). (**D**) Pt contents in mitochondrial parts of cells (drug feeding: 12.5 μM, 30 min). (**E**) Relative cell viability under different treatment conditions: 1. vehicle control [0.5% dimethylformamide (DMF)], 2. focused ultrasound (FUS)(3.5 W, 15 min), 3. cisplatin (12.5 μM), 4. carboplatin (12.5 μM), 5. carboplatin + FUS, 6. ligand **1** + carboplatin, 7. ligand **1** + carboplatin + FUS, 8. cyaninplatin (12.5 μM), 9. cyaninplatin + FUS. (**F**) Relative glutathione (GSH) level with different treatment (drug feeding: 25 μM, 30 min; FUS: 3.5 W, 10 min). (**G**) Oxidative depletion of nicotinamide adenine dinucleotide (NADH) with different treatment (drug feeding: 25 μM, 30 min; FUS: 3.5 W, 10 min). (**H**) Pt-binding level of mitochondrial DNA (mtDNA) of cells treated with carboplatin + ligand **1** or cyaninplatin (12.5 μM, 30 min) with FUS (3.5 W, 15 min). (**I**) Quantitative result of JC-1 staining (drug feeding: 25 μM, 30 min; FUS: 3.5 W, 10 min). (**J**) DCFH-DA staining for reactive oxygen species (ROS) levels upon various treatments (cyaninplatin at 25 μM, 30 min; FUS: 3.5 W, 10 min). (**K**) Calcein-acetoxymethyl (AM)/PI double staining of cells received different treatments (drug feeding: 25 μM, 30 min; FUS: 3.5 W, 10 min). 4T1 cells were used in all these assays. Mean ± SD, *t* test, **P < 0.01, ***P < 0.001, *n* = 3.

After incubation with cyaninplatin (12.5 μM) for 10 min, the accumulation level of platinum in 4T1 cells reached 114 ng per million cells, while the level for carboplatin was only 12 ng Pt per million cells. The accumulation level of cyaninplatin rapidly increased in the first 10 min of drug feeding and then slightly increased over the following 60 min (Fig. 4A). The improved cellular accumulation of cyaninplatin can be attributable to the lipophilic and cationic features endowed by ligand **1**. Confocal laser scanning microscopy (CLSM) verified the efficient accumulation of cyaninplatin in 4T1 and HeLa cells (fig. S13). In addition, cyaninplatin could infiltrate into the interior region of three-dimensional (3D) tumor spheroids with a size of approximately 200 μm (Fig. 4B). Moreover, we observed the accumulation of cyaninplatin in the mitochondria in both 4T1 and HeLa cell lines, with Pearson’s correlation coefficient of 0.88 and 0.86, respectively (Fig. 4C and figs. S14 and S15), which is attributable to the cationic nature of cyaninplatin. Meanwhile, the subcellular distribution of Pt was also determined by inductively coupled plasma mass spectrometry (ICP-MS). The mitochondrial accumulation ratios, defined as the level of Pt in the mitochondria/the level of Pt in the whole cell × 100%, were 54.9 and 52.3% in 4T1 and HeLa cells, respectively, which were apparently higher than those of carboplatin (18.1 and 20.1% in 4T1 and HeLa cells, respectively; Fig. 4D and fig. S16). As the chemotherapeutic efficacy of carboplatin was hindered by its limited accumulation level in the tumor, the swift cellular entrance and efficient accumulation at the mitochondria region promise the potentially enhanced therapeutic efficacy of cyaninplatin by damaging this most fundamental redox center and energy factory in cancer cells (*41*).

### Cytotoxicity against different cancer cell lines

The cytotoxicity of cyaninplatin upon sono-activation (fig. S17) was evaluated with comparison to carboplatin or an equivalent mixture of carboplatin and ligand **1** as a model for combinatorial chemotherapy and sonodynamic therapy. In all the tested carcinoma cell lines, including HeLa, MCF-7, A2780, A2780cisR, A549, A549cisR, and 4T1, the cytotoxicity of cyaninplatin was minimal without ultrasound treatment [median inhibitory concentration (IC_50_) > 40 μM, the limit of solubility in aqueous solutions], while the sono-cytotoxicity was drastically augmented upon sono-activation, with IC_50_ values in the range of 2.0 to 4.3 μM, which were the lowest among all the treatment groups (table S1 and fig. S18). The sono-sensitization index, defined as the IC_50_ without sono-activation/the IC_50_ with sono-activation, ranged from 6.4 to greater than 20. For the platinum-resistant A2780cisR and A549cisR cells, because of potential detoxification and other resistance mechanisms (*40*, *42*), remarkably compromised cytotoxicity occurred in these cells treated with the mixture, with resistance factors (RFs; the IC_50_ in resistant cells/the IC_50_ in sensitive cells) of 2.3 and 2.9, respectively; intriguingly, cyaninplatin upon sono-activation substantially surmounted this resistance effect with the RFs of as low as 1.0 and 1.2, respectively, leading to a therapeutic approach, i.e., SSCT, beyond the mere combination of chemotherapy and sonodynamic therapy. In addition, at a fixed concentration of 12.5 μM, sono-activated cyaninplatin (group 9, Fig. 4E) exhibited cell killing efficiency superior to that of the mixture (group 7, Fig. 4E). Noteworthy, the parent drug carboplatin (group 4) displayed minimal cytotoxicity under these conditions due to its highly dose-dependent effect and relatively low level of cellular uptake (*43*). Unlike cisplatin, inactivated cyaninplatin exhibited negligible cytotoxicity toward lung fibroblast cell line MRC-5, ensuring its biosafety for further in vivo experiments (fig. S19).

### Cellular redox homeostasis disturbed by cyaninplatin

Oxidative transition metal species are known to be able to perturb intracellular redox homeostasis and further strengthen oxidative stress, which may lead to cell death (*44*–*48*). Analogously, the sono-activated reduction of cyaninplatin, which can efficiently accumulate in the mitochondria, may also perturb the redox balance of cancer cells. To further explore the cytotoxic mechanism of cyaninplatin upon sono-activation, which robustly induced cell death, we first evaluated its impact on two vital cellular reductants that are abundant in the mitochondria: glutathione (GSH) and NADH (*49*). The level of GSH in 4T1 cells treated with FUS-activated cyaninplatin descended by 58.1%, while the level decreased by only 16.8% in cells treated with the mixture (equivalent carboplatin and ligand **1**) activated by FUS (Fig. 4F). Compared with normal levels of NAD^+^ and NADH in 4T1 cells, those in the cells treated with sono-activated cyaninplatin decreased by 73.7 and 86.0%, respectively (Fig. 4G). In the cells treated with sono-activated cyaninplatin but not other samples including the mixture of ligand **1** and carboplatin, the ratio of NAD^+^/NADH markedly increased (fig. S20).

We subsequently studied the level of Pt in mitochondrial DNA (mtDNA) in the treated cells. Upon ultrasound treatment, the Pt level elevated by 3.6-fold in cells treated with cyaninplatin, reaching 2.5 ng Pt/μg DNA (Fig. 4H and fig. S21), while in the cells treated with the mixture of ligand **1** and carboplatin, no substantial elevation in the level of Pt in mtDNA occurred. A slight increase in the Pt level in nuclear DNA (nuDNA), reaching 0.3 ng Pt/μg DNA, was also observed for sono-activated cyaninplatin but not the mixture (fig. S21). These data evidently prove the sono-activation of the Pt(IV) prodrug in cells, as only the released Pt(II) species can efficiently bind DNA (*41*, *50*). We further applied various ROS scavengers to pretreat the cells before treatment by cyaninplatin or ligand **1** (*49*). With pretreatment by NaAsc, *N*-acetyl cysteine (NAC), or sodium azide (NaN_3_), intracellular ROS levels intensively reduced (fig. S22), and the cytotoxicity from sono-activated ligand **1** drastically descended (fig. S23A). Sodium pyruvate (NaPyr) was less effective in ROS scavenging due to its specificity against intracellular H_2_O_2_ rather than ^1^O_2_. In comparison, the viability of NaAsc-pretreated cells (fig. S23B) did not recover significantly after treatment by cyaninplatin, while NAC can potently regenerate intracellular reductive thiol-pool for both ROS scavenging and detoxification of platinum therapeutics (*51*). In addition, an occurrence of cellular vacuolation was observed in cells pretreated with NaAsc and treated with cyaninplatin (fig. S22B). These results demonstrated that Pt-mtDNA binding is vital for antitumor activity, considering that the hypoxia tumor microenvironment and antioxidant defense system may strongly restrain the performance of ^1^O_2_-dependent sonodynamic therapy (SDT).

As the reduction of cyaninplatin disturbed intracellular redox homeostasis, we further evaluated mitochondrial damage caused by sono-activation. As a fluorescent indicator for mitochondrial membrane potential (ΔΨm), JC-1 aggregates (red fluorescence) represent the strong transmembrane potential of healthy mitochondria (red/green ratio = 6.02; Fig. 4I and fig. S24), generated by proton pumps. While after treatment with sono-activated cyaninplatin, the robust depolarization of mitochondria occurred, presented by the increased green fluorescence of JC-1 monomers due to the decreased ΔΨm (Fig. 4I). Notably, although both were activated by ultrasound, the decrease of ΔΨm by cyaninplatin (red/green ratio = 0.16) was more intensive than by the mixture (red/green ratio = 0.81), owing to the strengthened oxidization capability of cyaninplatin under excitation. Both the oxidative and DNA damage to mitochondria can lead to the break of the respiratory chain and cut off the supply of intracellular reductants, whereby the ROS generation is boosted. As indicated by a DCFH-DA staining assay, the relative ROS level in 4T1 cells treated with sono-activated cyaninplatin (1667%) was far beyond the cells treated with sono-activated mixture (570%), showing the superior capability of cyaninplatin to generate ROS (Fig. 4J and fig. S25).

Such robust elevation of Pt-DNA binding and ROS levels in cells may contribute to rapid cell death. To corroborate this hypothesis, calcein-AM/propidium iodide (PI) double staining was carried out immediately after ultrasound treatment to visualize the cell death process. Sono-activated cyaninplatin instantly killed most of the cells, as indicated by the increased fraction of PI-positive staining and decreased fraction of calcein-negative staining (Fig. 4K). Noteworthy, the decrease in cell number after treatment was notable (Fig. 4K and fig. S24), attributable to the robust cell killing efficiency of cyaninplatin. In tumor spheroids, cyaninplatin upon sono-activation exhibited compelling cell killing efficiency and destruction of spheroids, compared to the spheroids treated with the mixture and ultrasound (fig. S26).

These data explicitly demonstrate that the sono-activated reduction of cyaninplatin can impair the antioxidant defense by depleting cellular reductants. Subsequently, the ROS generated from sono-sensitization damage the vital organelle mitochondria to trigger instant cell death and initiate long-lasting chemotherapy by the released carboplatin. Thus, a therapeutic approach of SSCT was accomplished to maximize the merits of both Pt(IV) prodrug-based chemotherapy and sonodynamic therapy.

### SSCT induces ICD

As SSCT caused instant cell death by the burst of ROS, we proceeded to explore the potential of cyaninplatin to trigger ICD under FUS (*52*, *53*). Three main hallmarks of ICD, including the exposure of calreticulin (CRT; fig. S27), the secretion of ATP (fig. S28), and the extra-nuclear release of high-mobility group box 1 (HMGB1; fig. S29), were detected from 4T1 cells receiving treatment of SSCT, with the comparison to the treatment of oxaliplatin as a positive control. The potent ICD induced by cyaninplatin can elicit immune responses through strengthened antigen presentation and evoke immune cells to promote the therapeutic outcome of cyaninplatin in the following in vivo anticancer experiments.

### Cyaninplatin induces paraptosis

**Fig. 5.**
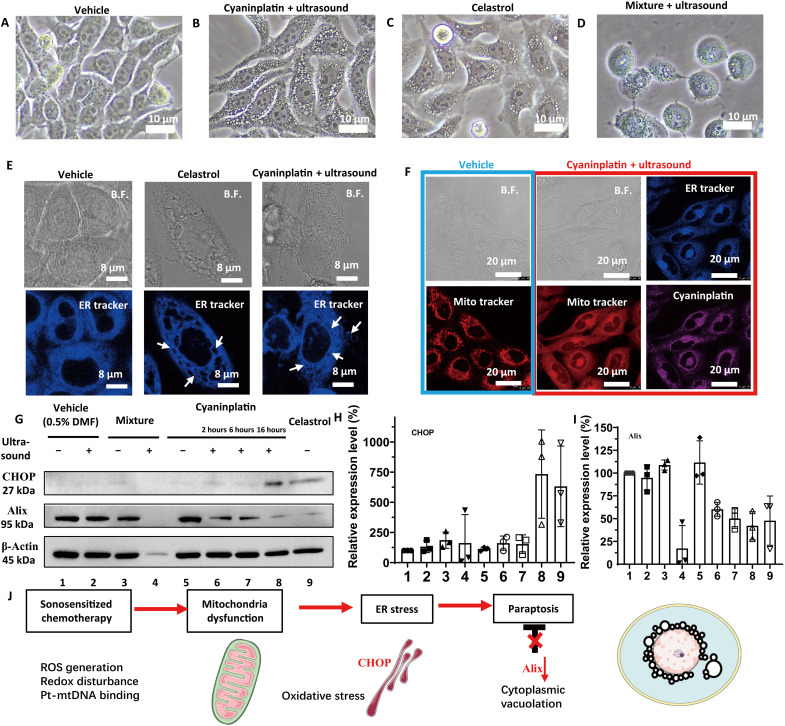
Exploration of cell death mode. Bright-field (B.F.) images of HeLa cells under various treatments. (**A**) Vehicle control (0.5% DMF), (**B**) cyaninplatin (10 μM, 30 min) + focused ultrasound (FUS) (3 MHz, 3.5 W, 15 min), (**C**) celastrol (2 μM, 6 hours), and (**D**) carboplatin + ligand **1** (35 μM, 30 min) + FUS. 5. Carboplatin + FUS, 6. ligand **1** + carboplatin, 7. ligand **1** + carboplatin + FUS. (**E**) Confocal laser scanning microscopy (CLSM) images of cells with cytoplasmic vacuolation and (**F**) depolarization of mitochondria. (**G**) Western blot and (**H** and **I**) quantitative results of HeLa cells with different treatment conditions. Drug feeding: mixture of carboplatin + ligand **1** (35 μM, 30 min); cyaninplatin (10 μM, 30 min); celastrol (2 μM, 6 h). FUS: (3 MHz, 3.5 W, 15 min). (**J**) Proposed mechanism for sono-activated cyaninplatin to induce paraptosis. Mean ± SD, *n* = 3.

To further investigate the cell death mode induced by sono-activated cyaninplatin, the morphological change of HeLa cells was observed first. Intriguingly, different from the typical apoptosis caused by doxorubicin (fig. S30), the typical formation of cytoplasmic vacuolation after treatment by sono-activated cyaninplatin was observed. Cells remained attached to the petri dish, and nucleus structure basically remained intact for a long period till 16 hours after treatment (fig. S31). This occurrence of “bubbles” inside of cytoplasm was identical to the cells treated with celastrol, as the most typical sign of paraptosis due to ER-related damages (Fig. 5, A to C) (*54*). The same phenomena did not occur for cells killed by equivalent mixture of sono-activated ligand **1** and carboplatin, which exhibited more features of necrosis (Fig. 5D). Since paraptosis is highly related to the dysfunction of mitochondria and oxidative ER stress caused by photodynamic therapy, (*55*) we further stained the cyaninplatin-treated cells with mito-tracker and ER tracker. The CLSM images evidently confirmed the swelling of cells and ER-derived vacuoles in cells treated with celastrol or sono-activated cyaninplatin (Fig. 5E). Because of severe mitochondria dysfunction with the decrease of ΔΨm, mito-tracker could no longer stain the cyaninplatin-treated cells; hence, a nonspecific staining occurred compared to untreated cells (Fig. 5F). The Western blot assay validated that sono-activated cyaninplatin induced oxidative ER stress by up-regulation of C/EBP homologous protein (CHOP) to a level of 730% at 16 hours (Fig. 5, G and H, and fig. S32). The expression level of Alix decreased to 49.8% at 2 hours after treatment (Fig. 5, I and J); and the down-regulation of this most important endogenous paraptosis inhibitor eventually led to programmed paraptosis (*56*). As ER stress–related programmed cell death, paraptosis is well-known to induce a stronger ICD effect than necrosis (*51*), which may benefit for further in vivo anticancer efficacy.

### Tissue penetration test

**Fig. 6.**
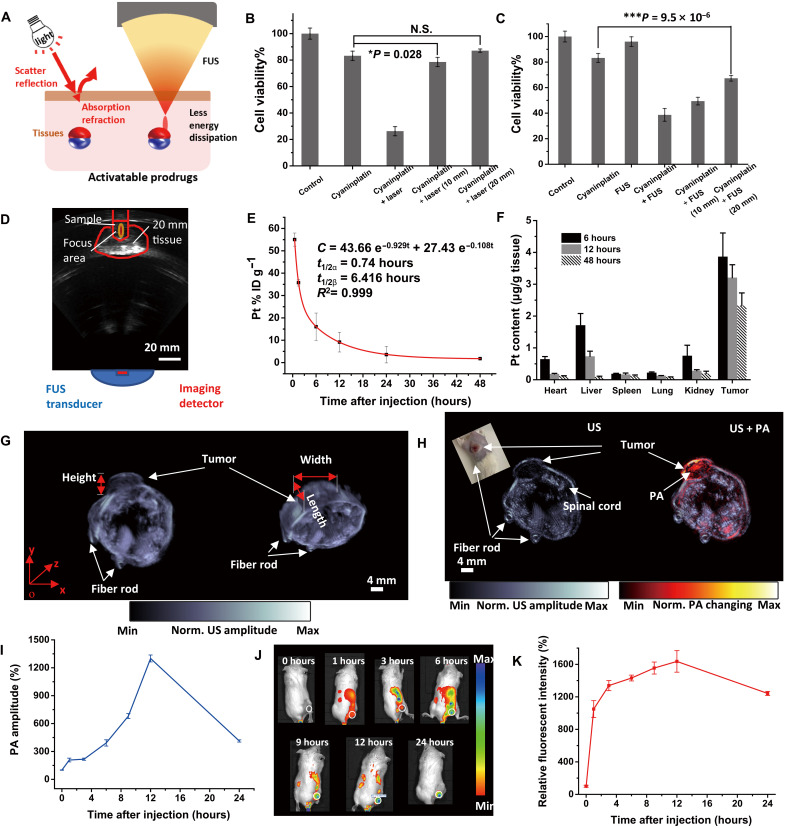
Circulation, biodistribution, and multimodal imaging. (**A**) Schematic illustration of tissue penetration process of light and focused ultrasound (FUS). (**B**) Tissue penetration test of near-infrared (NIR) laser-activated cyaninplatin against chicken breast tissues (HeLa cells, drug feeding: 12.5 μM, 30 min; laser: 808 nm, 0.33 W cm^−2^, cell viability was tested at 24 h post-treatment). (**C**) Tissue penetration test of sono-sensitized chemotherapy (SSCT) efficacy against chicken breast tissues (drug feeding: 12.5 μM, 30 min; FUS: 3.5 W, 15 min, cell viability was tested at 24 hours after treatment) and (**D**) corresponding B-mode ultrasound imaging to monitor the treatment process. (**E**) Blood circulation for mice with intravenous injection of cyaninplatin. (**F**) Biodistribution of cyaninplatin at 6, 12, and 48 hours after injection of cyaninplatin. (**G**) Semi-3D reconstruction of tumor area by high-resolution ultrasound (US) imaging. (**H**) Photoacoustic (PA) computed tomography to verify the accumulation of cyaninplatin in the tumor at 9 hours after injection. (**I**) Quantitative result of photoacoustic signal in the tumor region after injection of cyaninplatin. (**J**) NIR optical imaging to verify tumor accumulation of cyaninplatin and (**K**) corresponding quantitative data. Mean ± SD, *t* test, *P < 0.05, ***P < 0.001, *n* = 3.

As an effective approach to activating anticancer prodrugs, the most fascinating property of ultrasound is its improved tissue penetration depth. Even for light in the NIR-II (1000 to 1700 nm) region, the effective penetration depth is still in the millimeter range, which is insufficient for the noninvasive phototherapy of lesions embedded in deep tissues (*18*). In test tubes, an 808-nm NIR laser (15 min, 0.33 W cm^−2^) could also activate cyaninplatin (fig. S33), and the photoactivated prodrug inhibited cell viability by 74%. However, for HeLa cells under coverage by thick chicken breast tissue (10 or 20 mm; fig. S34), the phototherapy was no longer effective although the power density had already reached the maximum permissible exposure (Fig. 6, A and B). In stark contrast, the percentage of cell viability upon treatment with sono-activated cyaninplatin only slightly increased from 38% for no tissue coverage to 49% for coverage with 10-mm tissue; although the energy dissipation was stronger when ultrasound penetrated 20 mm of tissue, sono-activated cyaninplatin could still inhibit cell viability by 33% beneath this thick layer of tissue (Fig. 6, C and D, and fig. S35). This enhanced tissue penetration by SSCT allowed us to noninvasively ablate deep tumors. Yet, the decay in sono-activation efficiency remained (Fig. 6C), implying that more research is required to establish compensational methods that can meet the clinical demands for FUS-mediated treatment.

### Tumor accumulation of cyaninplatin

The pharmacokinetic behavior of cyaninplatin was determined by ICP-MS analysis of blood collected from mice after intravenous injection. Cyaninplatin followed a typical two-compartmental model, exhibiting a half-life of *t*_1/2α_ = 0.74 hours for the distribution phase and *t*_1/2ß_ = 6.416 hours for the elimination phase (Fig. 6E). The body clearance of cyaninplatin was determined by a biodistribution assay. In tissues harvested at 6, 12, and 48 hours after injection, we observed high Pt accumulation levels in the tumor and also that the Pt content in the liver and kidney gradually decreased with time, elucidating the completion of excretion within 48 hours (Fig. 6F).

Multimodal imaging for cyaninplatin-injected tumor-bearing mice was further conducted. The tumor geometry was first visualized by high-resolution ultrasound imaging. With 20 scanning sections of transverse planes, the tumor can be reconstructed in a semi-3D fashion to provide guidance for the accurate application of FUS at the tumor site (Fig. 6G). Moreover, NIR (excitation at 780 nm) photoacoustic computed tomography and fluorescence imaging analyses were performed to determine the accumulation time. Both the photoacoustic imaging (Fig. 6, H and I, and fig. S36) and fluorescent signal (Fig. 6, J and K) indicated that the accumulation of cyaninplatin in the tumor reached the maximum at 12 hours after injection, and the level gradually decreased in the following periods. Combined with the guidance of ultrasound/photoacoustic/optical imaging modalities, a spatiotemporally controlled theranostic approach could be achieved for accurate tumor treatment with minimized adverse effects.

### In vivo anticancer activity of cyaninplatin

**Fig. 7.**
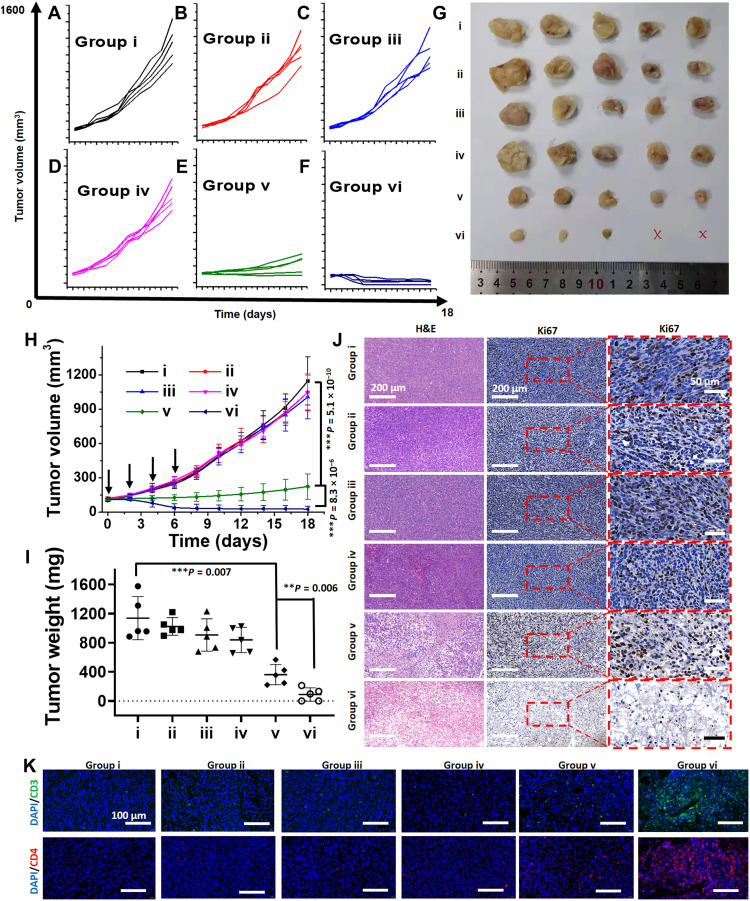
In vivo antitumor evaluation of cyaninplatin. Tumor growth curve of individual mice from groups i to vi: (**A**) vehicle control (group i); (**B**) carboplatin + ligand 1 (equivalent mixture, dose at 3 mg Pt kg^−1^, group ii); (**C**) cyaninplatin (3 mg Pt kg^−1^, group iii); (**D**) focused ultrasound (FUS) at 12 hours after injection (3.5 W, 20 min, group iv); (**E**) carboplatin (3 mg Pt kg^−1^) + ligand 1 + FUS (group v); (**F**) cyaninplatin (3 mg Pt kg^−1^) + FUS (group vi). (**G**) Photos of dissected tumors after treatment. (**H**) Mean tumor volume of mice in different groups of treatment. (**I**) Mean tumor weight at day 18 after treatment. (**J**) Hematoxylin and eosin (H&E) staining and Ki67 staining for dissected tumors in different groups. (**K**) CD3^+^ and CD4^+^ staining for tumors from each group. Mean ± SD, *t* test, **P < 0.01, ***P < 0.001, *n* = 5. DAPI, 4′,6-diamidino-2-phenylindole.

As monitored by multimodal imaging, 12-hour postinjection was the optimal therapeutic time window for sono-activation. A syngeneic model based on the 4T1 (murine mammary carcinoma) cell line that resembles stage IV breast cancer was established in immunocompetent BALB/c mice (*57*). To properly treat the tumors with FUS, programmable scanning was used to move the focus of ultrasonication to three positions to cover the whole region of tumors (figs. S37 and S38). In addition, the temperature of tumors was monitored to avoid local hyperthermia (temperature elevation < 3°C) and prevent damage to adjacent tissues. Treatment with the inactivated mixture, inactivated cyaninplatin, or FUS only (groups ii to iv) did not result in any inhibition of tumor growth as compared to the control group (group i), which was injected with the vehicle solution alone (Fig. 7, A to D, and fig. S37B). In mice treated with the mixture of carboplatin and ligand **1** (group v), after the fourth treatment on day 6 and upon ultrasound activation, three tumors continued growing as a sign of recurrence (Fig. 7E and fig. S39). In contrast, the growth of tumors in mice treated with sono-activated cyaninplatin was drastically inhibited (group vi), with two tumors being completely eradicated after treatment (Fig. 7, F and G, and fig. S40). The relative mean relative tumor volume from the treatment with sono-activated cyaninplatin was only 24.3% on day 18, compared with 200% from the treatment with the activated mixture (Fig. 7H). The average tumor weight of mice treated with sono-activated cyaninplatin was as low as 7.4% (Fig. 7I).

From the histological analysis of tumor slides, the sono-activated cyaninplatin not only directly destroyed tumor tissue but also decreased the level of tumor proliferation factor Ki67 to prevent potential recurrence of treated tumors (Fig. 7J). The relative expression level of Ki67 in the tumor treated with FUS-activated cyaninplatin drastically declined to 24% of that in the untreated group, where the level in the tumor treated with carboplatin and ligand **1** remained at 49% (fig. S41). Furthermore, the immunofluorescence staining results elucidated the successful T cell (CD3-positive) infiltration and the evoking of T helper cells (CD4-positive) in the tumor of mice treated with sono-activated cyaninplatin (Fig. 7K). While for the mixture of carboplatin and ligand **1** upon sono-activation, the infiltration of T cells and activated T helper cells was much lower in the tumor (Fig. 7K). During the whole treatment period, no notable loss of body weight and abnormal behaviors, such as anorexia or depression, occurred for all the treatment groups (fig. S42). Blood chemistry and hematoxylin and eosin (H&E) staining results suggested that no systemic toxicity was caused to the main organ tissues after treatment with sono-activated cyaninplatin (figs. S43 and S44).

The above-described robust therapeutic outcomes of SSCT can be attributed to several advantages of rational design: (i) the molecular functionalization of a Pt(IV) motif with a sonosensitizer allowed for the colocalized accumulation of these two therapeutic moieties and the simultaneous sono-activation; (ii) beyond conventional sono-sensitization process, the activation of cyaninplatin not only rapidly depleted cellular reductants, which impeded ROS-related damage, but also boosted the release of the Pt(II) chemotherapeutics, which continued exerting anticancer activity after the cessation of sono-activation and lastly induced paraptosis with stronger ICD effect; and (iii) the utilization of FUS with multimodal imaging guidance enabled the achievement of accurate and spatiotemporally controlled tumor-specific treatment with minimal side effects and the higher tissue penetration than photo-activation. In addition, ultrasound may also physically facilitate the diffusion of the drug into the interior of solid tumors and alleviate tumor hypoxia to enhance sono-sensitization (*21*, *28*–*30*).

### Tissue penetration ability of FUS ex vivo and in vivo

**Fig. 8.**
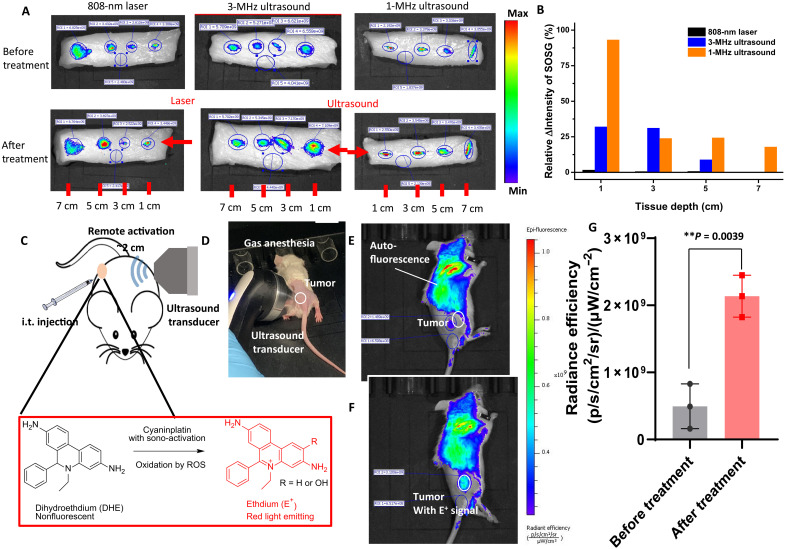
Tissue penetration capability of focused ultrasound (FUS) to excite cyaninplatin. (**A**) Fluorescent images of chicken breast tissue with injection of mixture of cyaninplatin (15 μM) and Singlet Oxygen Sensor Green (SOSG)(20 μM) and then treated with different excitation sources: 808 nm laser (0.33 W cm^−2^, 10 min), FUS (1 or 3 MHz, 3.5 W, 10 min). Ex: 480 nm/Em: 530 nm. (**B**) Quantitative result of normalized increase of SOSG signal (ΔIntensity_SOSG_ = Intensity_after_ − Intensity_before_) after treatment. The ΔIntensity_SOSG_ in each group without tissue coverage was defined as 100%. (**C**) Schematic illustration and (**D**) device setup of remote activation of cyaninplatin in the 4T1 tumor xenograft of mice. Fluorescent images of mice with intratumoral (i.t.) injection (**E**) before and (**F**) after FUS treatment (1 MHz, 3.5 W, 10 min). (**G**) Quantitative result of fluorescent signal in the tumor region. Ex: 520 nm/Em: 620 nm. Mean ± SD, *n* = 3, *t* test, **P < 0.01.

The activation of cyaninplatin by ultrasound can be verified by the detection of ROS using SOSG, as the molecular excitation of cyaninplatin is accompanied with ROS generation (Fig. 3I). To simulate deep lesions in clinical scenario, chicken tissue was injected with a mixture of cyaninplatin and singlet oxygen sensor green (SOSG) at the depths of 1, 3, 5, and 7 cm. The tissue was irradiated with 808-nm laser or FUS. The sample in the test tube without tissue coverage could be activated by all these three excitation sources (fig. S45). After penetrating 1 cm of chicken tissue, the normalized increase of SOSG signal (ΔIntensity_SOSG_) by 808-nm laser activation drastically descended to 1.68%, while the ΔIntensity_SOSG_ for 3 and 1 MHz FUS remained as high as 32.1 and 93.2%, respectively (Fig. 8, A and B). As therapeutic ultrasound with lower frequency tends to have higher tissue-penetrating capability (*21*), the ΔIntensity_SOSG_ of 1-MHz FUS remained at 17.9% after penetrating 7 cm of tissue which was too deep for 3-MHz FUS to reach, and the laser was not effective at all at this depth (Fig. 8B). Subsequently, we conducted the evaluation of tissue penetrating effect of FUS in vivo on 4T1 tumor–bearing mice. The intratumorally injected cyaninplatin could be effectively excited by ultrasound across the mice body (~2 cm) to generate ROS (Fig. 8, C to G). These results validated that FUS can effectively excite cyaninplatin with a centimeter range of tissue depth in both ex vivo and in vivo, which has great potential for clinical translations.

## DISCUSSION

In summary, toward the challenge of activating platinum anticancer prodrugs by mechanical force that has improved tissue penetration to realize specific bond scission, we developed cyaninplatin, a Pt(IV) prodrug that can be controllably activated by ultrasound. Such a molecular design paradigm achieved superior concomitant therapeutic outcomes in a carrier-free manner, as cyaninplatin was well internalized by cancer cells and accumulated specifically in the mitochondria. Upon irradiation with ultrasound, the prodrug was reduced to chemotherapeutic carboplatin via a sono-sensitized electron transfer process. Simultaneously, sono-activated cyaninplatin generated ROS and depleted intracellular reductants, thereby enhancing ROS-mediated mitochondrial damage and cell killing efficiency. Unlike combinatorial chemo/sonodynamic therapy with carboplatin and ligand **1**, sono-activated cyaninplatin overcame platinum resistance and potently induced ICD from the paraptotic cell death caused by sono-activated cyaninplatin. Markedly, this SSCT was still effective in the centimeter range beneath the tissue, demonstrating the realization of enhanced tissue penetration by FUS. With the guidance of high-resolution ultrasound imaging, accurate application of FUS at the tumor site was achieved, and optical imaging along with photoacoustic imaging allowed the spatiotemporally controlled activation of cyaninplatin in the tumor. The outstanding antitumor behavior implemented by sono-activated cyaninplatin was also augmented by its ability to evoke immune responses via the recruitment and activation of T cells. Overall, we present a small-molecule anticancer prodrug that can be effectively activated by mechanical force in a spatiotemporally controlled manner and that can achieve SSCT, which represents an ultrasound-mediated theranostic modality for cancer. As a small-molecule prodrug with multiple functionalities, the prodrug strategy presented in this work is affordable to be incorporated with various other therapeutic strategies in further potential preclinical studies. This research also improves the understanding of sono-activation process of small molecules and broadens the application of biomedical ultrasound and sono-activatable prodrugs.

## MATERIALS AND METHODS

### Materials and instruments

Detailed information about materials and instruments is contained in the Supplementary Materials. All chemicals, reagents, and antibodies are used without modification unless specified.

#### FUS system

The imaging-guided programmable FUS system used in this study was lab-customized. As depicted in Fig. 2 (A to D), the integrated US/FUS platform includes three main parts: a dedicated software including control unit and image reconstruction, a data acquisition (DAQ) system, and a therapy/imaging probe. The control unit has a motion controller to translate the sample, a sequence controller to synchronize the FUS transmission, and the US imaging. The DAQ (Vantage, Verasonics Inc., USA) has 256 Tx/Rcv channels in which 128 channels are used for FUS therapy and the remaining ones are used for US imaging. The therapy/imaging probe integrates a 128-element FUS transducer with an Archimedean spiral form arrangement and a linear-array imaging transducer. Archimedhe FUS transducer (H-302, Sonic Concepts Inc., Seattle, WA) has a 2-MHz center frequency, ~67% bandwidth, and 150-mm geometrical focus length. The linear imaging transducer (IP-104, Verasonics Inc., USA) has a 3.5-MHz center frequency and ~89% bandwidth and can fit in a central opening in the FUS transducer. The FUS transducer and the imaging transducer were aligned in the azimuthal direction via the marks on the transducer surfaces. An image calibration approach was used to align in the axial direction. The elliptical focus region of the array-based FUS transducer can be dynamically steered by phase programming in both the axial (40 mm, 3 dB) and the lateral directions (16 mm, 3 dB). The transmitting power can also be programmed throughout the treatment cycle. For US imaging, a diverging wave transmission with 24-times transmission in 1 cycle is used to achieve a video-rate imaging speed (17 frames/s).

### Stability and sono-sensitized reduction of cyaninplatin

To test the stability in PBS, cyaninplatin (10 μM) was dissolved in PBS buffer [(pH 7.4) 1% *N*,*N*′-dimethylformamide (DMF)] and incubated at 310 K in the dark. At 24 hours, the sample was analyzed by high-performance liquid chromatography (HPLC) to determine the percentage of Pt(IV) remained. To test the stability in complete RPMI 1640 culture medium [containing 10% FBS, 2 mM l-glutamine, and penicillin/streptomycin (100 IU mL^−1^)] or cell lysate (from A2780 cells by 1% Triton X-100 lysis, protein concentration adjusted to 1.5 mg ml^−1^), cyaninplatin was dissolved in these two solutions (with 1% DMF). At determined time intervals, 50 μl of samples was taken out and diluted with 200 μl of methanol with subsequent centrifugation at 14,000*g* to remove proteins, and then the supernatant was analyzed by HPLC. Also, a portion of cyaninplatin in fresh lysates was treated with FUS (3.5 W, 15 min) and analyzed by the same method.

Since the absorbance of ligand **1** and cyaninplatin is identical at 700 nm, the peak area (by UV-vis detector at 700 nm) can indicate the content of these two compounds.

Percentage of remained Pt(IV) was defined as: Pt(IV)% = (Area_Pt_)/(Area_Pt_ + Area_ligand_) × 100%, where Area_ligand_ represents the peak area of ligand **1**, and Area_Pt_ is the peak area of Pt(IV) complex.

To test reduction with or without ultrasound activation, cyaninplatin was dissolved in PBS containing 1% DMF and 5 mM sodium ascorbate, and then the sample (500 μl in an Eppendorf tube) was continuously treated with FUS (1.75 MHz, 4 W, and under this FUS condition, the temperature of samples was quickly elevated to 37°C and remained during the whole treatment process) for totally 60 min. At 15, 30, 45 and 60 min, samples were analyzed by HPLC. For 45-min–treated sample, the reduction product was identified by HPLC-MS. Solvent A: H_2_O with 5% acetonitrile and 0.1% formic acid; solvent B: acetonitrile with 5% H_2_O and 0.1% formic acid; flow rate: 1.0 ml min^−1^. The test samples were eluted following the program: 0% B (0 min) → 25% B (5 min) → 80% B (8 min) → 100% B (16 min) → 0% B (20 min). Complex **2** was tested under the same ultrasound treatment condition as a control to investigate the response of this sonosensitizer-free platinum(IV) complex to ultrasound stimulus. In addition, cyaninplatin in PBS (without ascorbate) was treated with ultrasound for 60 min to verify the electron donor–dependent activation behavior of cyaninplatin.

To test characteristic ^1^H NMR peak for carboplatin released after sono-activation, 1 mM cyaninplatin was dissolved in a mixed solvent of 20% DMF-d7 and 80% D_2_O (with 50 mM ascorbate and 50 mM PBS). After ultrasound treatment (4 W) for 30 min, the ^1^H NMR test was conducted, with reference to standard carboplatin in mixed solvent (20% DMF-d7 and 80% D_2_O).

### ct-DNA binding assay

Carboplatin or cyaninplatin (10 μM) was dissolved in 200 μl of PBS buffer [(pH 7.4) 0.5% DMF] which contained 150 μg of calf thymus (ct-DNA) disodium salt and 5 mM sodium ascorbate. Subsequently, the samples were treated with or without FUS (4 W, 60 min) and then incubated at 37°C in the dark for 24 hours. To precipitate the ct-DNA, 20 μl of sodium acetate (3 M in deionized water) and 400 μl of cold ethanol were added and stored at −80°C for 2 hours. After centrifugation at 20,000*g* for 20 min, the collected DNA was washed three times with 70% ethanol and redissolved in 200 μl of tris-EDTA buffer [10 mM tris and 1 mM EDTA (pH 8.0)]. The amount of DNA and Pt was determined by a NanoDrop spectrometer and ICP-MS, respectively.

### Sono-sensitization of cyaninplatin

To prove that cyaninplatin can be excited by FUS, cyaninplatin or ligand **1** (25 μM, 1% DMF) in PBS (pH 7.4, with or without 30 min of argon gas purging before treatment) was treated with ultrasound (4 W, 30 min). SOSG (20 μM; Thermo Fisher Scientific) as the fluorescent probe was adopted to detect ^1^O_2_ generated during sono-sensitization process by a spectrofluorometer.

For further validation of the generated ROS, 5,5-dimethylpyrroline *N*-oxide (90 mM) or TEMP (20 mM) was used as spin traps for radical products (superoxide anion, hydroxyl radical, etc.) or ^1^O_2_, respectively. The treatment condition was the same as above, and the sample solution was analyzed by EPR immediately after treatment.

### NADH oxidation test

Ligand **1** or cyaninplatin (15 μM, 0.5% DMF) was dissolved in PBS buffer (pH 7.4) containing 150 μM fresh NADH. Samples were treated with or without FUS (4 W, 30 min). Afterward, the amount of remained NADH was determined using an NADH/NAD^+^ Detection Kit (Beyotime-S0175). In a typical procedure, 20 μl of sample from each group was incubated with 90 μl of alcohol dehydrogenase buffer at 37°C for 10 min in a 96-well plate. Meanwhile, a portion of the same sample was purged with nitrogen and preheated at 60°C for 30 min to allow the degradation of NAD^+^, before being added to alcohol dehydrogenase buffer. Then, 10 μl of WST-8 solution (1 mg ml^−1^) was added to these wells and then incubated at 37°C for 30 min. Absorption of formazan product at 450 nm was detected and recorded as the total level of NADH/NAD^+^ from original samples or NADH only from preheated samples.

### Cell culture

A549 (CCL-185), MCF-7 (HTB-22), 4T1 (CRL-2539), HeLa (CCL-2), and MRC-5 (CCL-171) cells were purchased from American Type Culture Collection (ATCC). A2780 and A2780cisR cells (originally purchased from ATCC, HTB-174) were provided by W. H. Ang (Department of Chemistry, National University of Singapore). A549cisR cells were cultured as previously reported (*16*, *17*, *58*). Human breast carcinoma MCF-7 cells, human cervix carcinoma HeLa cells, human lung carcinoma A549 cells, and cisplatin-resistant A549cisR cells were cultured in Dulbecco’s modified Eagle’s medium (DMEM) containing 10% FBS and penicillin/streptomycin (100 IU ml^−1^). Human ovarian carcinoma A2780 and A2780cisR cells were cultured in RPMI 1640 containing 10% FBS, 2 mM l-glutamine, and penicillin/streptomycin (100 IU ml^−1^). Human lung fibroblast MRC-5 cells were cultured in MEM containing 10% FBS, 1% non-essential amino acids (NEAA), 1% l-glutamine, 1% NaPyr, and penicillin/streptomycin (100 IU ml^−1^). In every two passages of A2780cisR and A549cisR cells, 5 μM cisplatin was added to maintain the platinum resistance. All the cells were cultured in humidified incubators at 37°C with 5% CO_2_. Cell counting was performed with a hemocytometer.

### Cellular uptake

HeLa or 4T1 cells (1 × 10^4^ per dish) were incubated in confocal dishes for 24 hours before use. Cells were incubated with 12.5 μM cyaninplatin (0.5% DMF) for 0.5, 2.0, or 8.0 hours, respectively. At these time points, the cells were washed with PBS and observed with a confocal microscope (excitation: 633 nm, emission: 760 nm), and the intensity was quantified for the mean value of 100 single cells using ImageJ 1.53c.

For short-term time-dependent cellular uptake, 4T1 cells were preseeded in six-well plates (1 × 10^5^ per well) for 48 hours. Then, the cells were treated with cyaninplatin or carboplatin (12.5 μM, 0.5% DMF) for 10, 20, 30, or 60 min. At the corresponding time points, the cells were washed with PBS three times and harvested with trypsin. The obtained cells were counted and digested with aqua regia at 75°C, and Pt concentration was determined with ICP-MS. The experiment was conducted in biological triplicates.

### Subcellular distribution

HeLa or 4T1 cells (1 × 10^4^ per dish) were incubated in confocal dishes 24 hours before use. Cells were then incubated with 12.5 μM cyaninplatin (0.5% DMF) for 30 min. After washing with PBS twice, the cells were further stained with 20 μM Hoechst 33342, lyso-tracker green (Thermo Fisher Scientific), ER tracker blue (Thermo Fisher Scientific), or mito-tracker green (Thermo Fisher Scientific) for 20 min at 37°C. The cells were observed with a confocal microscope, and the Pearson’s correlation coefficient was calculated from 50 single cells using ImageJ 1.53c (Coloc 2 Plugin).

For ICP analysis, 4T1 or HeLa cells were preseeded in six-well plates (5 × 10^4^ per well) and incubated for 48 hours. Then, the cells were treated with cyaninplatin or carboplatin (12.5 μM, 0.5% DMF) for 30 min. Then, the cells were washed with PBS three times and harvested with trypsin. Mitochondria of obtained cells were extracted with the Mitochondria Isolation Kit for Cultured Cells (Thermo Fisher Scientific, kit no. 89874) through selective lysis of cell membrane and gradient centrifugation to separate mitochondrial with nonmitochondrial parts of cells, and both parts were collected. The Pt contents in the mitochondrial part and corresponding nonmitochondrial part were determined with ICP-MS after digestion by aqua regia, the Pt content in “whole cell” = Pt content in the mitochondrial part + Pt content in the nonmitochondrial part.

### FUS conditioning

For adherent cells, HeLa cells preseeded in confocal dishes were treated with 12.5 μM complex for 30 min. The cells were subsequently treated with FUS (2 W, 10 min) and stained with calcein-acetoxymethyl (AM) and PI to visualize cell viability instantly (fig. S16).

### Cell viability assay

Cell viability was tested via 3-(4,5-dimethylthiazol-2-yl)-2,5-diphenyltetrazolium bromide (MTT) assay. The cells were first preseeded in six-well plates until the confluency reached 80% after 48 hours. Then, the cells were treated with drug-containing culture medium (0.5% DMF with various concentrations of 0, 2.5, 5, 12.5, 25, or 40 μM) for 30 min. After washing with PBS three times, the cells were trypsinized and resuspended in 2-ml Eppendorf tubes with 1 ml of fresh culture medium with subsequent treatment with FUS (3.5 W, 15 min). Afterward, cells were seeded into 96-well plates (2 × 10^4^ per well), and MTT assay was conducted after 24 hours. The experiments were performed in biological triplicates, and 100% cell viability was defined as the viability of cells treated with 0.5% DMF (30 min) as the vehicle control.

### Tissue penetration test

HeLa cells were first preseeded in six-well plates and treated with cyaninplatin-containing culture medium (0.5% DMF, 12.5 μM) for 30 min. After washing with PBS three times, cells were trypsinized and resuspended in a 2-ml Eppendorf tube (for ultrasound treatment) or a 2-ml glass vial (for laser irradiation). Then, cells were treated with ultrasound (3.5 W, 15 min) or laser [808 nm, 0.33 W cm^−2^, 15 min, at maximum permissible exposure limit (*59*)] through 0-, 10-, or 20-mm-thick chicken breast tissues. After treatment, cells were seeded into 96-well plates to evaluate the tissue penetration ability of laser and ultrasound.

### Depletion of cellular reductants

To test the intracellular GSH levels, 4T1 cells were incubated in six-well plates and treated with 25 μM mixture of carboplatin and ligand **1** or cyaninplatin for 30 min. Then, followed by the same procedure as the cell viability assay, the cells were treated with or without FUS (3.5 W, 10 min). As a positive control, 500 μM buthionine sulfoximine treatment for 24 hours was adopted to completely deplete GSH. At 30 min posttreatment, the cells were collected and washed three times with cold PBS and lysed after counting with 200 μl of 1% Triton X-100 on an ice bath. The GSH level was tested with 5 mM 5,5′-dithio-bis-(2-nitrobenzoic acid) using a microplate reader at 415 nm. The GSH level of the untreated control group was defined as 100%.

To test the NADH concentrations in cyaninplatin-treated cells, 4T1 cells were seeded in six-well plates and then treated with 25 μM mixture of carboplatin and ligand **1** or cyaninplatin for 30 min and treated with FUS (3.5 W, 10 min). At 30 min after treatment, the cells were washed, counted, and then lysed (by 1% Triton X-100). The cellular NAD^+^/NADH levels were tested using an NADH/NAD^+^ Detection Kit (Beyotime-S0175). In a typical procedure, 20 μl of lysate was incubated with 90 μl of alcohol dehydrogenase buffer at 37°C for 10 min in 96-well plates. Then, 10 μl of WST-8 solution (1 mg ml^−1^) was added to the wells and then incubated at 37°C for 30 min. Absorption of formazan product at 450 nm was detected to indicate the total concentration of NADH/NAD^+^, which was referred to a calibration curve prepared using fresh NADH solution. The NADH/NAD^+^ level of the untreated control group was defined as 100%.

To test the NADH level, 50 μl of lysate was heated in a water bath (60°C for 30 min), and under this condition, NAD^+^ was degraded. Last, 20 μl of sample was tested following the same method as mentioned above. In addition, 100 μM H_2_O_2_ for 24 hours was used as the positive control for NADH quenching. These tests were conducted in biological triplicates.

### Nuclear/mtDNA binding test

4T1 cells were cultured in T-75 bottles until confluency reached 80% (~5 × 10^6^ cells). Then, the cells were treated with the mixture of carboplatin and ligand **1** or cyaninplatin only (12.5 μM, 0.5% DMF, for 30 min). After drug feeding, cells were washed three times with cold PBS, trypsinized, and transferred into 2-ml tubes. The suspension of cells was treated with or without FUS (3.5 W, 15 min) and transferred into six-well plates, with further incubation in the dark for 4 hours, at 37°C. The Mitochondria Isolation Kit for Cultured Cells (Thermo Fisher Scientific, no. 89874) was used to obtain mitochondria by centrifugation at 12,000*g*, and the nucleus was collected by centrifugation at 600*g*. The GeneJET Genomic DNA Purification Kit (K0722) was subsequently applied to the fragments to obtain nuDNA and mtDNA. The content of Pt and DNA were determined with ICP-MS after digestion and NanoDrop, respectively.

### ROS quenching in live cells

To further study the contribution from direct ROS damage by ligand **1** and mtDNA damage by Pt-DNA binding on cytotoxicity, ROS scavengers, including sodium azide (NaN_3_, 500 μM, 2 hours), NaPyr (10 mM, 12 hours), NAC (2 mM, 2 hours), and sodium ascorbate (NaAsc, 200 μM, 12 hours), were used to pretreat 4T1 cells. After pretreatment, cells cultured in confocal dishes (around 10,000 cells per dish) were fed with ligand **1** (35 μM) or cyaninplatin (10 μM) for 30 min. Then, the cells were stained with DCFH-DA (20 μM, 30 min), exposed to FUS (3 MHz, 3.5 W, 15 min), and directly observed by CLSM [488 nm excitation (ex.)/525 nm emission (em.)]. Meanwhile, cells at a density of 4000 per well were preincubated in 96-well plates for 48 hours. Then, the cells were treated under the same condition, and MTT assay was conducted at 24 hours after treatment.

### Calcein-AM/PI double staining

Following the abovementioned method to test cell viability, the cells treated with/without cyaninplatin or a mixture of carboplatin and ligand **1** (25 μM, 30 min) and with/without FUS (3.5 W, 10 min) were then stained with calcein-AM (50 μg ml^−1^) and PI (10 μg ml^−1^) for 10 min at 37°C. Twenty microliters of cell suspension were sealed with a glass coverslip for confocal microscopy. Calcein: 488 nm ex./540 nm em. PI: 488 nm ex./640 nm em.

### DCFH-DA staining

4T1 cells were incubated in six-well plates and stained with 50 μM DCFH-DA (37°C, 30 min). Then, following the abovementioned method to test cell viability, the cells were treated with/without cyaninplatin or a mixture of carboplatin and ligand **1** (25 μM, 30 min) and with/without FUS (3.5 W, 10 min). At 3 hours after treatment, 20 μl of cell suspension was sealed with a glass coverslip for confocal microscopy. DCF: 488 nm ex./525 nm em. The ROS level of the untreated control group was defined as 100%.

### Mitochondria membrane potential (ΔΨm) test by JC-1 stain

Following the abovementioned procedure for the cell viability test, the cells treated with/without cyaninplatin or a mixture of carboplatin and ligand **1** (25 μM, 30 min) and with/without FUS (3.5 W, 5 min) were then stained with JC-1 (100 ng ml^−1^) for 10 min at 37°C. Twenty microliters of cell suspension were sealed with a glass coverslip for confocal microscopy. J-monomer: 488 nm ex./540 nm em. J-aggregate: 488 nm ex./ 650 nm em. The ratio of red/green signal intensity from randomly selected 50 cells was calculated to present relative ΔΨm by ImageJ software.

### ICD assay

4T1 cells were treated following the procedure for the cytotoxicity test. The concentration of cyaninplatin or a mixture of carboplatin and ligand **1** was fixed at 25 μM for 30 min, and the ultrasound treatment condition was 3.5 W for 10 min. After treatment, cells were collected by centrifugation at 800*g* and waiting for further use. Meanwhile, treatment with 300 μM oxaliplatin for 6 hours was adopted as a positive control to trigger ICD response.

For CRT exposure, collected cells were stained with primary antibody for 1 hour at room temperature and then incubated with fluorescein isothiocyanate (FITC)–conjugated secondary antibody for another 1 hour. Eventually, cells were stained by Hoechst 33342 (50 ng ml^−1^, 10 min at room temperature) and observed by a confocal microscope.

For HMGB1 release, collected cells were fixed with 4% paraformaldehyde for 12 hours, and then permeabilized by Triton X-100 (0.1% in water) for 10 min. After washing by PBS, HMGB1 primary antibody was applied to stain the cells for 1 hour at room temperature, and then cells were stained with FITC-conjugated secondary antibody (1 hour). Cells were washed with PBS and stained with PI (100 ng ml^−1^) for 30 min and observed with a confocal microscope.

For ATP secretion, 0.5 ml of the supernatant was taken out after centrifugation, and the relative ATP level in the solution was determined by an ATP Determination Kit (Thermo Fisher Scientific, A22066).

### 4T1 tumor spheroids

4T1 cells (less than 1 × 10^4^ cells per well) were cultured in ultra-low attachment six-well plates with continuous shaking during the following 12 hours. Fresh medium was replaced every 3 days for spheroid formation. After 5 days, tumor spheroids with a diameter of around 200 μm were moved into ultra-low attachment 96-well plates (15 spheroids per well). The spheroids were treated with 25 μM ligand or cyaninplatin for 30 min and then observed with a confocal microscope.

The damage caused by cyaninplatin was evaluated via calcein-AM/PI double staining. After drug feeding (25 μM, 30 min), the tumor spheroids were exposed to ultrasound (3.5 W) for 15 min. The tumor spheroids were cultured for another 2 hours and stained with calcein AM (100 μM) and PI (20 nM) for 30 min; the live/dead cells were observed with confocal microscopy.

### Identification of cell death mode

To better observe the morphological change after treatment, the commercially available and portable FUS device (Chattanooga CHA-15-1201 Mobile 2) was used for the following experiments. HeLa cells were preseeded in six-well plates until confluency reached 80%. Then, the cells were treated with 1. vehicle (0.5% DMF), 2. vehicle + FUS (3 MHz, 15 min, 3.5 W), 3. a mixture of carboplatin + ligand 1 (35 μM, 30 min), 4. mixture + FUS (the cells were harvested at 16 hours after treatment), 5. cyaninplatin (10 μM, 30 min), 6. cyaninplatin + FUS (cells were collected at 2, 6, or 16 hours after treatment, as groups 6, 7, and 8), 9. celastrol (2 μM, 6 hours). After treatment, the morphology of cells was observed and recorded, then the cells were lysed, and proteins were collected and quantified by bicinchoninic acid assay (BCA) and mixed with a loading buffer [Laemmli buffer recipe, 4% sodium dodecyl sulfate (SDS), 10% 2-mercaptoethanol, 20% glycerol, 0.004% bromophenol blue, and 0.125 M tris HCl (pH 6.8)]. Then, the whole protein solution (15 μg protein for each sample) was applied in SDS–polyacrylamide gel electrophoresis (15% resolving gel and 15% stacking gel) at 80 V for about 150 min and transferred to polyvinylidene difluoride membrane at 110 V for 80 min. The membrane was blocked with 5% (*w*/*v*) nonfat milk powder in TBST (tris buffer saline with 0.1% Tween-20) for 2 hours at room temperature and incubated with the primary antibodies overnight at 4°C. The membrane was washed three times with 4% milk tris buffer saline. After incubation of horseradish peroxidase–linked secondary antibody for 1 hour, the membrane was washed three times with TBST. Then, the membrane was incubated with enhanced chemiluminescence (ECL) reagent (Bio-Rad) for 10 min and imaged with the Bio-Rad ChemiDoc Touch Imaging System. The intensity of bands was quantified by ImageJ, and the intensity was normalized with protein loading amount. For the calculation of relative expression level, the vehicle control group was set as 100%. The experiment was completed with biological triplicates. For CLSM imaging, the cells with the same condition in groups 1, 6, and 9 were prepared and stained with ER tracker and Mito-tracker for further observations.

### Animal model and animal ethics

All the mice were obtained from the City University of Hong Kong and cultured under pathogen-free conditions. All animal-related experiments were carried out following the National Institutes of Health guidelines for the care and use of laboratory animals and approved by the Animal Ethics Sub-Committee of the City University of Hong Kong (A0594), under licensed Animal (Control of Experiments) Ordinance Chapter 340 [ref. no. (19-143) in DH/SHS/8/2/5 Pt.6]. To establish tumor-bearing mouse models, female BALB/c mice (8 weeks old) were subcutaneously injected with 50 μl of suspension of 2 × 10^6^ 4T1 cells at the shaved right flank. After 10 days, the mean tumor volume exceeded 120 mm^3^ for further use, and the grouping of mice was fully randomized.

### Maximum tolerated dose test

To test the biocompatibility of cyaninplatin with systemic administration, BALB/c mice were randomly divided into three groups (*n* = 3) and intravenously injected with cyaninplatin at a dosage of 0, 1.5, or 3.0 mg kg^−1^ (Pt amount to bodyweight). During the following 14 days, body weight and change of behavior were monitored. The highest dose of 3.0 mg kg^−1^ already reached the solubility limit, which was dissolved in 200 μl of physiological saline (0.9% NaCl) containing 0.5% DMF and 4.5% Kolliphor HS 15. If obvious bodyweight loss (more than 20%), debilitating effects, or signs of distress occurred, then the dosage would be deemed as the maximum tolerated dose (MTD).

### Pharmacokinetic assay

Tumor-bearing BALB/c mice (*n* = 3) were intravenously injected with 200 μl of cyaninplatin (at a dosage of about 0.3 mg Pt/kg bodyweight). At time points of 0.5, 1.5, 6, 12, 24, and 48 hours after injection, about 20 μl of orbital blood was collected with weighing and digested with aqua regia for pharmacokinetics analysis via ICP-MS for Pt element.

### Biodistribution test

At 6, 12 or 48 hours after injection of cyaninplatin (0.5 mg Pt/ kg body weight, *n* = 3), tumor-bearing mice were sacrificed. Main organ tissues (heart, liver, spleen, lung, and kidney) and tumors were dissected and digested (aqua regia, 80°C, 48 hours) for ICP analysis to determine the residue amount of Pt.

### In vivo fluorescence imaging

To monitor the accumulation status of cyaninplatin in the tumor, tumor-bearing BALB/c mice (*n* = 3) were intravenously injected with cyaninplatin at a dosage of 3 mg Pt/ kg bodyweight. At determined time intervals (0, 1, 3, 6, 9, 12, and 24 hours), optical imaging was conducted (780 nm ex./830 nm em.), and fluorescent signal was quantified with a region of interest.

### In vivo ultrasound/photoacoustic imaging

The imaging system was used as previously reported (*60*). The mice were placed under gas anesthesia by isoflurane. By 20 step-scan of transverse-section planes of mice (1 mm between each plane), the 3D geometric feature of the tumor area was reconstructed. Then, time-dependent photoacoustic computed tomography was conducted to determine therapeutic time windows for further SSCT.

### In vivo anticancer evaluation

Tumor-bearing BALB/c mice were randomly divided into six groups by intravenous injection with different drugs (*n* = 5 in each group): (i) vehicle control (saline +4.5% Kolliphor HS 15 + 0.5% DMF); (ii) carboplatin + ligand (1:1 molar ratio, 3 mg Pt/kg); (iii) cyaninplatin (3 mg Pt/kg); (iv) ultrasound (3.5 W); (v) carboplatin + ligand + ultrasound; (vi) cyaninplatin + ultrasound (fig. S37A).

For ultrasound treatment, the mice were under anesthesia by isoflurane, and the tumor was immersed in a clean water bath (37°C) to maintain the body temperature. Since tumor size was visualized with ultrasound imaging, three focal positions were set around the tumor center. Tumors were subjected to scanning treatment (fig. S37B): The ultrasound focus will repeatedly move among the three focal positions (3.5 W, 2-min treatment with 1-min interval to prevent overheating of tissue, repeated for 10 times in one treatment). During ultrasound treatment, the local tumoral temperature was monitored with a thermal camera (FLIR Systems Inc.) to prevent local hyperthermia. For ultrasound-treated groups, the treatment was conducted every 2 days and repeated for four times. After 18 days of treatment, all the mice were sacrificed by cervical dislocation. Main organ tissues were dissected, and full blood was collected by enucleation of the eye. Tumor volume was calculated following equation: *V* (mm^3^) = length × width^2^ × 0.5. The relative mean tumor volume at day 0 was defined as 100% for each group.

### Blood chemistry assay

The collected blood was centrifugated at 800*g* for 10 min and 12,000*g* to obtain the supernatant as serum samples. Then, aspartate transaminase/serum glutamic-oxaloacetic transaminase, alanine aminotransferase/serum glutamic-pyruvic transaminase, blood urea nitrogen, and creatine kinase myocardial band were analyzed by corresponding Stanbio Labs kits following the manufacturer’s instructions.

### Histological pathology analysis

The dissected organs and tumors were fixed with 10% paraformaldehyde at room temperature for 24 hours. Then, all the tissues were dehydrated with gradient ethanol (50, 70, 80, 95, and 100%), cleared with xylene, and embedded in paraffin. The embedded tissues were cut into 5-μm-thick sections for staining with H&E or immunohistology analysis.

### In vivo tissue penetration test

Chicken breast tissues (sliced into 10 cm by 4 cm by 4 cm) were prepared and injected with 20 μl of a mixture of cyaninplatin (15 μM) and SOSG (20 μM) at the position of 1, 3, 5, and 7 cm from the edge. Then, the tissue samples were treated with 808-nm laser (0.33 W cm^−2^, 10 min) or FUS (Chattanooga CHA-15-1201 Mobile 2, 1, or 3 MHz, 3.5 W, 10 min). The signal increase (ΔIntensity_SOSG_ = Intensity_after_ − Intensity_before_) of SOSG was obtained by region of interest (480 nm ex./ 530 nm em.) to represent the activation efficiency of cyaninplatin. In each three groups, for comparison of energy decay over tissue depth, ΔIntensity_SOSG_ of each sample in test tube without any tissue coverage was defined as 100%.

For in vivo penetration assay, 4T1 tumor–bearing mice (*n* = 3) were intratumorally injected with 20 μl of a mixed solution of dihydroethidium (10 μg/ml) and cyaninplatin (15 μM) in PBS (pH 7.4). Then, the FUS (1 MHz, 3.5 W, 10 min) was applied on the opposite side of the tumor, and fluorescent signal of ethidium at the tumor region was recorded (520 nm ex./620 nm em.).

### Statistical analysis

The errors of parallel test were presented as mean ± SD. OriginPro 2017 (OriginLab), GraphPad Prism 8, and ImageJ 1.53c were used for data analysis. Statistical difference was calculated through two-tailed Student’s *t* test: *P* > 0.05 not significant (N.S.), *P < 0.05, **P < 0.01, ***P < 0.001.

## Notes

View/request a protocol for this paper from *Bio-protocol*.

## References

[1] K. A. Ryu, C. M. Kaszuba, N. B. Bissonnette, R. C. Oslund, O. O. Fadeyi, Interrogating biological systems using visible-light-powered catalysis. Nat. Rev. Chem. 5, 322–337 (2021). 37117838 10.1038/s41570-021-00265-6

[2] D. Havrylyuk, A. C. Hachey, A. Fenton, D. K. Heidary, E. C. Glazer, Ru(II) photocages enable precise control over enzyme activity with red light. Nat. Commun. 13, 3636 (2022). 35752630 10.1038/s41467-022-31269-5 PMC9233675

[3] J. Rautio, H. Kumpulainen, T. Heimbach, R. Oliyai, D. Oh, T. Järvinen, J. Savolainen, Prodrugs: Design and clinical applications. Nat. Rev. Drug Discov. 7, 255–270 (2008). 18219308 10.1038/nrd2468

[4] J. Rautio, N. A. Meanwell, L. Di, M. J. Hageman, The expanding role of prodrugs in contemporary drug design and development. Nat. Rev. Drug Discov. 17, 559–587 (2018). 29700501 10.1038/nrd.2018.46

[5] N. J. Farrer, J. A. Woods, L. Salassa, Y. Zhao, K. S. Robinson, G. Clarkson, F. S. MacKay, P. J. Sadler, A potent *trans*-diimine platinum anticancer complex photoactivated by visible light. Angew. Chem. Int. Ed. 49, 8905–8908 (2010). 10.1002/anie.201003399 20677309

[6] C. Imberti, P. Zhang, H. Huang, P. J. Sadler, New designs for phototherapeutic transition metal complexes. Angew. Chem. Int. Ed. 59, 61–73 (2020). 10.1002/anie.201905171 PMC6973108 31310436

[7] E. M. Bolitho, C. Sanchez-Cano, H. Shi, P. D. Quinn, M. Harkiolaki, C. Imberti, P. J. Sadler, Single-cell chemistry of photoactivatable platinum anticancer complexes. J. Am. Chem. Soc. 143, 20224–20240 (2021). 34808054 10.1021/jacs.1c08630 PMC8662725

[8] T. W. Hambley, The influence of structure on the activity and toxicity of Pt anti-cancer drugs. Coord. Chem. Rev. 166, 181–223 (1997).

[9] E. Wexselblatt, E. Yavin, D. Gibson, Cellular interactions of platinum drugs. Inorganica Chim. Acta 393, 75–83 (2012).

[10] L. Kelland, The resurgence of platinum-based cancer chemotherapy. Nat. Rev. Cancer 7, 573–584 (2007). 17625587 10.1038/nrc2167

[11] D. Wang, S. J. Lippard, Cellular processing of platinum anticancer drugs. Nat. Rev. Drug Discov. 4, 307–320 (2005). 15789122 10.1038/nrd1691

[12] T. Yempala, T. Babu, S. Karmakar, A. Nemirovski, M. Ishan, V. Gandin, D. Gibson, Expanding the arsenal of Pt^IV^ anticancer agents: Multi-action Pt^IV^ anticancer agents with bioactive ligands possessing a hydroxy functional group. Angew. Chem. Int. Ed. 58, 18218–18223 (2019). 10.1002/anie.201910014 31599054

[13] Z. Xu, Z. Wang, Z. Deng, G. Zhu, Recent advances in the synthesis, stability, and activation of platinum(IV) anticancer prodrugs. Coord. Chem. Rev. 442, 213991 (2021).

[14] T. C. Johnstone, K. Suntharalingam, S. J. Lippard, The next generation of platinum drugs: Targeted Pt(II) agents, nanoparticle delivery, and Pt(IV) prodrugs. Chem. Rev. 116, 3436–3486 (2016). 26865551 10.1021/acs.chemrev.5b00597 PMC4792284

[15] G. Thiabaud, G. He, S. Sen, K. A. Shelton, W. B. Baze, L. Segura, J. Alaniz, R. M. MacIas, G. Lyness, A. B. Watts, H. M. Kim, H. Lee, M. Y. Cho, K. S. Hong, R. Finch, Z. H. Siddik, J. F. Arambula, J. L. Sessler, Oxaliplatin Pt(IV) prodrugs conjugated to gadolinium-texaphyrin as potential antitumor agents. Proc. Natl. Acad. Sci. U.S.A. 117, 7021–7029 (2020). 32179677 10.1073/pnas.1914911117 PMC7132275

[16] Z. Wang, N. Wang, S. C. Cheng, K. Xu, Z. Deng, S. Chen, Z. Xu, K. Xie, M. K. Tse, P. Shi, H. Hirao, C. C. Ko, G. Zhu, Phorbiplatin, a highly potent Pt(IV) antitumor prodrug that can be controllably activated by red light. Chem 5, 3151–3165 (2019).

[17] Z. Deng, N. Wang, Y. Liu, Z. Xu, Z. Wang, T. C. Lau, G. Zhu, A photocaged, water-oxidizing, and nucleolus-targeted Pt(IV) complex with a distinct anticancer mechanism. J. Am. Chem. Soc. 142, 7803–7812 (2020). 32216337 10.1021/jacs.0c00221

[18] X. Li, J. F. Lovell, J. Yoon, X. Chen, Clinical development and potential of photothermal and photodynamic therapies for cancer. Nat. Rev. Clin. Oncol. 17, 657–674 (2020). 32699309 10.1038/s41571-020-0410-2

[19] M. Rudin, R. Weissleder, Molecular imaging in drug discovery and development. Nat. Rev. Drug Discov. 2, 123–131 (2003). 12563303 10.1038/nrd1007

[20] J. Geng, Y. Zhang, Q. Gao, K. Neumann, H. Dong, H. Porter, M. Potter, H. Ren, D. Argyle, M. Bradley, Switching on prodrugs using radiotherapy. Nat. Chem. 13, 805–810 (2021). 34112990 10.1038/s41557-021-00711-4 PMC7611443

[21] S. Mitragotri, Healing sound: The use of ultrasound in drug delivery and other therapeutic applications. Nat. Rev. Drug Discov. 4, 255–260 (2005). 15738980 10.1038/nrd1662

[22] X. Wang, G. Kim, J. L. Chu, T. Song, Z. Yang, W. Guo, X. Shao, M. L. Oelze, K. C. Li, Y. Lu, Noninvasive and spatiotemporal control of DNAzyme-based imaging of metal ions *in vivo* using high-intensity focused ultrasound. J. Am. Chem. Soc. 144, 5812–5819 (2022). 35302361 10.1021/jacs.1c11543 PMC9133526

[23] M. A. Santos, S. K. Wu, M. Regenold, C. Allen, D. E. Goertz, K. Hynynen, Novel fractionated ultrashort thermal exposures with MRI-guided focused ultrasound for treating tumors with thermosensitive drugs. Sci. Adv. 6, eaba5684 (2020). 32917589 10.1126/sciadv.aba5684 PMC7467687

[24] S. Dromi, V. Frenkel, A. Luk, B. Traughber, M. Angstadt, M. Bur, J. Poff, J. Xie, S. K. Libutti, K. C. P. Li, B. J. Wood, Pulsed-high intensity focused ultrasound and low –Temperature-sensitive liposomes for enhanced targeted drug delivery and antitumor effect. Clin. Cancer Res. 13, 2722–2727 (2007). 17473205 10.1158/1078-0432.CCR-06-2443 PMC2555974

[25] S. Huo, P. Zhao, Z. Shi, M. Zou, X. Yang, E. Warszawik, M. Loznik, R. Göstl, A. Herrmann, Mechanochemical bond scission for the activation of drugs. Nat. Chem. 13, 131–139 (2021). 33514936 10.1038/s41557-020-00624-8

[26] A. Y. Rwei, J. L. Paris, B. Wang, W. Wang, C. D. Axon, M. Vallet-Regí, R. Langer, D. S. Kohane, Ultrasound-triggered local anaesthesia. Nat. Biomed. Eng. 1, 644–653 (2017). 29152410 10.1038/s41551-017-0117-6 PMC5687284

[27] A. Schroeder, R. Honen, K. Turjeman, A. Gabizon, J. Kost, Y. Barenholz, Ultrasound triggered release of cisplatin from liposomes in murine tumors. J. Control. Release 137, 63–68 (2009). 19303426 10.1016/j.jconrel.2009.03.007

[28] N. McDannold, Y. Zhang, J. G. Supko, C. Power, T. Sun, C. Peng, N. Vykhodtseva, A. J. Golby, D. A. Reardon, Acoustic feedback enables safe and reliable carboplatin delivery across the blood-brain barrier with a clinical focused ultrasound system and improves survival in a rat glioma model. Theranostics 9, 6284–6299 (2019). 31534551 10.7150/thno.35892 PMC6735504

[29] H. X. Li, J. H. Zheng, L. Ji, G. Y. Liu, Y. K. Lv, D. Yang, Z. Hu, H. Chen, F. M. Zhang, W. Cao, Effects of low-intensity ultrasound combined with low-dose carboplatin in an orthotopic hamster model of tongue cancer: A preclinical study. Oncol. Rep. 39, 1609–1618 (2018). 29436690 10.3892/or.2018.6262 PMC5868397

[30] B. H. A. Lammertink, C. Bos, K. M. van der Wurff-Jacobs, G. Storm, C. T. Moonen, R. Deckers, Increase of intracellular cisplatin levels and radiosensitization by ultrasound in combination with microbubbles. J. Control. Release 238, 157–165 (2016). 27476609 10.1016/j.jconrel.2016.07.049

[31] N. Chang, D. Qin, P. Wu, S. Xu, S. Wang, M. Wan, IR780 loaded perfluorohexane nanodroplets for efficient sonodynamic effect induced by short-pulsed focused ultrasound. Ultrason. Sonochem. 53, 59–67 (2019). 30559082 10.1016/j.ultsonch.2018.12.021

[32] Y. Wang, X. Liao, J. Sun, B. Yi, S. Luo, T. Liu, X. Tan, D. Liu, Z. Chen, X. Wang, C. Shi, Characterization of HIF-1α/glycolysis hyperactive cell population via small-molecule-based imaging of mitochondrial transporter activity. Adv. Sci. 5, 1700392 (2018). 10.1002/advs.201700392 PMC5867035 29593950

[33] E. Zhang, S. Luo, X. Tan, C. Shi, Mechanistic study of IR-780 dye as a potential tumor targeting and drug delivery agent. Biomaterials 35, 771–778 (2014). 24148240 10.1016/j.biomaterials.2013.10.033

[34] E. Wexselblatt, R. Raveendran, S. Salameh, A. Friedman-Ezra, E. Yavin, D. Gibson, On the stability of Pt^IV^ pro-drugs with haloacetato ligands in the axial positions. Chem. A Eur. J. 21, 3108–3114 (2015). 10.1002/chem.201405467 25529335

[35] C. K. J. Chen, X. Gui, P. Kappen, A. K. Renfrew, T. W. Hambley, The effect of charge on the uptake and resistance to reduction of platinum(IV) complexes in human serum and whole blood models. Metallomics 12, 1599–1615 (2020). 33084707 10.1039/d0mt00157k

[36] H. Huang, S. Banerjee, K. Qiu, P. Zhang, O. Blacque, T. Malcomson, M. J. Paterson, G. J. Clarkson, M. Staniforth, V. G. Stavros, G. Gasser, H. Chao, P. J. Sadler, Targeted photoredox catalysis in cancer cells. Nat. Chem. 11, 1041–1048 (2019). 31548671 10.1038/s41557-019-0328-4

[37] Y. Mizukoshi, E. Takagi, H. Okuno, R. Oshima, Y. Maeda, Y. Nagata, Preparation of platinum nanoparticles by sonochemical reduction of the Pt(IV) ions: Role of surfactants. Ultrason. Sonochem. 8, 1–6 (2001). 11105315 10.1016/s1350-4177(00)00027-4

[38] N. Xie, L. Zhang, W. Gao, C. Huang, P. E. Huber, X. Zhou, C. Li, G. Shen, B. Zou, NAD^+^ metabolism: Pathophysiologic mechanisms and therapeutic potential. Signal Transduct. Target. Ther. 5, 227 (2020). 33028824 10.1038/s41392-020-00311-7 PMC7539288

[39] J. J. Soldevila-Barreda, I. Romero-Canelón, A. Habtemariam, P. J. Sadler, Transfer hydrogenation catalysis in cells as a new approach to anticancer drug design. Nat. Commun. 6, 6582 (2015). 25791197 10.1038/ncomms7582 PMC4383003

[40] J. P. C. Coverdale, I. Romero-Canelón, C. Sanchez-Cano, G. J. Clarkson, A. Habtemariam, M. Wills, P. J. Sadler, Asymmetric transfer hydrogenation by synthetic catalysts in cancer cells. Nat. Chem. 10, 347–354 (2018). 29461524 10.1038/nchem.2918

[41] M. V. Babak, Y. Zhi, B. Czarny, T. B. Toh, L. Hooi, E. K. H. Chow, W. H. Ang, D. Gibson, G. Pastorin, Dual-targeting dual-action platinum(IV) platform for enhanced anticancer activity and reduced nephrotoxicity. Angew. Chem. Int. Ed. 58, 8109–8114 (2019). 10.1002/anie.201903112 30945417

[42] X. Ling, J. Tu, J. Wang, A. Shajii, N. Kong, C. Feng, Y. Zhang, M. Yu, T. Xie, Z. Bharwani, B. M. Aljaeid, B. Shi, W. Tao, O. C. Farokhzad, Glutathione-responsive prodrug nanoparticles for effective drug delivery and cancer therapy. ACS Nano 13, 357–370 (2019). 30485068 10.1021/acsnano.8b06400 PMC7049173

[43] H. Yao, S. Chen, Z. Deng, M. K. Tse, Y. Matsuda, G. Zhu, BODI-Pt, a green-light-activatable and carboplatin-based platinum(IV) anticancer prodrug with enhanced activation and cytotoxicity. Inorg. Chem. 59, 11823–11833 (2020). 32799491 10.1021/acs.inorgchem.0c01880

[44] R. A. Cairns, I. S. Harris, T. W. Mak, Regulation of cancer cell metabolism. Nat. Rev. Cancer 11, 85–95 (2011). 21258394 10.1038/nrc2981

[45] G. Liu, J. Zhu, H. Guo, A. Sun, P. Chen, L. Xi, W. Huang, X. Song, X. Dong, Mo_2_C-derived polyoxometalate for NIR-II photoacoustic imaging-guided chemodynamic/photothermal synergistic therapy. Angew. Chem. Int. Ed. 58, 18641–18646 (2019). 10.1002/anie.201910815 31605417

[46] S. G. Kimani, J. B. Phillips, J. I. Bruce, A. J. MacRobert, J. P. Golding, Antioxidant inhibitors potentiate the cytotoxicity of photodynamic therapy. Photochem. Photobiol. 88, 175–187 (2012). 22044030 10.1111/j.1751-1097.2011.01022.x

[47] G. G. Yang, Z. Y. Pan, D. Y. Zhang, Q. Cao, L. N. Ji, Z. W. Mao, Precisely assembled nanoparticles against cisplatin resistance via cancer-specific targeting of mitochondria and imaging-guided chemo-photothermal therapy. ACS Appl. Mater. Interfaces 12, 43444–43455 (2020). 32883070 10.1021/acsami.0c12814

[48] G. G. Yang, X. X. Su, B. B. Liang, Z. Y. Pan, Q. Cao, Z. W. Mao, A platinum-ruthenium hybrid prodrug with multi-enzymatic activities for chemo-catalytic therapy of hypoxic tumors. Chem. Sci. 13, 11360–11367 (2022). 36320579 10.1039/d2sc03375e PMC9533400

[49] X. Su, B. Liu, W. J. Wang, K. Peng, B. B. Liang, Y. Zheng, Q. Cao, Z. W. Mao, Disruption of zinc homeostasis by a novel platinum(IV)-terthiophene complex for antitumor immunity. Angew. Chem. Int. Ed. 62, e2022169 (2022). 10.1002/anie.202216917 36546893

[50] Z. Deng, C. Li, S. Chen, Q. Zhou, Z. Xu, Z. Wang, H. Yao, H. Hirao, G. Zhu, An intramolecular photoswitch can significantly promote photoactivation of Pt(IV) prodrugs. Chem. Sci. 12, 6536–6542 (2021). 34040729 10.1039/d0sc06839j PMC8139284

[51] J. H. van den Berg, J. H. Beijnen, A. J. M. Balm, J. H. M. Schellens, Future opportunities in preventing cisplatin induced ototoxicity. Cancer Treat. Rev. 32, 390–397 (2006). 16781082 10.1016/j.ctrv.2006.04.011

[52] L. Galluzzi, A. Buqué, O. Kepp, L. Zitvogel, G. Kroemer, Immunogenic cell death in cancer and infectious disease. Nat. Rev. Immunol. 17, 97–111 (2017). 27748397 10.1038/nri.2016.107

[53] W. Um, H. Ko, D. G. You, S. Lim, G. Kwak, M. K. Shim, S. Yang, J. Lee, Y. Song, K. Kim, J. H. Park, Necroptosis-inducible polymeric nanobubbles for enhanced cancer sonoimmunotherapy. Adv. Mater. 32, 1907953 (2020). 10.1002/adma.201907953 32125731

[54] D. Kessel, Pathways to paraptosis After ER photodamage in OVCAR‐5 cells. Photochem. Photobiol. 95, 1239–1242 (2019). 30924537 10.1111/php.13103

[55] D. Kessel, Apoptosis, paraptosis and autophagy: Death and survival pathways associated with photodynamic therapy. Photochem. Photobiol. 95, 119–125 (2019). 29882356 10.1111/php.12952 PMC6286706

[56] S. Sperandio, K. Poksay, I. de Belle, M. J. Lafuente, B. Liu, J. Nasir, D. E. Bredesen, Paraptosis: Mediation by MAP kinases and inhibition by AIP-1/Alix. Cell Death Differ. 11, 1066–1075 (2004). 15195070 10.1038/sj.cdd.4401465

[57] B. A. Pulaski, S. Ostrand-Rosenberg, Mouse 4T1 breast tumor model. Curr. Protoc. Immunol. 39, 1–16 (2000). 10.1002/0471142735.im2002s39 18432775

[58] M. P. Barr, S. G. Gray, A. C. Hoffmann, R. A. Hilger, J. Thomale, J. D. O’Flaherty, D. A. Fennell, D. Richard, J. J. O’Leary, K. J. O’Byrne, Generation and characterisation of cisplatin-resistant non-small cell lung cancer cell lines displaying a stem-like signature. PLOS ONE 8, e54193 (2013). 23349823 10.1371/journal.pone.0054193 PMC3547914

[59] Y. Liu, W. Zhen, Y. Wang, J. Liu, L. Jin, T. Zhang, S. Zhang, Y. Zhao, S. Song, C. Li, J. Zhu, Y. Yang, H. Zhang, One-dimensional Fe_2_P acts as a fenton agent in response to NIR II light and ultrasound for deep tumor synergetic theranostics. Angew. Chem. Int. Ed. 58, 2407–2412 (2019). 10.1002/anie.201813702 30600877

[60] Y. Zhang, Y. Wang, P. Lai, L. Wang, Video-rate dual-modal wide-beam harmonic ultrasound and photoacoustic computed tomography. IEEE Trans. Med. Imaging 41, 727–736 (2022). 34694993 10.1109/TMI.2021.3122240

