# Supplementary material for: An ultrasound-activatable platinum prodrug for sono-sensitized chemotherapy
Source: Sci Adv. 2023 Jun 21;9(25):eadg5964. doi: 10.1126/sciadv.adg5964 (PMC10284555; doi:10.1126/sciadv.adg5964)
Supplement: Supplementary file 1 — Supplementary Materials and Methods Fig. S1 to S45 Table S1 [file sciadv.adg5964_sm.pdf]

Science Advances
AAAS

Supplementary Materials for

An ultrasound-activatable platinum prodrug for sono-sensitized chemotherapy

Gongyuan Liu *et al.*

Corresponding author: Lidai Wang, lidawang@cityu.edu.hk; Guangyu Zhu, guangzhu@cityu.edu.hk

Sci. Adv. **9**, eadg5964 (2023)
DOI: 10.1126/sciadv.adg5964

This PDF file includes:

Supplementary Materials and Methods
Fig. S1 to S45
Table S1

Supplementary Text

Materials and Reagents.

IR780 iodide, hydrogen peroxide (30% or 50% in water), acetic acid, calcein-AM, propidium iodide (PI), JC-1, sodium pyruvate (NaPyr), sodium azide (NaN_3), and sodium ascorbate (NaAsc) were obtained from Sigma Aldrich. Sodium hydride (NaH, 60% dispersion in mineral oil), celastrol, N-acetyl cysteine (NAC), and 3-(4-hydroxyphenyl)propionic acid were obtained from Energy Chemical (China). Cisplatin, carboplatin, and oxaliplatin were obtained from Boyuan Technology (Shandong, China). Dihydroethidium (DHE), 2',7'-dichlorodihydrofluorescein diacetate (DCFH-DA), 3-(4,5-dimethyl-2-thiazolyl)-2,5-diphenyl-2H-tetrazolium bromide (MTT), minimum essential medium (MEM), Dulbecco's Modified Eagle Medium (DMEM), Roswell Park Memorial Institute 1640 (RPMI 1640) medium, trypsin, phosphate-buffered saline (PBS), fetal bovine serum (FBS), and penicillin-streptomycin were purchased from Life Technologies. Unless otherwise specified, PBS used in this study contains 10 mM Na_2HPO_4, 2 mM KH_2PO_4, 137 mM NaCl, and 2.7 mM KCl, with pH value of 7.4. Graphic resources for schematic illustrations were obtained from smart.servier.com under permission of usage.

ATP Determination Kit (A22066), ER-Tracker™ Blue-White DPX (E12353), LysoTracker™ Green DND-26 (L7526), and MitoTracker™ Green FM (M7514) were purchased from Thermo Fisher Scientific. Rabbit polyclonal calreticulin (CRT) antibody (Cat. No.: ab2907, RRID: AB_303402), rabbit monoclonal anti-HMGB1 antibody (ab79823, AB_1603373), and goat anti-rabbit lgG H&L (FITC) secondary antibody (ab7086, AB_955262) were purchased from Abcam. Triton X-100 cell lysis buffer (P0013), GSH/GSSG detection kit (S0053), and NADH/NAD^+ detection kit (S0175) were purchased from Beyotime (China). Mouse anti-CHOP primary antibody (CST#2895, AB_2089254), mouse anti-Alix primary antibody (CST#2171, AB_2299455), rabbit anti-β-actin mab (CST#4970, AB_2223172), Anti-mouse IgG, HRP-linked Antibody (CST#7076, AB_330924), protein marker (CST#74124), and Anti-rabbit IgG, HRP-linked Antibody (CST#7074, AB_2099233) were obtained from Cell Signaling Technology.

Instruments.

Nuclear Magnetic Resonance (NMR) data were recorded with Bruker Ascend AVANCE III (300 MHz, 400 MHz, or 600 MHz) spectrometers at room temperature with samples protected from light. Chemical shifts are reported in parts per million (ppm), calibrated to solvent peaks. UV-Vis absorbance was recorded on Shimadzu 1700 double beam scanning UV-Vis spectrophotometer (Shimadzu, Japan). Fluorescence spectra were recorded with Horiba Fluormax-4 Spectrofluorometer (HORIBA Scientific, Japan). Circular Dichroism (CD) spectra were recorded with CD Spectrometer (JASCO J-150, Japan). Electron paramagnetic resonance (EPR) studies were carried out with ADANI SPINSCAN X EPR spectrometer. The confocal cell imaging was conducted with Laser Confocal Scanning Microscopes (Leica SPE and Leica SP5). Analytical reversed-phase High-Performance Liquid Chromatography (RP-HPLC) was conducted on a Shimadzu Prominence LC-20AT HPLC system equipped with a reversed-phase C18 column (Phenomenex Garmin 250 × 4.60 mm, 5 μm, 110 Å), and LC-MS analysis was conducted with HPLC-High Resolution Mass Spectrometer (Sciex X500R Q-TOF). Unless indicated, test samples were monitored by UV-Vis absorbance at 700 nm: Solvent A (H_2O with 5% acetonitrile and 0.1% formic acid) and solvent B (acetonitrile with 5% H_2O and 0.1% formic acid) were used for a gradient elution at a flow rate of 1.0 mL min^{-1}. The test samples were eluted following the program: 5% B (0 min) → 20% B (7 min) → 90% B (16 min) → 90% B (25 min). Optical imaging for mice was performed on an IVIS Lumina III *In Vivo* Imaging system (PerkinElmer, USA).

Synthesis of cis,trans-diammine(cyclobutane-1,1-dicarboxylato)dihydroxidoplatinum(IV) [oxo-carboPt(IV)]. For a typical process, carboplatin (1.00 g, 2.70 mmol) was suspended in 15 mL Milli-Q water, and subsequently 1.21 mL H_2O_2 solution (30% in water) was added. After heating for 6 h at 328 K, the reaction was cooled to room temperature, and solvent was removed under reduced pressure. After washing with acetone for three times, pale powder product was obtained as oxo-carboPt(IV) (0.98 g, 90.0%, marked as complex **1**). ESI-MS (m/z): $[M-H]^-$ calculated for $C_6H_{13}N_2O_6Pt^-$: 404.2, found: 404.2.

Synthesis of cis,trans-diammine(cyclobutane-1,1-dicarboxylato)acetatohydroxido-platinum(IV) (complex 2). To synthesize complex **2**, carboplatin (50 mg) was dissolved in 20 mL acetic acid with the addition of H_2O_2 (0.5 mL, 50% in water) and stirred for 30 min at room temperature. Upon the completion of the reaction, the crude product was obtained by precipitation in Et_2O. After recrystallization in acetone, white solid was obtained as complex **2**. ^{1}H NMR (600 MHz, DMSO-d_6) δ 6.11 – 5.70 (m, 6H), 2.57 – 2.53 (t, J = 8.0 Hz, 2H), 2.48 (t, J = 8.0 Hz, 2H), 1.87 (s, 3H), 1.79 (p, J = 8.0 Hz, 2H). ^{13}C NMR (151 MHz, DMSO-d_6) δ 179.03, 175.79, 56.14, 33.34, 30.33, 24.30, 15.04. ^{195}Pt NMR (129 MHz, DMSO-d_6) δ 1753.33. ESI-MS (m/z): $[M-H]^-$ calculated for $C_8H_{15}N_2O_7Pt^-$: 446.1, found: 446.4; $[M+Cl]^-$ calculated for $C_8H_{16}ClN_2O_7Pt^-$: 482.0, found: 481.9.

Synthesis of ligand 1. NaH (60% in mineral oil, 19.3 mg, 0.270 mmol) was dissolved in 3 mL dry DMF and 3-(4-Hydroxyphenyl)propionic acid (39.9 mg, 0.150 mmol in 2 mL dry DMF) was added dropwise in ice bath and under N_2 protection. Then, IR780 iodide (40.0 mg, 0.060 mmol, in 5 mL dry DMF) was slowly added during 30 min. The reaction was further kept at 298 K for another 1 h. After washing with Et_2O and silica column chromatography (eluent: DCM/MeOH= 8/1), reddish-green crystalline solid was obtained as ligand **1** (44.2 mg, 92.7%). ^{1}H NMR (300 MHz, Methanol-d_4) δ(ppm) 8.01 (d, *J* = 14.1 Hz, 2H), 7.44 – 7.34 (m, 4H), 7.32 – 7.18 (m, 6H), 7.09 – 7.01 (m, 2H), 6.17 (d, *J* = 14.1 Hz, 2H), 4.09 (t, *J* = 7.2 Hz, 4H), 2.81 (dt, *J* = 34.1, 6.9 Hz, 6H), 2.54 (t, *J* = 6.9 Hz, 2H), 2.11 – 2.01 (m, 2H), 1.85 (q, *J* = 7.5 Hz, 4H), 1.35 (s, 12H), 1.03 (t, *J* = 7.5 Hz, 6H). HRMS (m/z): $[M]^+$ calculated for $C_{45}H_{53}N_2O_3^+$: 669.4051, found: 669.3998.

Synthesis of cyaninplatin. Oxo-carboPt(IV) (20.2 mg, 0.05 mmol), ligand **1** (10.0 mg, 0.05 mmol), 2-(1H-benzotriazole-1-yl)-1,1,3,3-tetramethylaminium tetrafluoroborate (TBTU, 19.2 mg, 0.06 mmol), and N,N-diisopropylethylamine (DIEA, 9.2 mg, 0.06 mmol) were dissolved in 2 mL dry DMSO. The reaction was kept for 24 h at 298 K in the dark, then 10 mL of cold water was added to precipitate the yielded product. After silica column chromatography (eluent: DCM/MeOH= 6/1) and recrystallization with DCM/Et_2O = 1/3, dark green powder was obtained as cyaninplatin (22.4 mg, 74.6%). ^{1}H NMR (300 MHz, DMSO-d_6) δ 7.80 (d, J = 14.2 Hz, 1H), 7.50 (d, J = 7.5 Hz, 1H), 7.41 – 7.33 (m, 2H), 7.25 – 7.16 (m, 2H), 7.03 (d, J = 8.7 Hz, 1H), 6.17 (d, J = 14.2 Hz, 1H), 4.10 (t, J = 7.5 Hz, 3H), 2.68 (d, J = 6.9 Hz, 3H), 2.43 – 2.29 (m, 1H), 2.29 (t, J = 6.0 Hz, 1H), 1.93 (d, J = 6.3 Hz, 1H), 1.70 (dt, J = 14.3, 7.2 Hz, 3H), 0.92 (t, J = 7.5 Hz, 4H). ^{13}C NMR (151 MHz, DMSO-d_6) δ(ppm) 180.01, 172.16, 163.35, 158.40, 158.34, 141.37, 136.06, 130.41, 128.93, 125.22, 122.87, 121.87, 111.71, 49.02, 32.26, 31.80, 31.25, 27.68, 21.19, 20.82, 16.19, 15.95, 11.55. ^{195}Pt NMR (129 MHz, DMF-d_7) δ(ppm) 1735.00. HRMS (m/z): $[M]^+$ calculated for $C_{51}H_{65}N_4O_8Pt^+$: 1056.4445, found: 1056.4399. Powder of cyaninplatin was stored

at 193 K, and solution stock was prepared in DMF (8 mM) and stored at 253 K, avoiding any light exposure for further use, and purity was checked with HPLC before use.

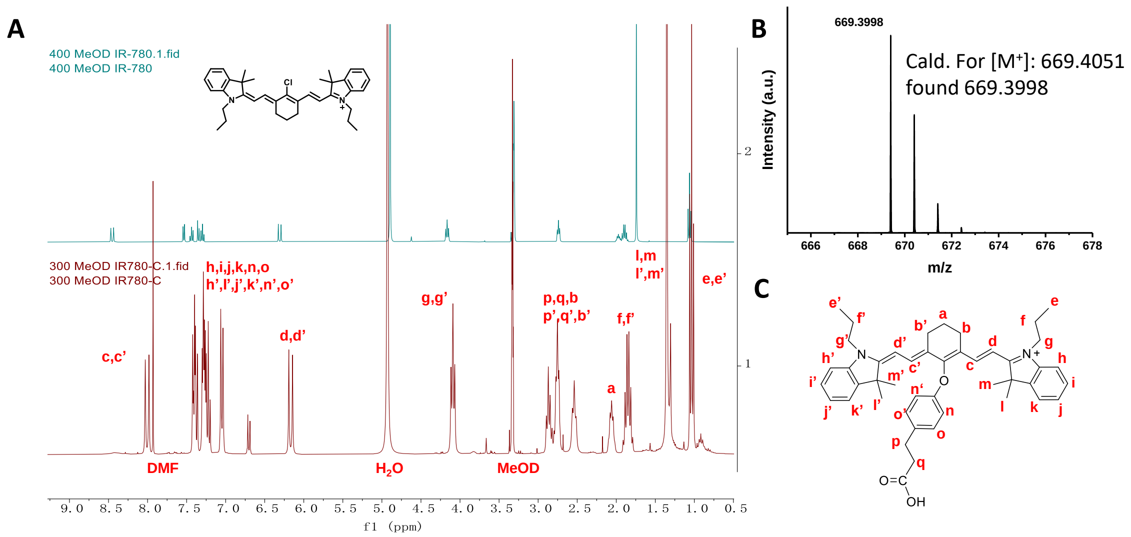

Fig. S1. (A) ^{1}H NMR spectra of IR780 (upper) and ligand **1** (lower) in MeOD. (B) HR-MS of ligand **1**. (C) Chemical structure of ligand **1**.

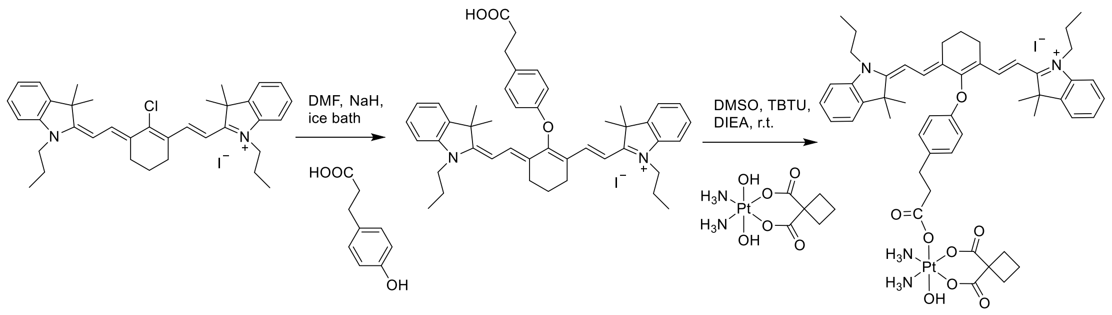

IR780 Ligand **1** ‘Cyaninplatin’

Fig. S2. Synthetic route of cyaninplatin.

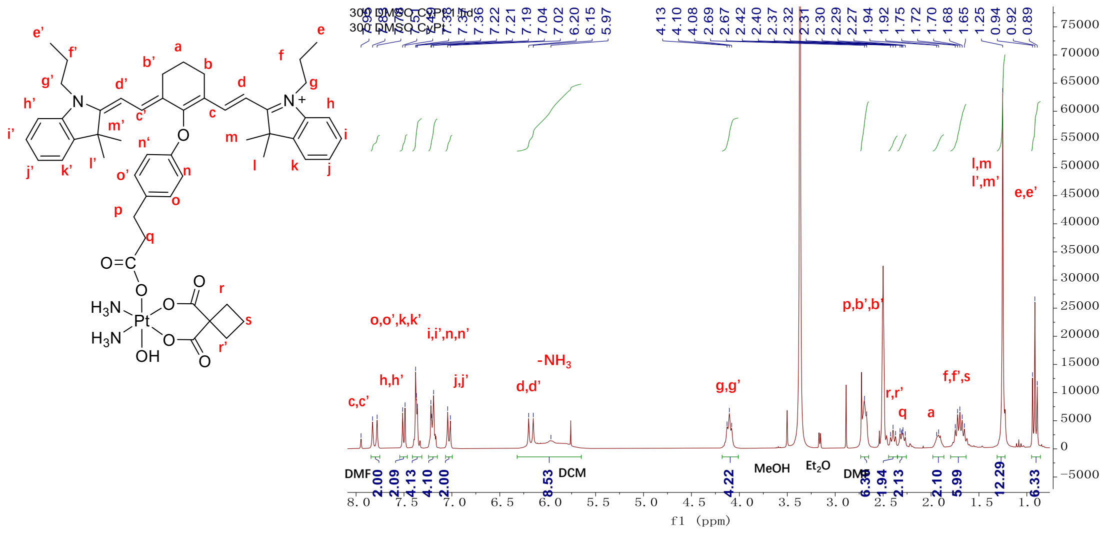

Fig. S3. ^{1}H NMR spectra of cyaninplatin in DMSO-d_6.

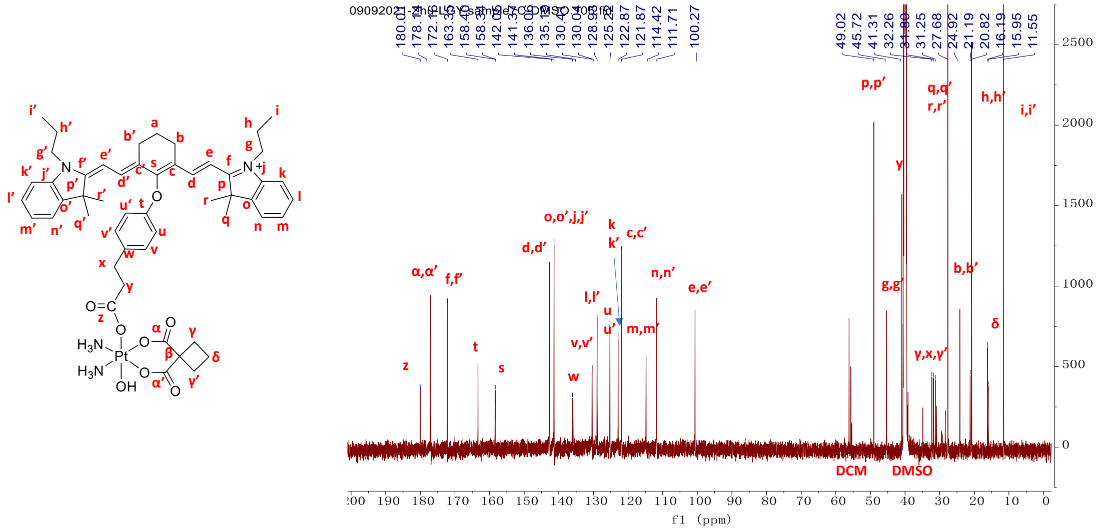

Fig. S4. ^{13}C NMR spectra of cyaninplatin in DMSO-d_6.

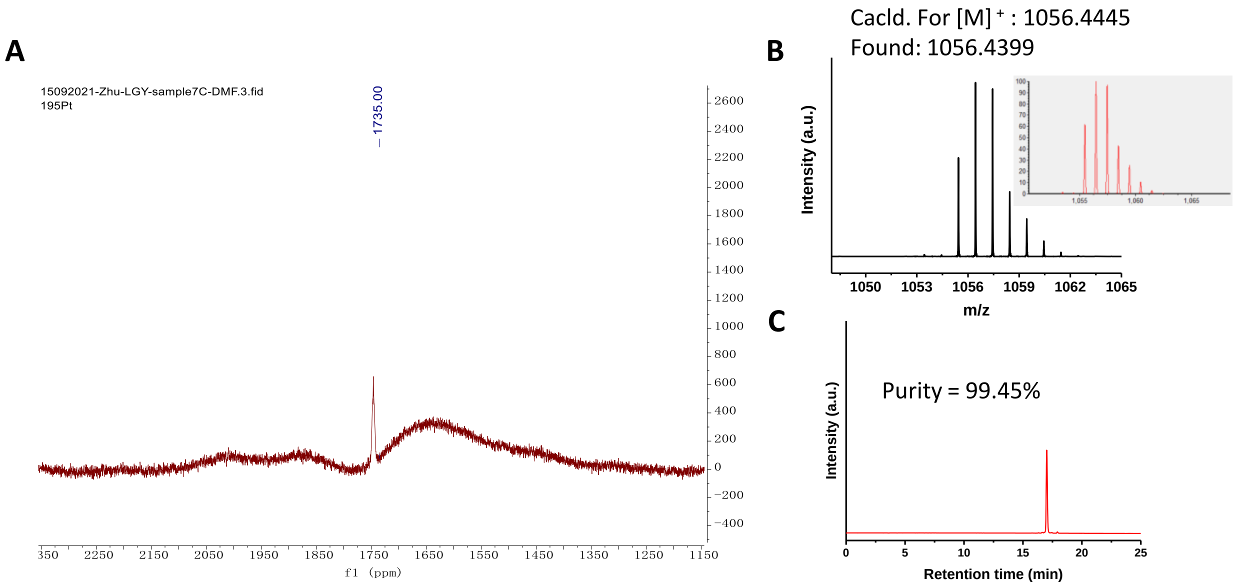

Fig. S5. (A) ^{195}Pt NMR spectra of cyaninplatin in DMF-d_7. (B) HR-MS of cyaninplatin. Insert: simulated MS spectra of cyaninplatin. (C) HPLC trace to check the purity of cyaninplatin.

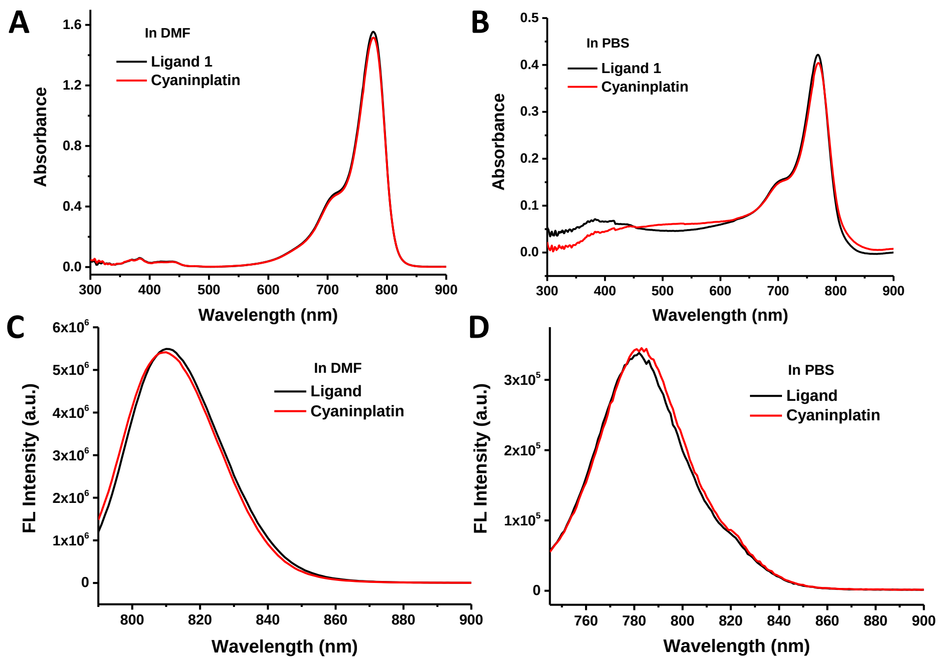

Fig. S6. Absorption spectra of ligand 1 and cyaninplatin in (A) DMF and (B) PBS. Fluorescent spectra of ligand 1 and cyaninplatin in (C) DMF (780 nm ex.) and (D) PBS (740 nm ex.).

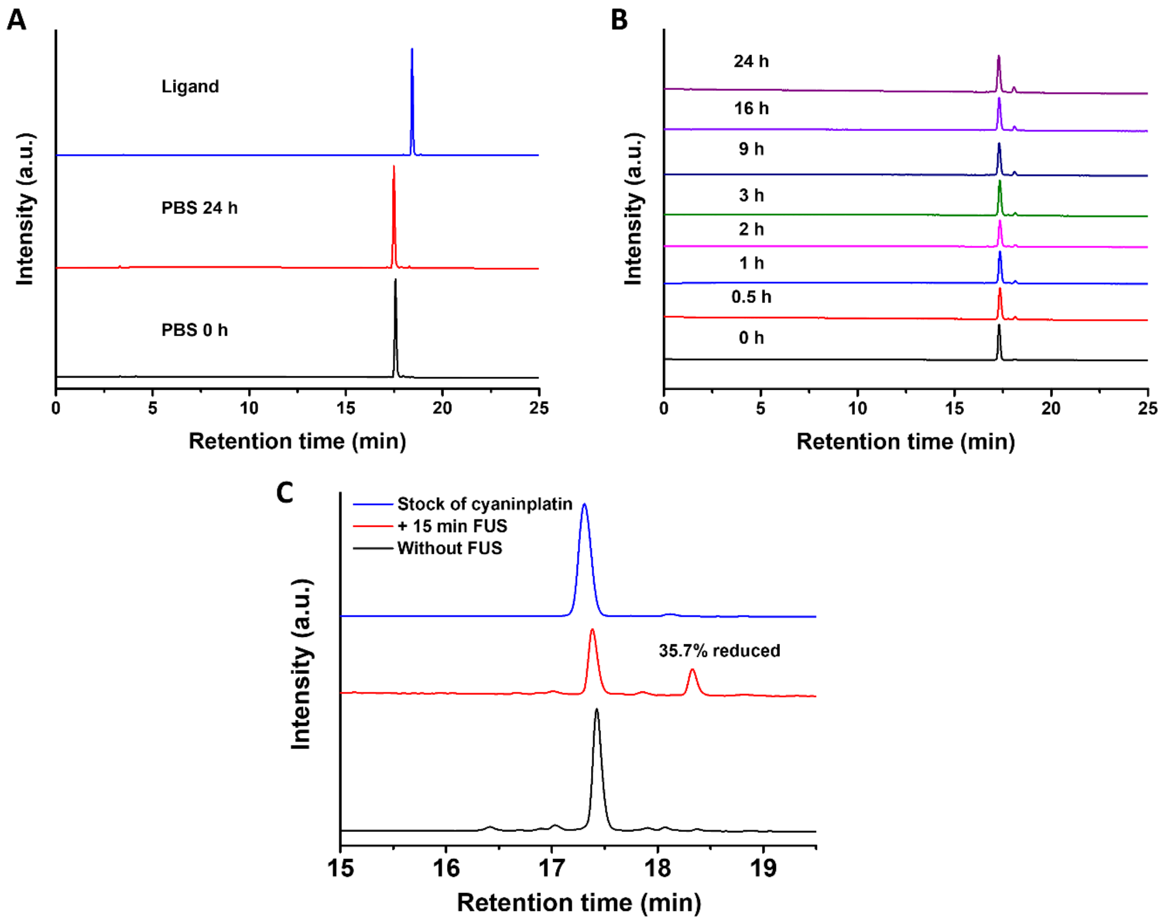

Fig. S7. (A) Stability of cyaninplatin in PBS buffer at 37 °C (1% DMF, pH 7.4). (B) Reduction profile of cyaninplatin in PBS buffer at 37 °C (1% DMF, pH 7.4) containing 5 mM ascorbate. (C) FUS-boosted reduction of cyaninplatin in fresh cell lysates (1% DMF, FUS: 3.5 W, 15 min).

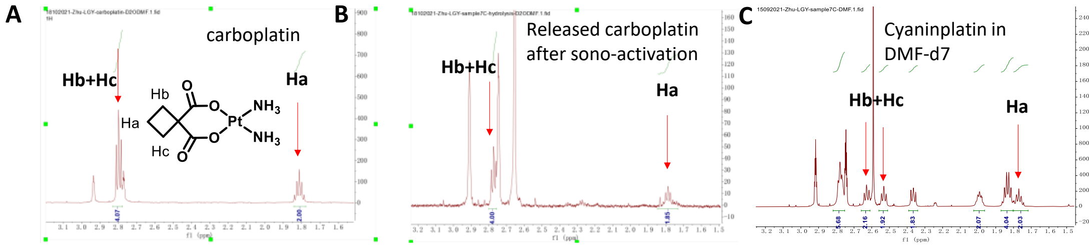

Fig. S8. ^{1}H NMR spectra of (A) carboplatin in 20% DMF-d_7 / 80% D_2O, (B) cyaninplatin after sono-activation (4 W, 60 min) in 20% DMF-d_7/ 80% D_2O and (C) before sono-activation in DMF-d_7. Due to the different chemical environments, Hb and Hc split into two individual peaks for Pt(IV) complexes containing asymmetric axial ligands. However, for carboplatin, only one group of peaks of Hb and Hc can be found.

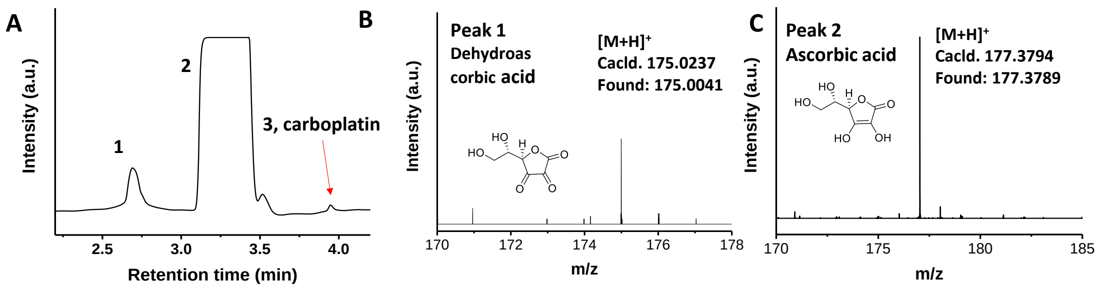

Fig. S9. (A) The LC-MS trace corresponding to Figure 1F, with UV detector at 220 nm. (B) The HRMS spectrum of dehydroascorbic acid (DHA) for peak 1; and (C) HRMS spectrum of ascorbic acid for peak 2.

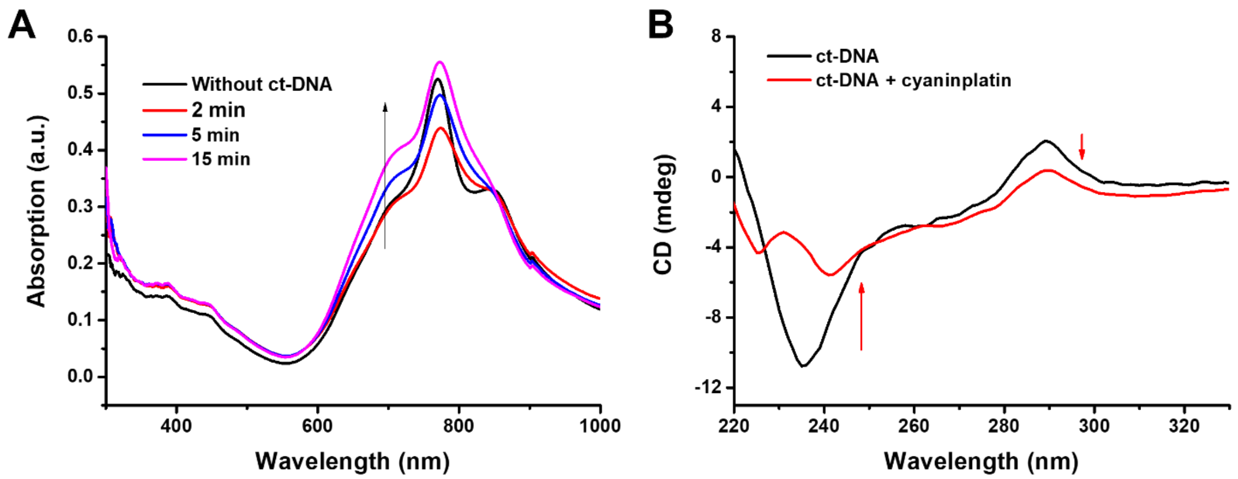

Fig. S10. (A) Absorption spectra of cyaninplatin (20 μM) incubated with ctDNA (150 μg mL^{-1}). (B) CD spectra of ctDNA incubated with cyaninplatin for 20 min.

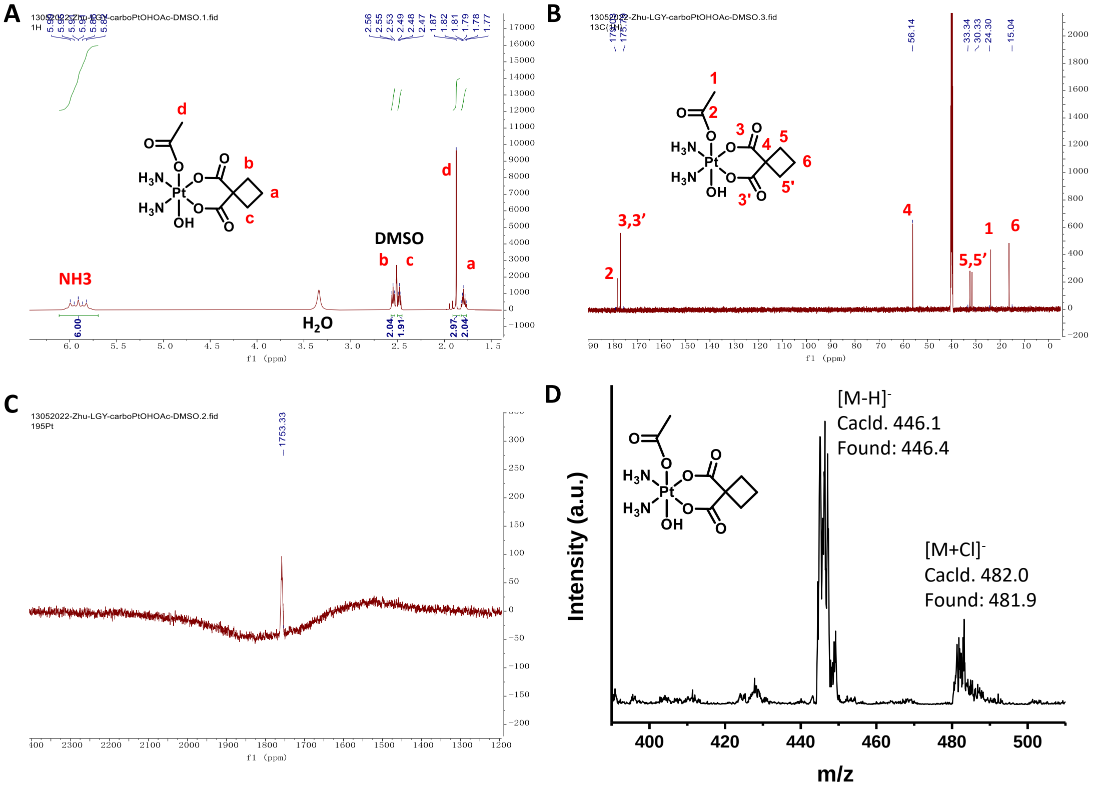

Fig. S11. (A) ^{1}H NMR, (B) ^{13}C NMR, and (C) ^{195}Pt NMR spectra of complex **2** in DMSO-d_6. (D) ESI-MS spectra of complex **2**.

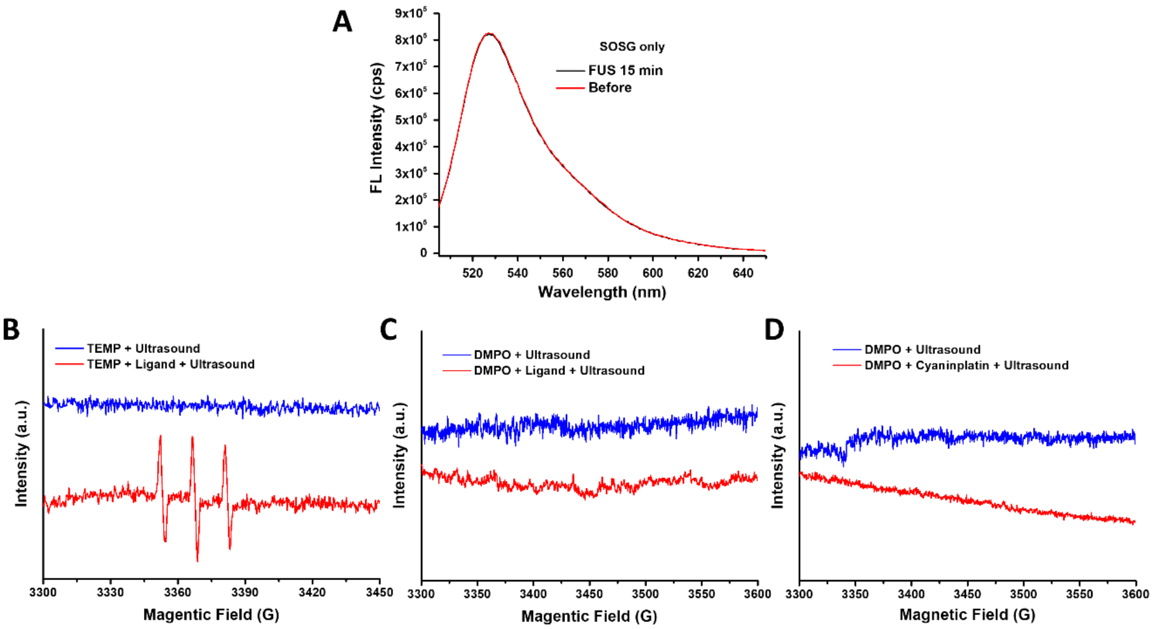

Fig. S12. (A) Fluorescence spectra of SOSG treated with FUS (4 W, 15 min). EPR spectra of (B) TEMP treated with ligand **1** and FUS (4 W, 15 min). (C) DMPO treated with ligand **1** and FUS, and (D) DMPO treated with cyaninplatin and FUS.

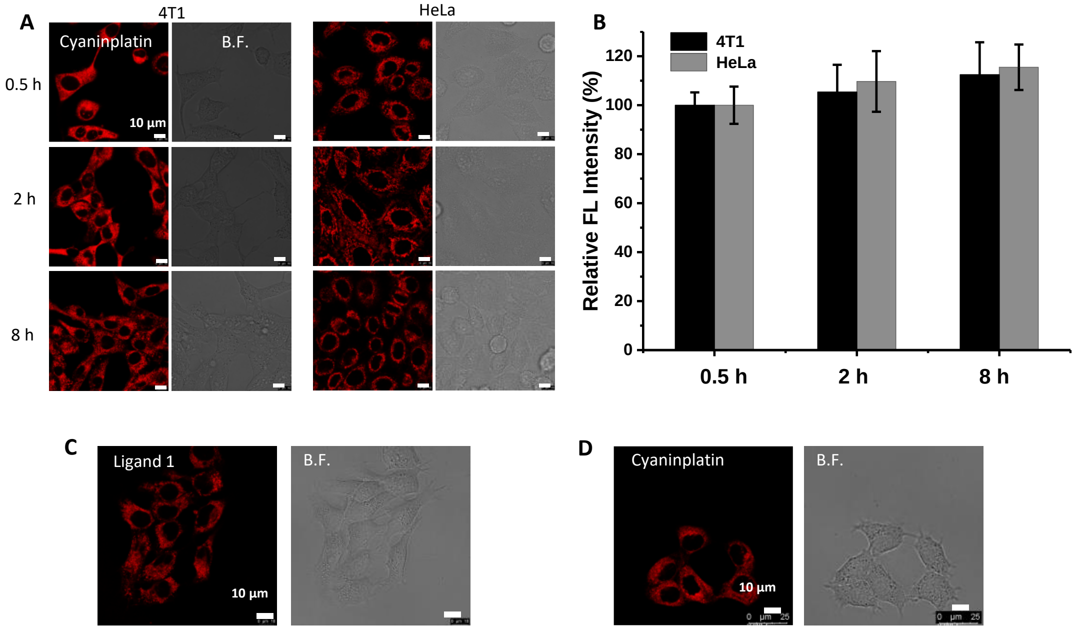

Fig. S13. (A) Cellular accumulation of cyaninplatin (drug feeding: 12.5 μM for 0.5, 2, and 8 h) observed by confocal laser scanning microscopy (CLSM) in 4T1 and HeLa cell lines, and (B) Corresponding quantitative data. CLSM images for 4T1 cells treated with (C) ligand 1 (12.5 μM, 30 min) and (D) cyaninplatin (12.5 μM, 30 min). Mean ± SD.

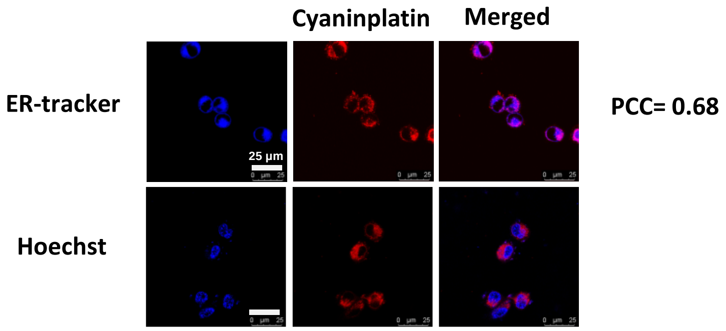

Fig. S14. Subcellular distribution of cyaninplatin in 4T1 cells after treatment with cyaninplatin (12.5 μM, 30 min).

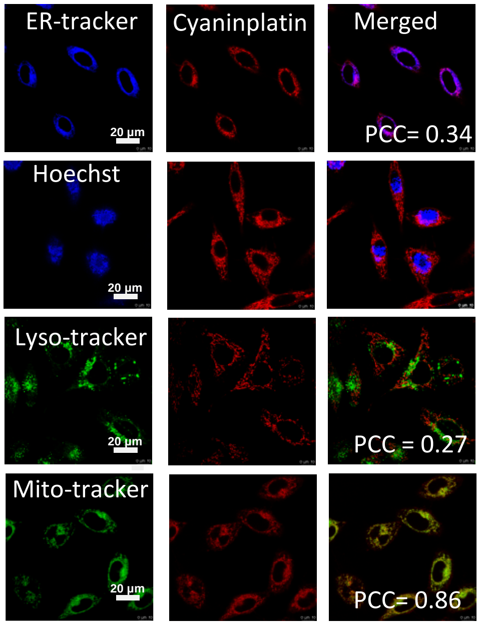

Fig. S15. Subcellular distribution of cyaninplatin in HeLa cells after treatment with cyaninplatin (12.5 µM, 30 min).

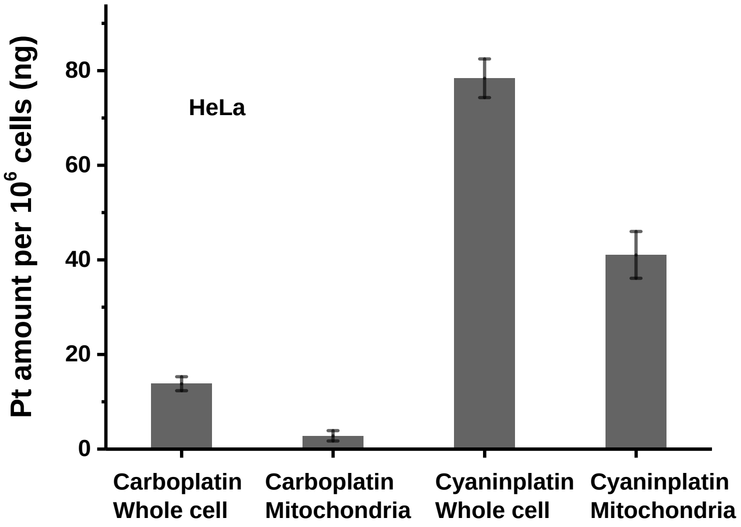

Fig. S16. ICP analysis of extracted mitochondrial parts of cyaninplatin-treated (12.5 μM, 30 min) HeLa cells. Mean ± SD, *n* =3.

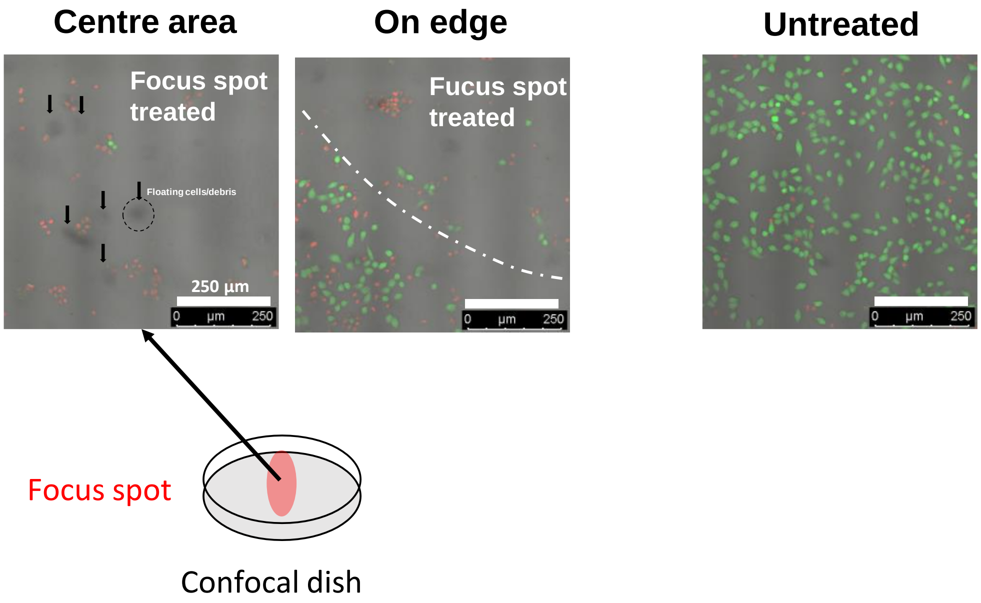

Fig. S17. Calcein-AM/PI double staining for 4T1 cells treated with cyaninplatin (12.5 µM, 30 min) and FUS (1.75 MHz, 2 W, 10 min) in confocal dishes; the dimension of focus point: geometric length: 7.34 mm, diameter: 0.8 mm. Due to mechanical disturbance caused by focused ultrasound to adherent cells, the cells that directly contacted the focus area of FUS became suspension immediately. Thus, the evaluation on cytotoxicity based on this lab-built FUS device (1.75 MHz) were conducted using resuspended cells unless specified otherwise.

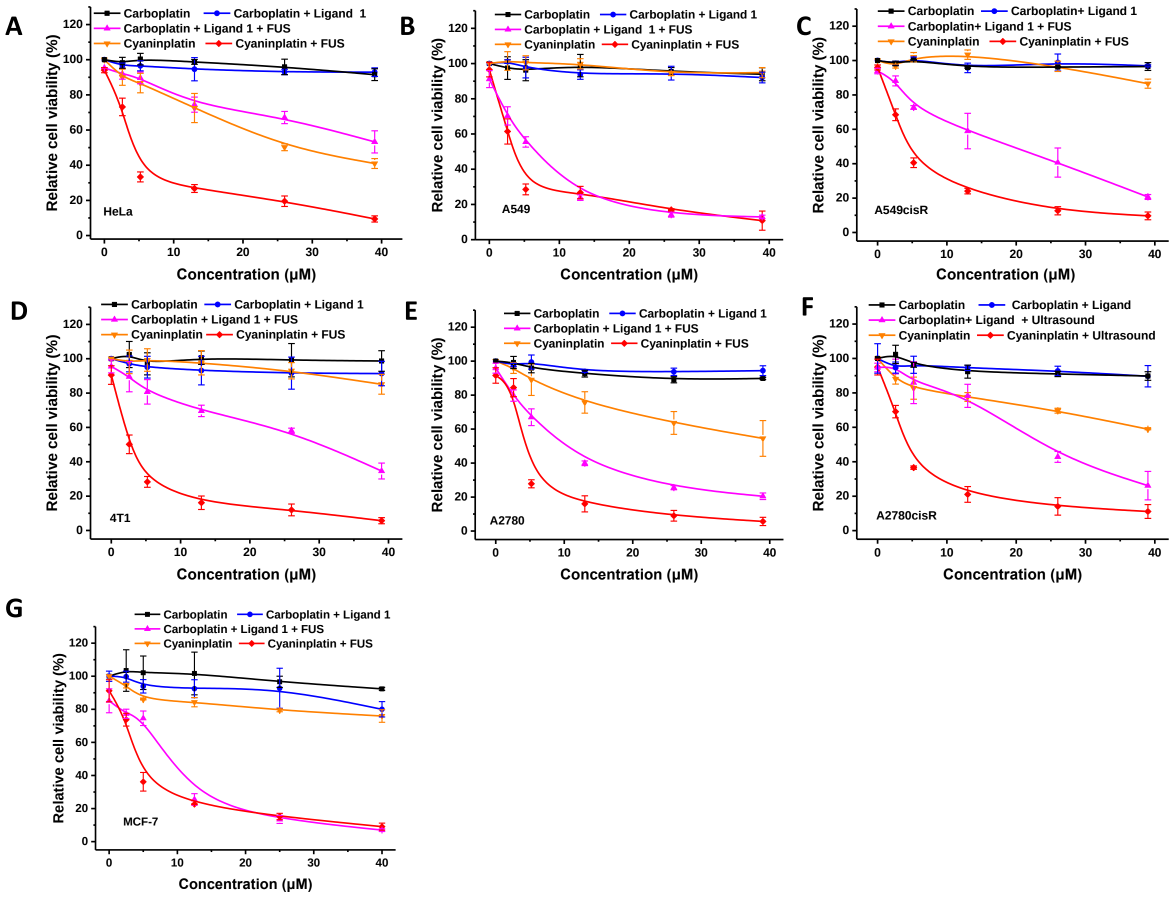

Fig. S18. Relative cell viability of treated cells under different conditions on various carcinoma cell lines. (A) HeLa; (B) A549; (C) A549cisR; (D) 4T1; (E) A2780; (F) A2780cisR; (G) MCF-7. Viability of cells treated with vehicle solution (0.5% DMF) only was defined as 100%. Drug feeding time: 30 min, FUS: 3.5 W, 15 min, cell viability tested at 24 h post-treatment. Mean ± SD, n = 3.

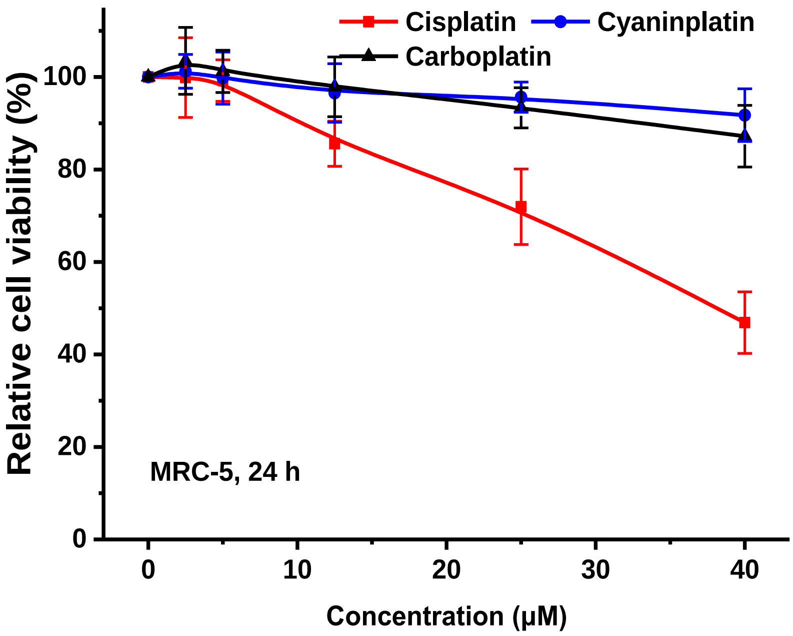

Fig. S19. Cell viability of cisplatin, carboplatin, and cyaninplatin against MRC-5 cells, with the treatment time of 24 h. Mean ± SD, $n = 3$.

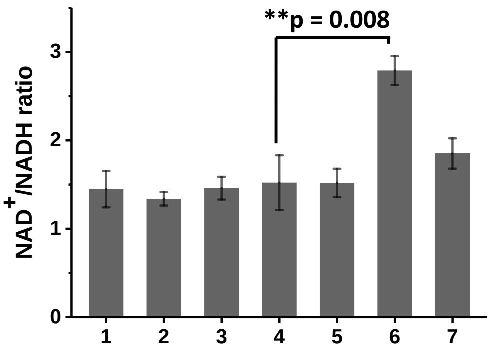

Fig. S20. NAD$^+$/NADH ratio of 4T1 cells under different treatment conditions: 1. Vehicle control (0.5% DMF); 2. FUS (3.5 W, 10 min); 3. Ligand **1** + carboplatin (25 μM); 4. Ligand **1** + carboplatin + FUS; 5. Cyaninplatin (25 μM); 6. Cyaninplatin + FUS; 7. H_2O_2 (100 μM, 24 h). Mean ± SD, *n* = 3, *t*. test, $^{**}p < 0.01$.

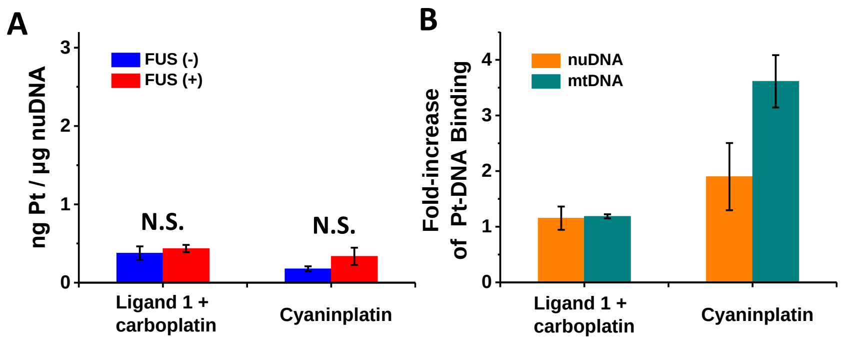

Fig. S21. (A) Pt-binding level of nuclear DNA of 4T1 cells treated with carboplatin + ligand **1** or cyaninplatin (12.5 μM, 30 min) with FUS (3.5 W, 15 min). (B) Fold-increase of Pt binding level to nuclear DNA and mitochondrial DNA for cells after activation by FUS (3.5 W, 15 min). Mean ± SD, *n* =3, *t.* test, N.S. not significant.

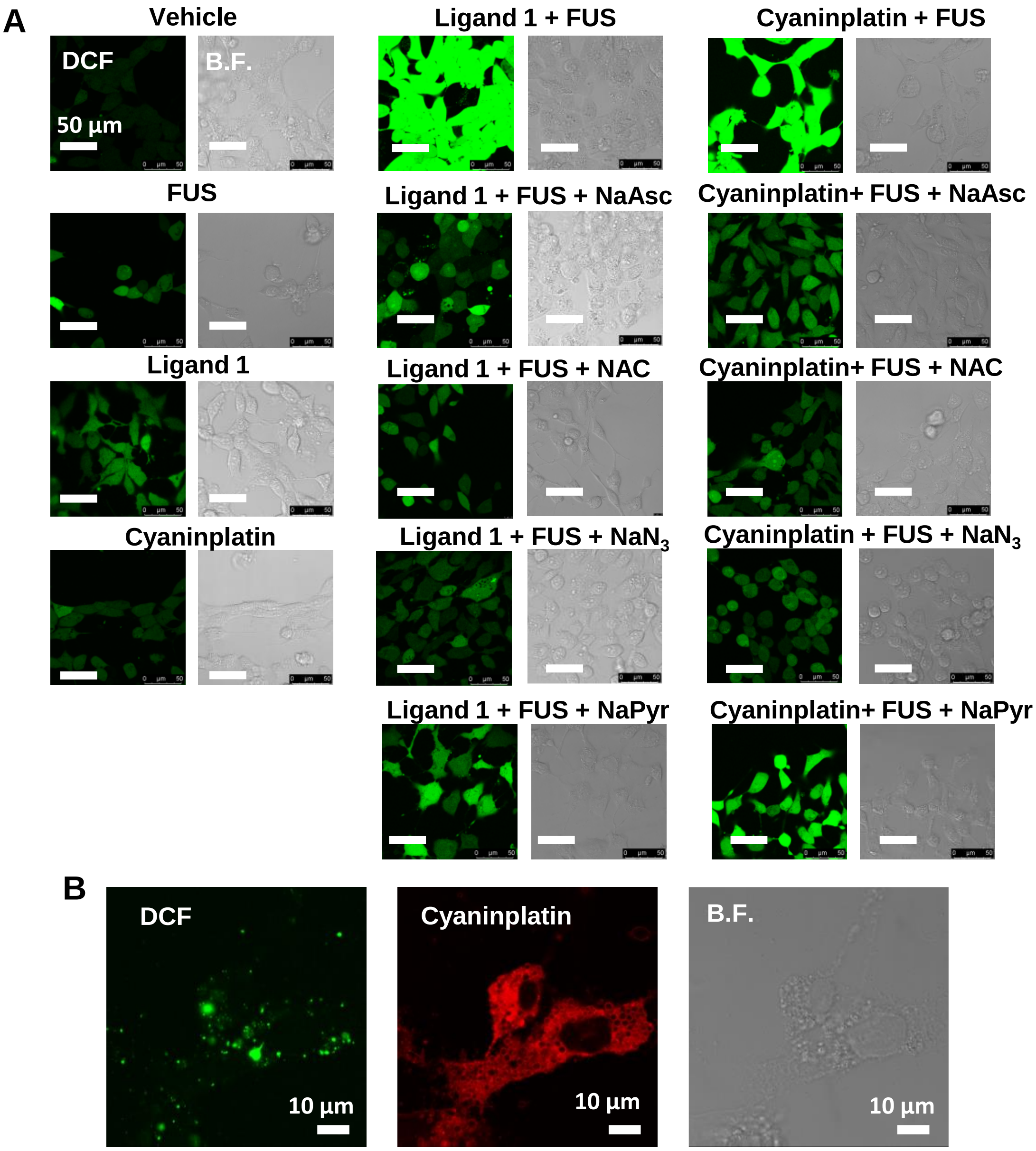

Fig. S22. (A) DCFH-DA staining for intracellular ROS of 4T1 cells pretreated with various ROS scavengers. Ligand **1** (35 μM, 30 min), cyaninplatin (10 μM, 30 min), FUS: 3 MHz, 3.5 W, 15 min. (B) Formation of intracellular vacuoles in 4T1 cells pretreated with ascorbate at 6 h post-treatment by FUS-activated cyaninplatin.

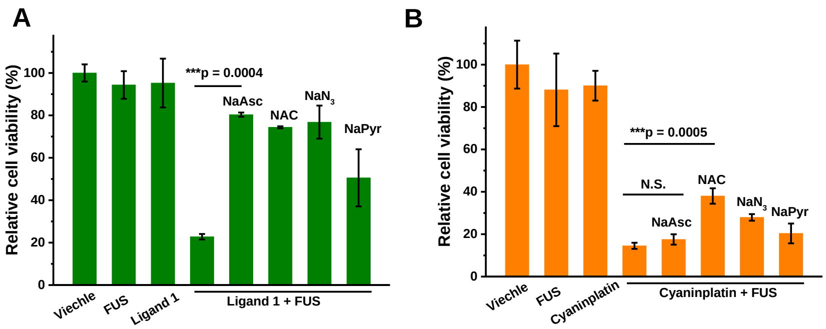

Fig. S23. Cytotoxicity with pretreatment by antioxidants for (A) ligand **1** (35 μM, 30 min) and (B) cyaninplatin (10 μM, 30 min), FUS: 3MHz, 3.5 W, 15 min. Mean ± SD, n = 3, t. test, ***p < 0.001.

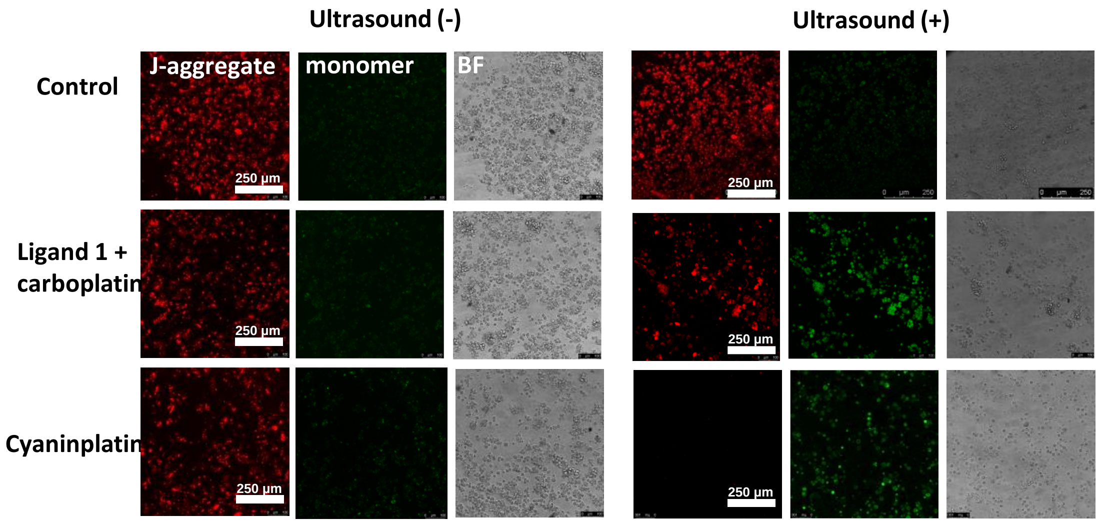

Fig. S24. JC-1 staining of 4T1 cells under different treatment conditions. FUS condition: 3.5 W, 5 min. Cyaninplatin: 25 μM, 30 min.

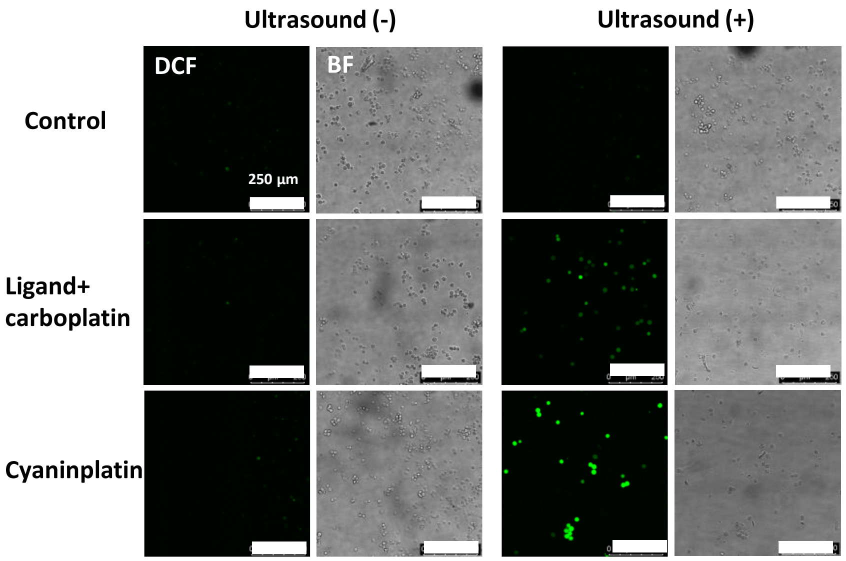

Fig. S25. DCFH-DA staining of 4T1 cells under different treatment conditions. FUS condition: 3.5 W, 10 min. Cyaninplatin: 25 µM, 30 min.

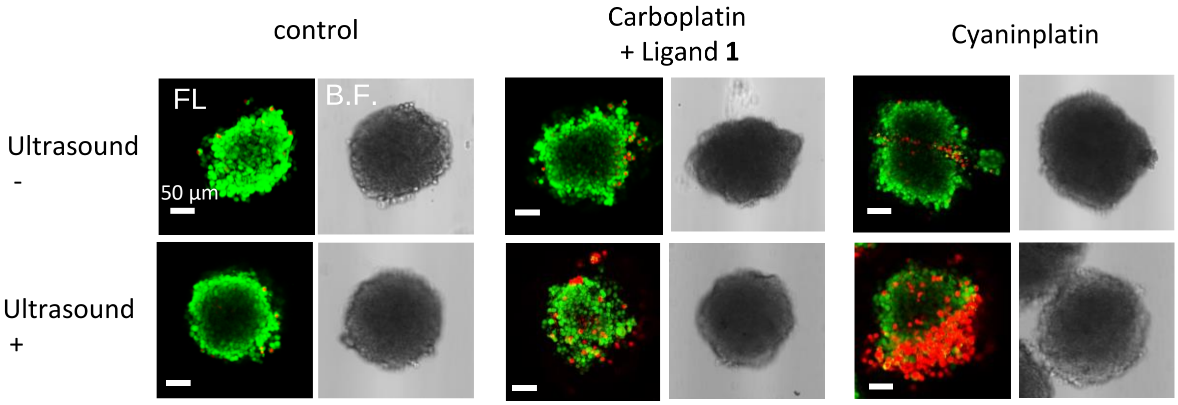

Fig. S26. Calcein-AM/PI double staining of 4T1 tumor spheroids receiving different treatments. FUS condition: 3.5 W, 15 min. Drug feeding for mixture or cyaninplatin: 25 µM, 30 min (scale bar: 50 µm).

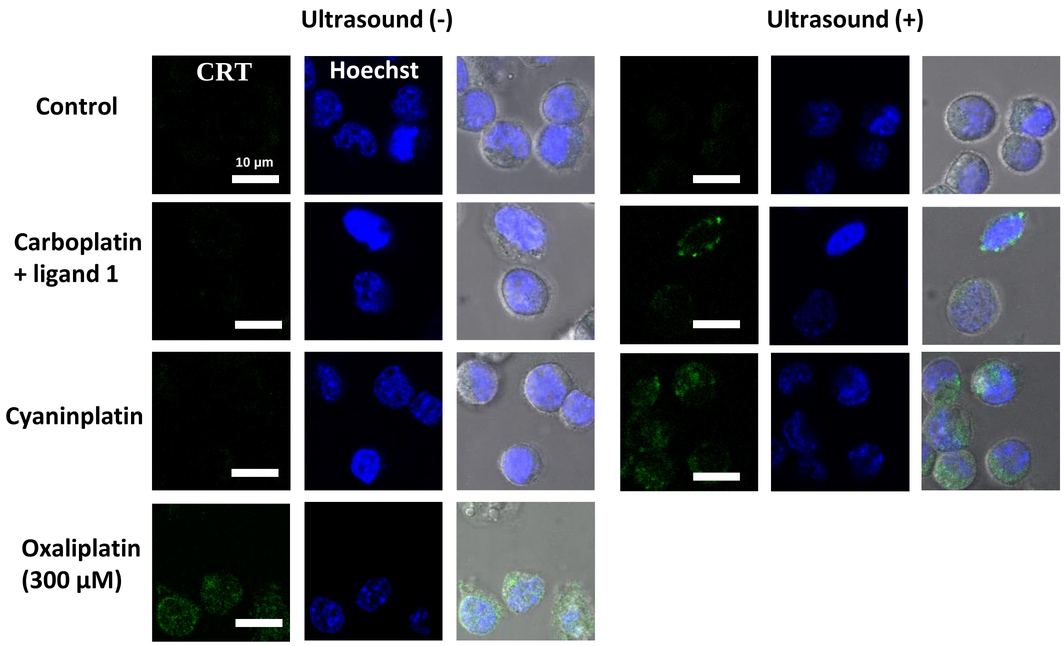

Fig. S27. CLSM images of 4T1 cells under different treatment conditions and stained for exposure of CRT. FUS condition: 3.5 W, 10 min. Cyaninplatin: 25 µM, 30 min.

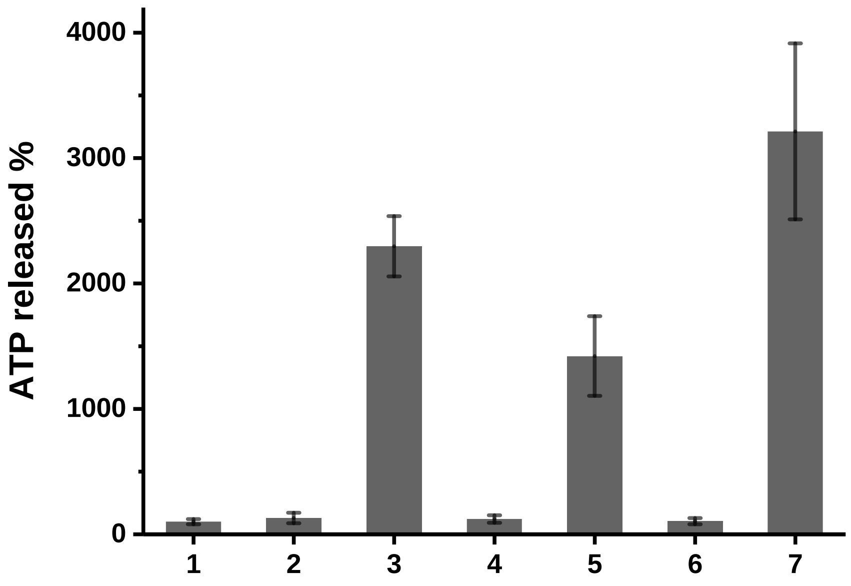

Fig. S28. Relative level of ATP released to extracellular medium of 4T1 cells received different treatments: 1) Vehicle control (0.5% DMF); 2) FUS (3.5 W, 10 min) only; 3) Oxaliplatin 300 μM for 3 h; 4) Carboplatin + ligand **1** (25 μM, 30 min); 5) Carboplatin + ligand **1** + FUS; 6) Cyaninplatin (25 μM, 30 min); and 7) Cyaninplatin + FUS. The level from the control group was deemed as 100%. Mean ± SD, $n = 3$.

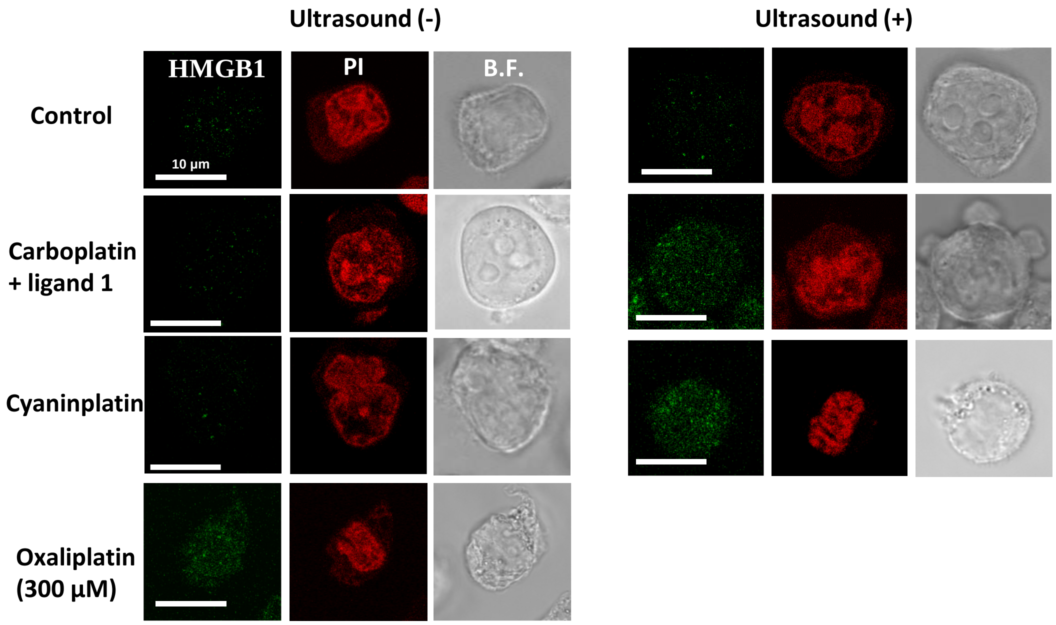

Fig. S29. CLSM images of 4T1 cells under different treatment conditions and stained for release of HMGB1. FUS condition: 3.5 W, 10 min. Cyaninplatin: 25 μM, 30 min.

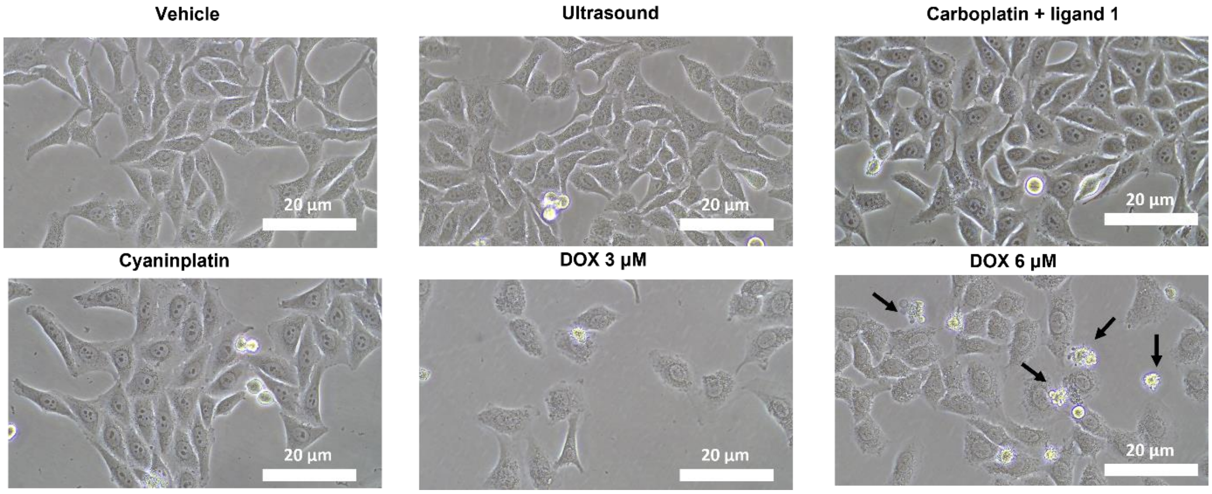

Fig. S30. Bright field microscopy images for cells received different treatments. Vehicle: 0.5% DMF; ultrasound: 3 MHz, 3.5 W, 15 min; carboplatin + ligand **1**: equivalent mixture of carboplatin and ligand **1** at 35 M for 30 min of drug feeding; cyaninplatin: 10 µM for 30 min; Doxorubicin (DOX): 3 or 6 µM for 4 h of treatment, typical morphology of late apoptosis was highlighted.

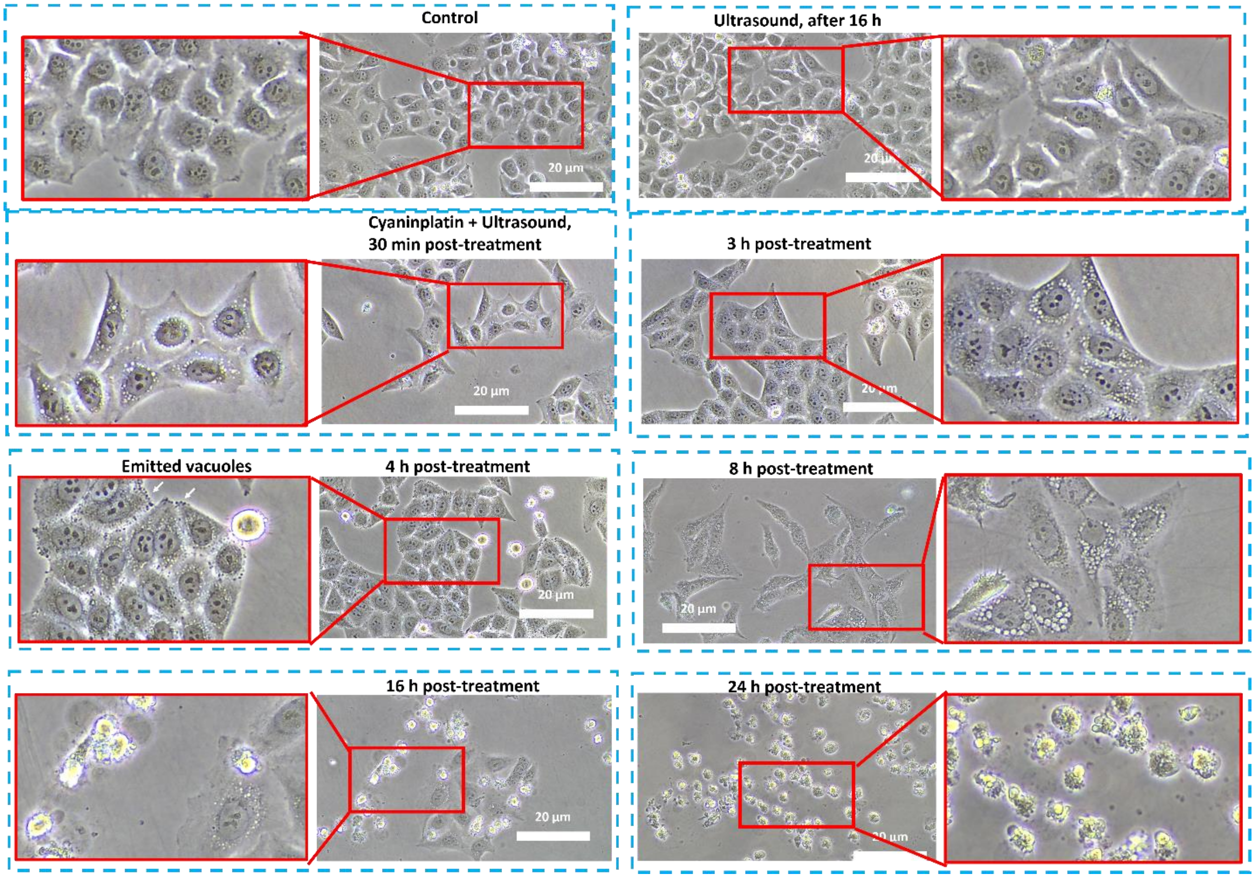

Fig. S31. Bright field images for HeLa cells at different time points post treatment by cyaninplatin (drug feeding for 10 µM for 30 min) and ultrasound exposure (3 MHz, 3.5 W, 15 min).

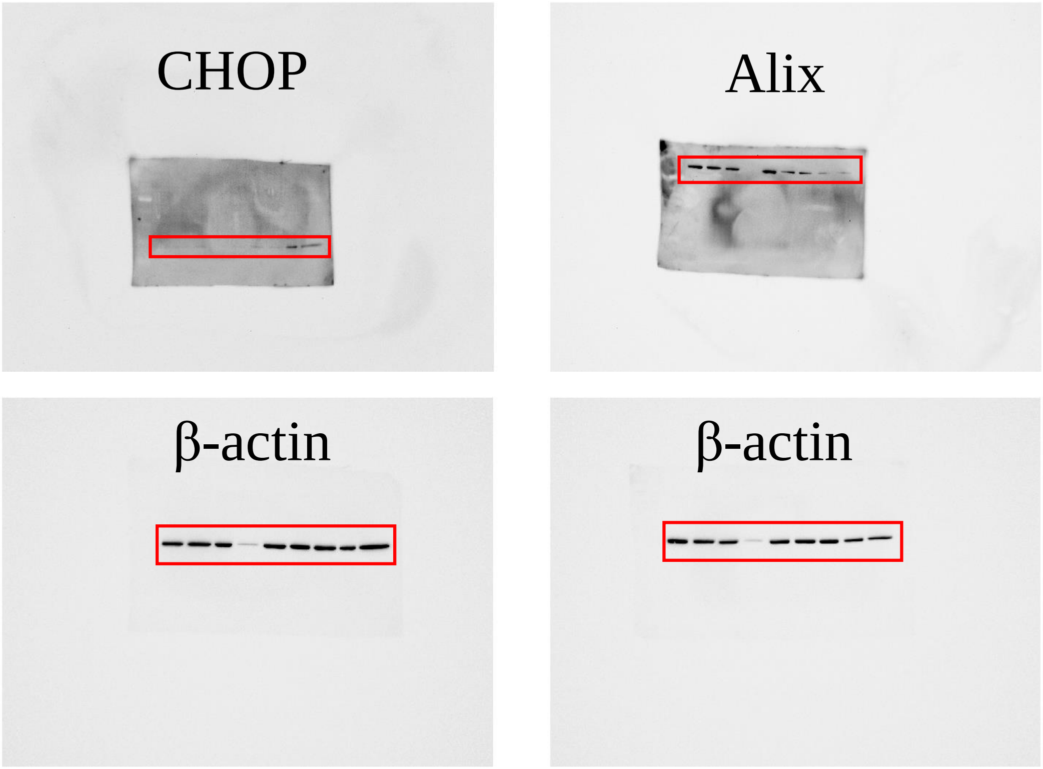

Fig. S32. Uncropped image corresponding to Fig. 5G.

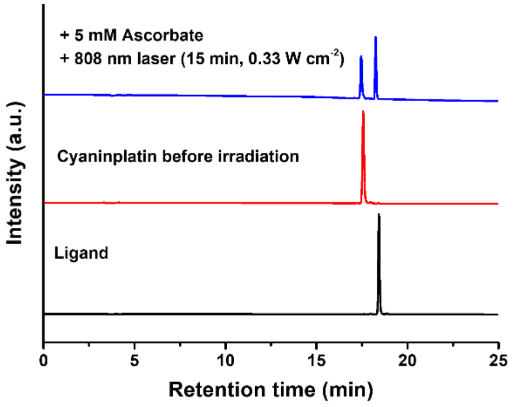

Fig. S33. Photo-activation of cyaninplatin by 808 nm laser at maximum permissible exposure limit (0.33 W cm^{-2}) for 15 min.

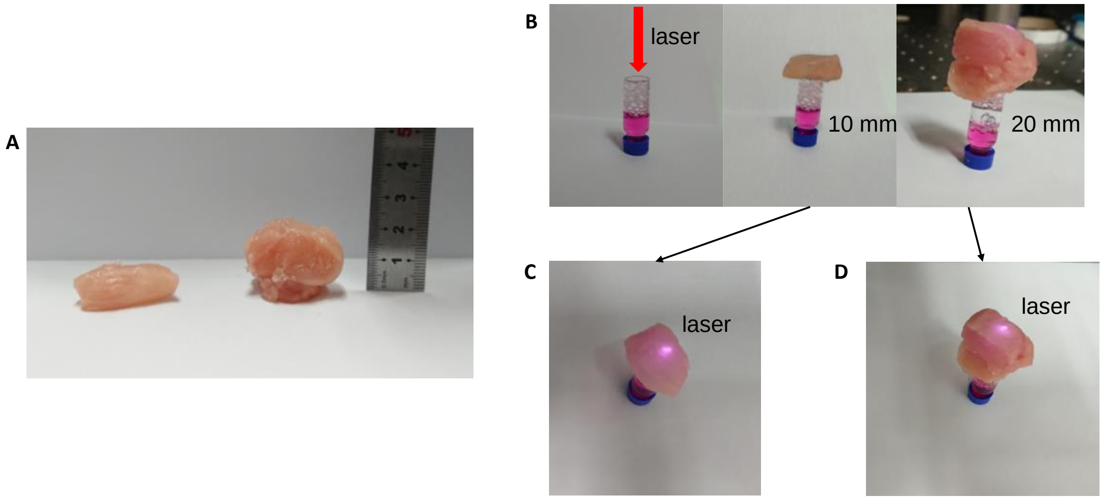

Fig. S34. Photos of (A) chicken breast tissues of different thicknesses to cover (B, C, and D) laser-treated suspension of 4T1 cells. The thickness was defined as the natural tissue that was not affected by outer press or strain forces.

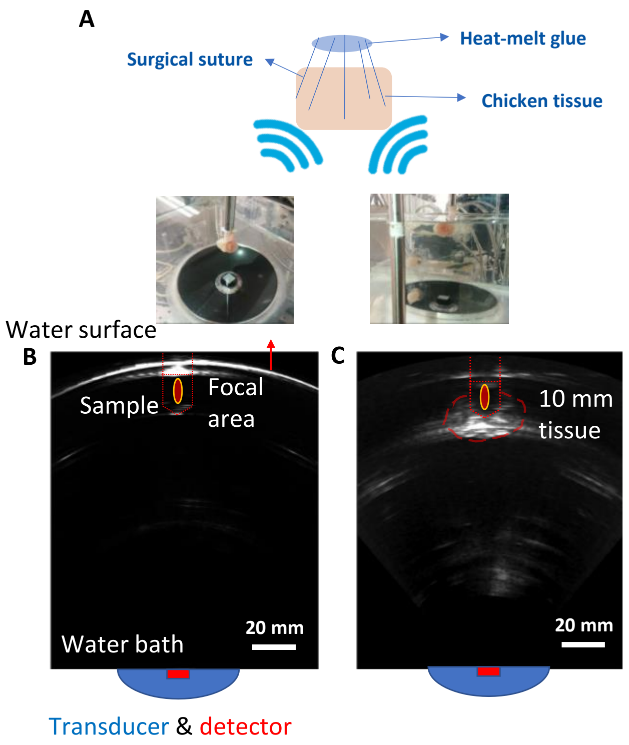

Fig. S35. (A) Device setup for treatment with FUS to penetrate chicken breast tissues. (B), and (C) Real-time ultrasound imaging to monitor the treatment process. FUS condition: 3.5 W, 15 min. The same tissue was used as in Figure S33, and the thickness of the tissues can be visualized by ultrasound imaging.

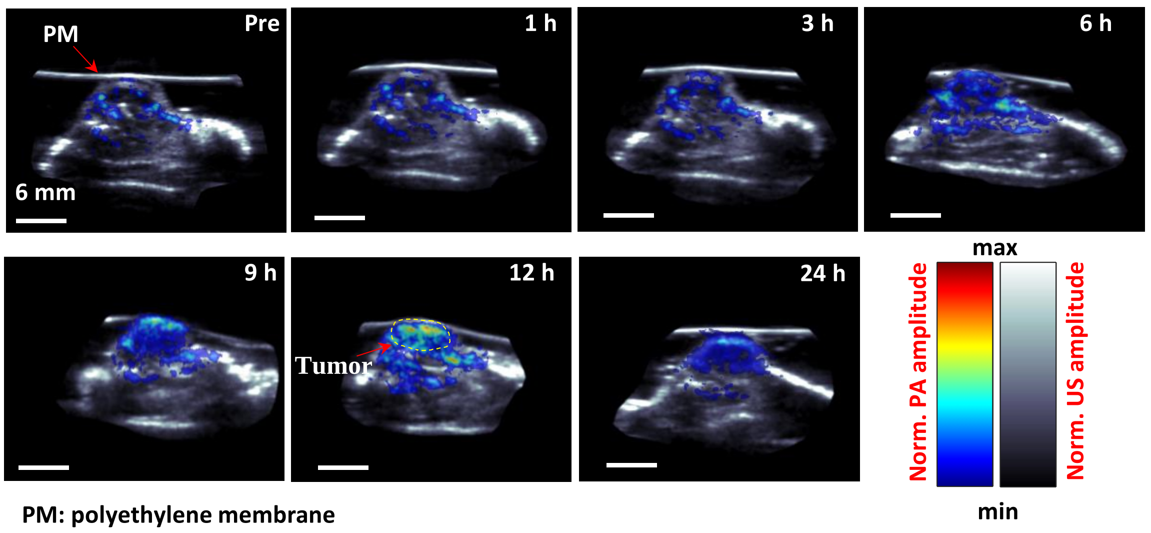

Fig. S36. Time-dependent B-mode ultrasound/photoacoustic computed-tomography images of the tumor-bearing mouse.

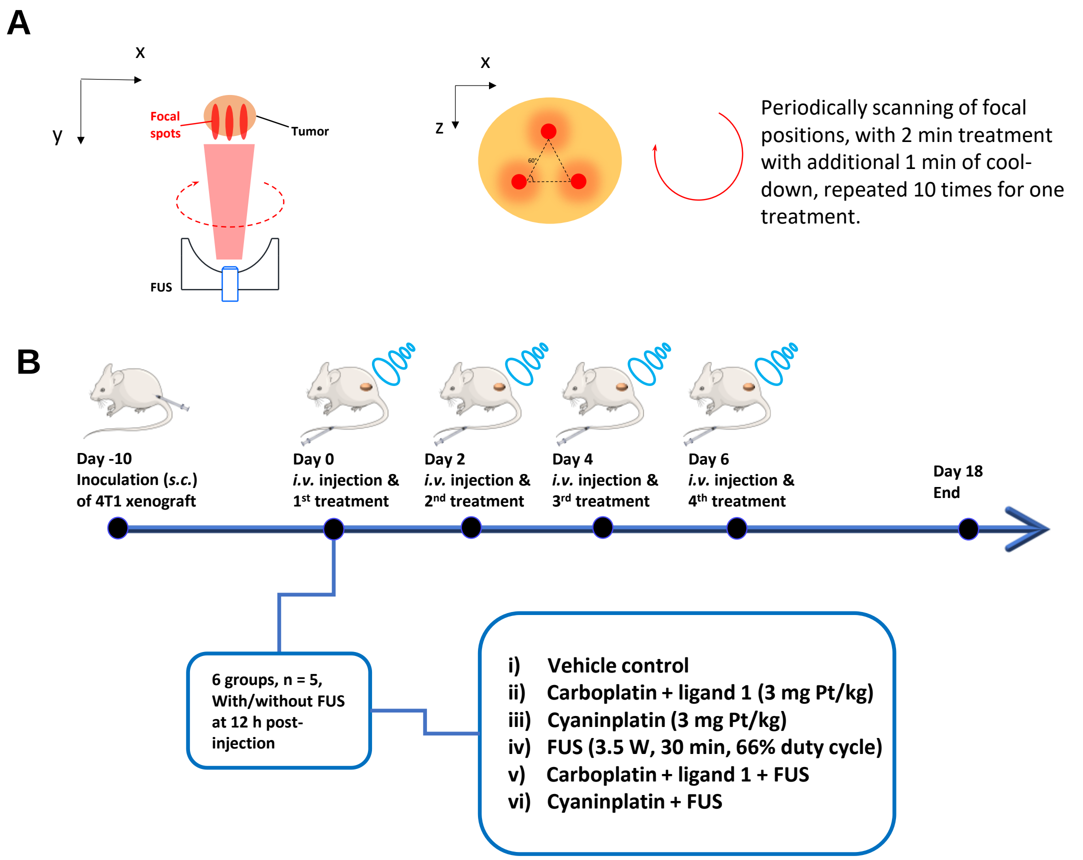

Fig. S37. Schematic illustration of (A) programmable-scanning focused ultrasound treatment of tumor and (B) treatment procedure of tumor-bearing mice.

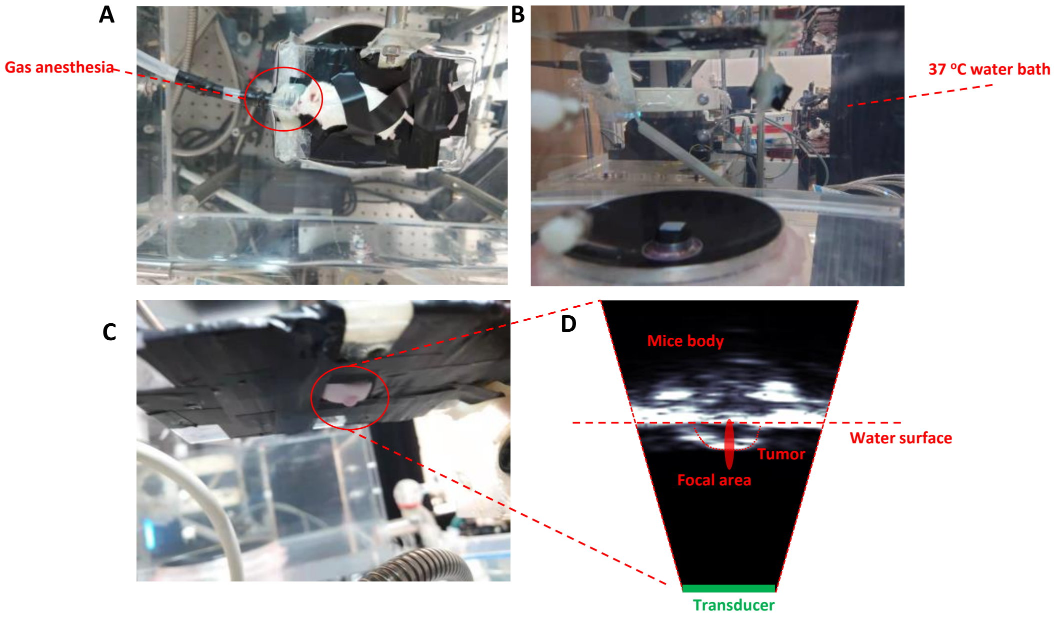

Fig. S38. Photos of (A) treatment platform for mice. (B) FUS with a temperature-controlled water bath. (C) Tumor of mice. And (D) Real-time ultrasound imaging to guide the application of the focal spot of FUS.

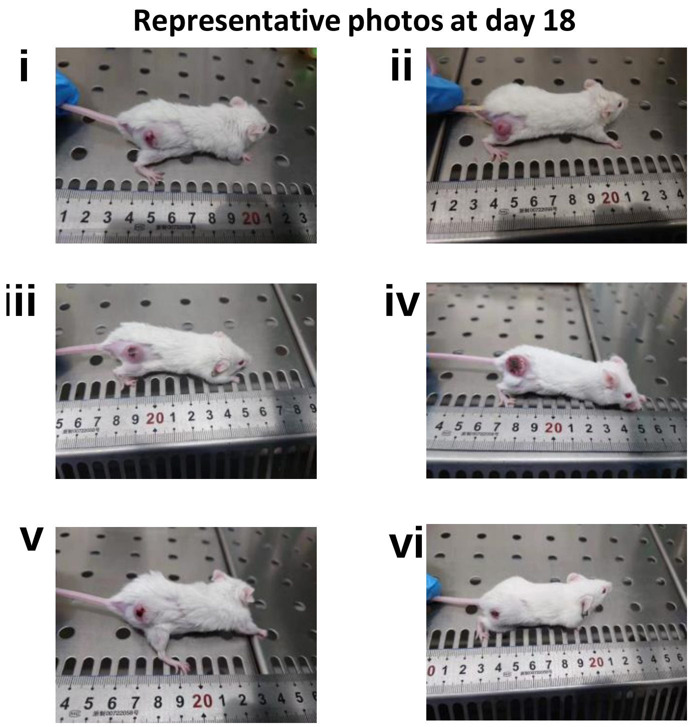

Fig. S39. Representative photos of mice from each group by the end of treatment.

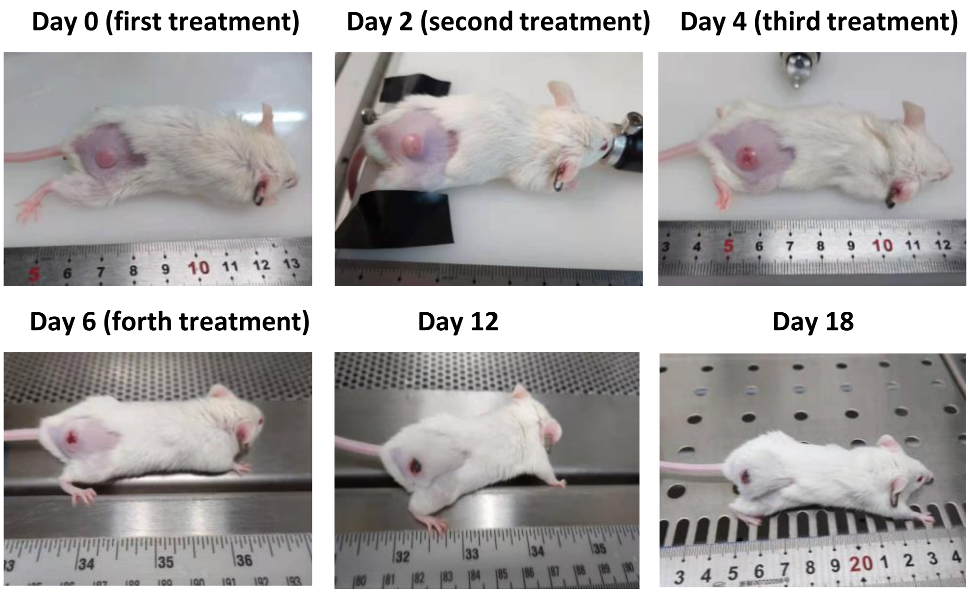

Fig. S40. Photos of tumor status in group vi during treatment.

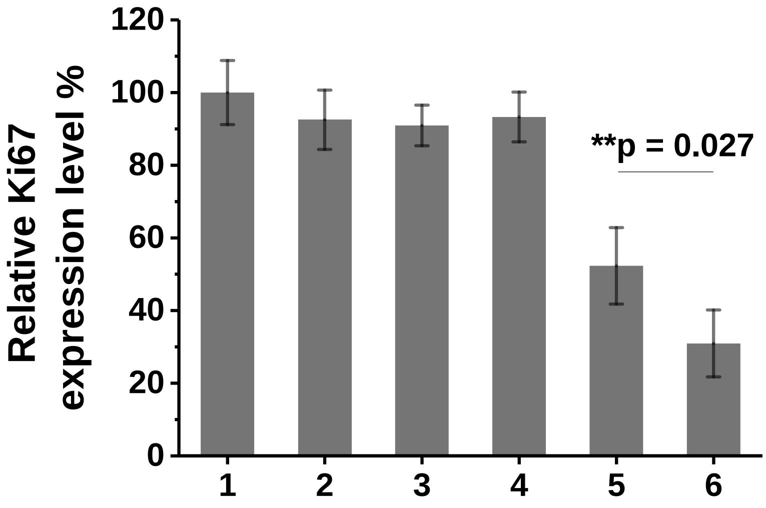

Fig. S41. Quantitative result of Ki67 staining of tumor slides by calculating relative area and gray value of Ki67-positive signals with ImageJ. (Mean ± SD, n = 5, t. test, $**p < 0.01$)

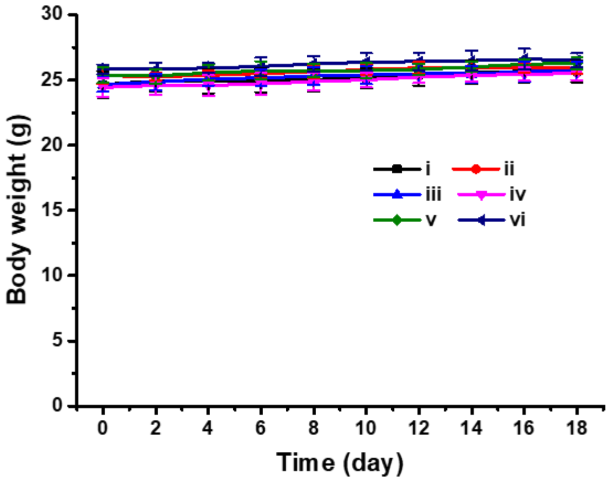

Fig. S42. Body weight of mice during treatment. Mean ± SD, *n* = 5.

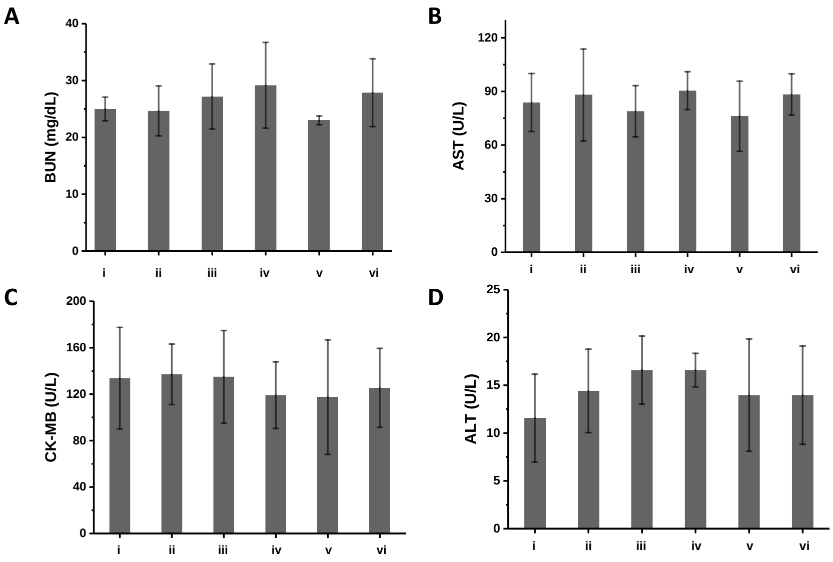

Fig. S43. Blood chemistry assay of mice after treatment. (A) Blood urea nitrogen (BUN); (B) Aspartate aminotransferase (AST); (C) Creatine kinase-myocardial band (CK-MB); (D) Alanine aminotransferase (ALT). Mean ± SD, n = 5.

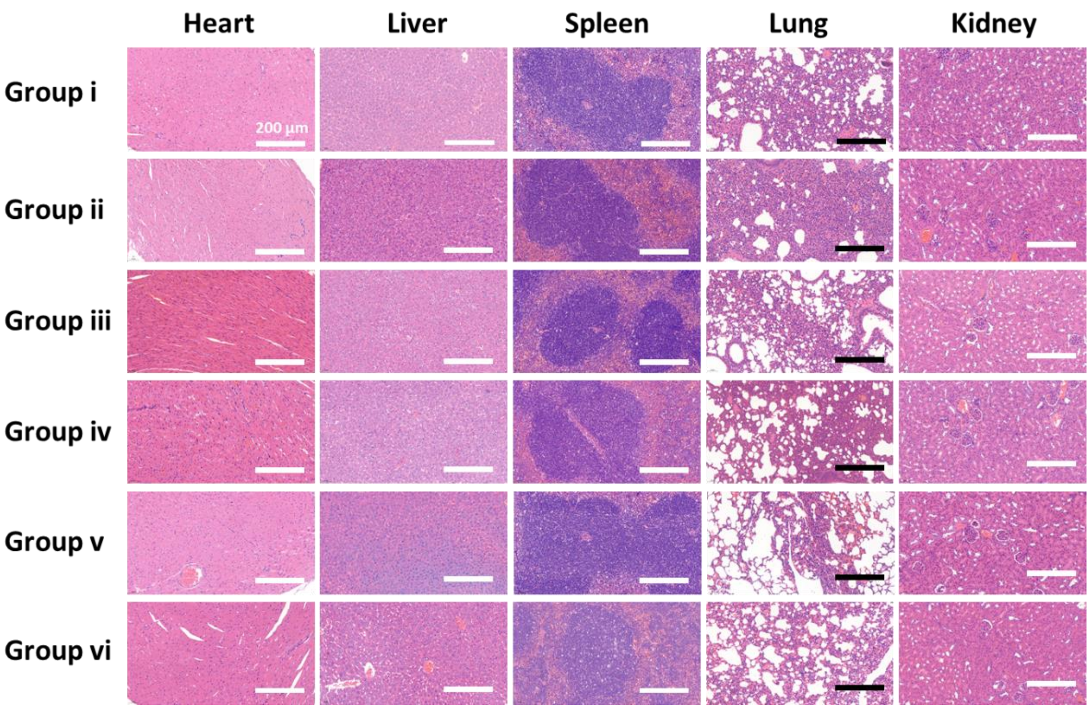

Fig. S44. H&E staining for main organ tissues of mice in each group after treatment.

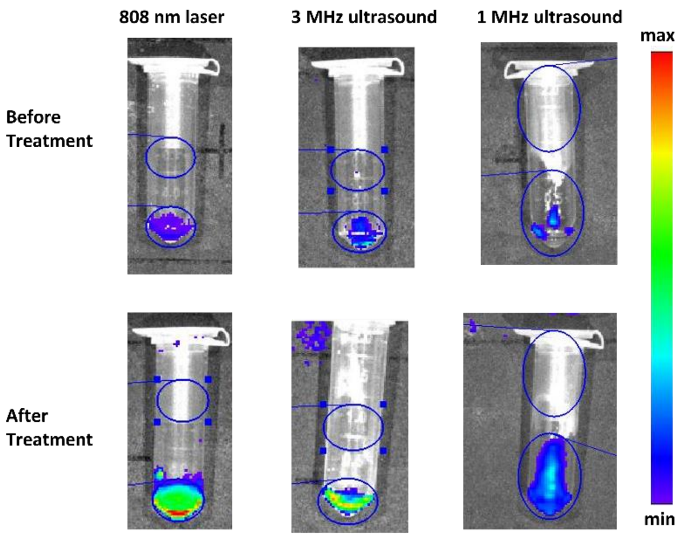

Fig. S45. Solution of SOSG (20 μM) and cyaninplatin (15 μM) exposed to different activation sources. 808 nm laser (0.33 W cm^{-2}, 10 min), FUS (1 or 3 MHz, 3.5 W, 10 min).

Table S1. IC_{50} value (μM) in various carcinoma cell lines. [a]Mixture: mixture of equivalent carboplatin and ligand **1**. Drug feeding: 30 min, FUS condition: 3.5 W, 15 min, cell viability tested at 24 h post-treatment. [b]Sono-sensitization Index (SI): defined as the IC_{50} of ultrasound-activated cyaninplatin / IC_{50} of cyaninplatin without ultrasound activation. [c]RF: defined as the IC_{50} in platinum-resistant cell lines (A2780cisR or A549cisR) / the IC_{50} in platinum-sensitive cell lines (A2780 or A549). Mean ± SD, *n* = 3.

Cell line	Carboplatin	Mixture[a]	Mixture + FUS	Cyaninplatin	Cyaninplatin + FUS	SI[b]
HeLa	> 40	> 40	> 40	26.3±2.8	4.1±0.2	6.4
MCF-7	> 40	> 40	8.8±0.6	> 40	4.2±0.3	>9.5
A2780	> 40	> 40	10.1±0.8	> 40	4.0±0.2	>10.0
A2780-cisR	> 40	> 40	23.4±1.4	> 40	4.1±0.2	>9.8
RF[c]			2.3		1.0	
A549	> 40	> 40	6.6±0.7	> 40	3.5±0.5	>11.4
A549-cisR	> 40	> 40	19.4±6.7	> 40	4.3±0.3	>9.3
RF			2.9		1.2	
4T1	> 40	> 40	30.5±1.5	> 40	2.0±0.7	>20
